# Novel Mixed NOP/Opioid Receptor Peptide Agonists

**DOI:** 10.1021/acs.jmedchem.0c02062

**Published:** 2021-05-17

**Authors:** Salvatore Pacifico, Valentina Albanese, Davide Illuminati, Erika Marzola, Martina Fabbri, Federica Ferrari, Victor A.D. Holanda, Chiara Sturaro, Davide Malfacini, Chiara Ruzza, Claudio Trapella, Delia Preti, Ettore Lo Cascio, Alessandro Arcovito, Stefano Della Longa, Martina Marangoni, Davide Fattori, Romina Nassini, Girolamo Calò, Remo Guerrini

**Affiliations:** † Department of Chemical, Pharmaceutical and Agricultural Sciences, University of Ferrara, Via Luigi Borsari 46, Ferrara 44121, Italy; ‡ Department of Neuroscience and Rehabilitation, Section of Pharmacology, University of Ferrara, Via Fossato di Mortara 17/19, Ferrara 44121, Italy; § Technopole of Ferrara, LTTA Laboratory for Advanced Therapies, via Fossato di Mortara 70, Ferrara 44121, Italy; ∥ Dipartimento di Scienze Biotecnologiche di Base, Cliniche Intensivologiche e Perioperatorie, Università Cattolica del Sacro Cuore, Largo F. Vito 1, Roma 00168, Italy; ⊥ Fondazione Policlinico Universitario A. Gemelli IRCCS, Largo F. Vito 1, Roma 00168, Italy; # Department of Life, Health and Environmental Sciences, University of L’Aquila, Pza S. Tommasi 1, L’Aquila 67100, Italy; ¶ Department of Health Sciences, Section of Clinical Pharmacology and Oncology, University of Florence, Viale Pieraccini, 6, Florence 50139, Italy; ∇ Department of Pharmaceutical and Pharmacological Sciences, University of Padova, Largo Meneghetti 2, Padova 35131, Italy

## Abstract

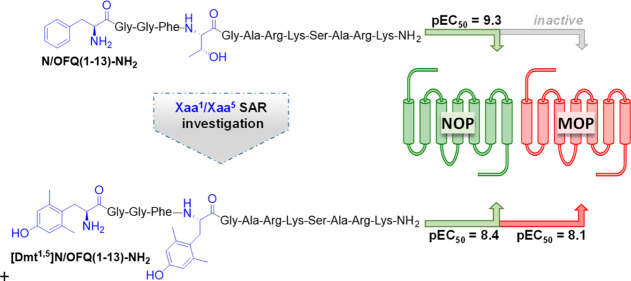

The nociceptin/orphanin FQ (N/OFQ)/N/OFQ receptor (NOP) system controls different biological functions including pain and cough reflex. Mixed NOP/opioid receptor agonists elicit similar effects to strong opioids but with reduced side effects. In this work, 31 peptides with the general sequence [Tyr/Dmt^1^,Xaa^5^]N/OFQ(1-13)-NH_2_ were synthesized and pharmacologically characterized for their action at human recombinant NOP/opioid receptors. The best results in terms of NOP versus mu opioid receptor potency were obtained by substituting both Tyr^1^ and Thr^5^ at the N-terminal portion of N/OFQ(1-13)-NH_2_ with the noncanonical amino acid Dmt. [Dmt^1,5^]N/OFQ(1-13)-NH_2_ has been identified as the most potent dual NOP/mu receptor peptide agonist so far described. Experimental data have been complemented by *in silico* studies to shed light on the molecular mechanisms by which the peptide binds the active form of the mu receptor. Finally, the compound exerted antitussive effects in an *in vivo* model of cough.

## Introduction

Nociceptin/orphanin FQ (N/OFQ; FGGFTGARKSARKLANQ) is the endogenous ligand of the N/OFQ peptide (NOP) receptor.^1,2^ N/OFQ and the NOP receptor display high structural homology with peptides and receptors of the opioid family but distinct pharmacology.^3^ The N/OFQ-NOP receptor system controls several biological functions at both central and peripheral levels including pain transmission, mood and anxiety, food intake, learning and memory, locomotion, cough and micturition reflexes, cardiovascular homeostasis, intestinal motility, and immune responses.^4^

The effects of N/OFQ and selective NOP agonists in analgesiometric assays are complex depending on the dose, administration route, type of pain, and animal species.^5,6^ On the contrary, strong and consistent experimental evidence suggests that the simultaneous activation of NOP and opioid receptors elicits synergistic analgesic effects.^6,7^ On these bases, mixed NOP/opioid receptor agonists (cebranopadol,^8,9^ AT-121,^10^ BU10038,^11^ and BPR1M97^12^) have been developed and investigated for their antinociceptive properties. It was consistently demonstrated that these drugs elicit similar analgesic effects to strong opioids but with substantially reduced side effects including respiratory depression, tolerance, and abuse liability (see the recent review by Kiguchi *et al.*(13)).

Other ligands targeting multiple opioid receptors have been studied.^14^ For example, dual-acting mu agonist/delta antagonist peptidomimetics demonstrated to produce antinociception *in vivo* with reduced tolerance liability compared with morphine.^15,16^ Moreover, mixed kappa agonist/mu partial agonist ligands have been investigated as potential treatment agents for cocaine and other psychostimulant abuses.^17^ Finally, mixed kappa agonist/delta antagonist ligands have been developed as tools for the characterization of delta and kappa-opioid receptor phenotypes.^18^

**Figure 1:**
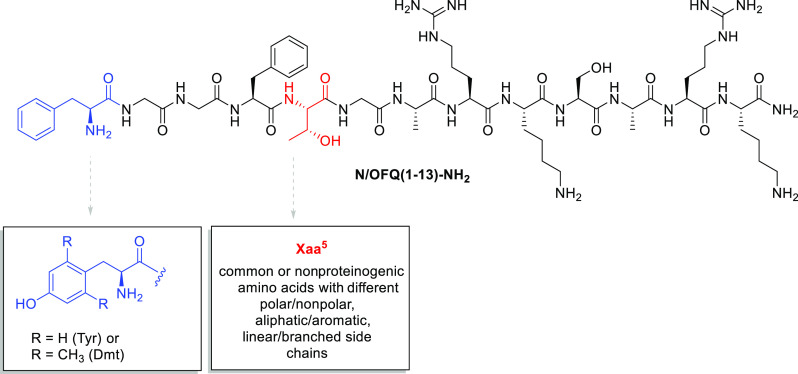
SAR investigation leading to a series of [Tyr/Dmt^1^,Xaa^5^]N/OFQ(1-13)-NH_2_ peptide derivatives as possible mixed NOP/opioid receptor ligands.

With the aim of generating a peptide acting as a nonselective NOP/opioid agonist, we investigated different approaches. On one hand, the peptide [Dmt^1^]N/OFQ(1-13)-NH_2_ has been identified as a nonselective agonist for NOP and opioid receptors^19^ and its tetrabranched derivative, generated using the peptide welding technology (PWT),^20^ was demonstrated to produce a robust analgesic effect after spinal administration in nonhuman primates. However, this action was sensitive to NOP but not opioid receptor antagonists.^21^ On the other hand, N/OFQ and dermorphin-related peptides were linked together to generate the hetero-tetrabranched derivative H-PWT1-N/OFQ-[Dmt^1^]dermorphin^22^ or the dimeric compound DeNo.^23^ Despite its promising *in vitro* pharmacological profile as a mixed NOP/opioid agonist, DeNo was not effective as a spinal analgesic.^23^ In the present study, we further investigate the possibility of generating a mixed NOP/opioid agonist based on the following evidence: (i) mixed NOP/kappa ligands can be obtained combining the C-terminal sequence of N/OFQ with the N-terminal of dynorphin A, where amino acids in positions 5 and 6 were particularly important for receptor selectivity;^24^ (ii) Thr^5^ in N/OFQ(1-13)-NH_2_ can be replaced with several different residues without loss of peptide efficacy and potency at the NOP receptor;^25^ (iii) the substitution of Phe^1^ in N/OFQ with Tyr^26^ and particularly with Dmt^19,27,28^ increases affinity/potency at classical opioid receptors. Thus, in the present study, 31 peptide derivatives with the general sequence [Tyr/Dmt^1^,Xaa^5^]N/OFQ(1-13)-NH_2_ were generated and tested for their action at NOP and opioid receptors (Figure 1).

Experimental data have been complemented by an *in silico* study of the binding of [Dmt^1,5^]N/OFQ(1-9)-NH_2_ to the mu receptor. This non-natural peptide has been compared with the agonist peptide DAMGO ([D-Ala^2^, *N*-MePhe^4^, Gly-ol]-enkephalin) and the N-terminal fragment of N/OFQ (N/OFQ(1-9)-NH_2_). The starting point of the computational study was the structure of the activated mu receptor in complex with the agonist peptide DAMGO that has been previously reported by X-ray diffraction and cryo-electron microscopy.^29,30^ The last structure of the complex DAMGO-mu receptor was used as a model, allowing the setup of the two unknown complexes with the selected peptides by molecular docking. Specifically, docking of a flexible ligand to multiple receptor conformations as already applied to the study of NOP agonists and antagonists^31,32^ was carried out to provide the best binding pose of the two peptides [Dmt^1,5^]N/OFQ(1-9)-NH_2_ and N/OFQ(1-9)-NH_2_. This docking procedure was further challenged by long-lasting molecular dynamics (MD) simulations and compared with an MD simulation of the DAMGO-mu receptor-G_i_ protein complex to identify the key interactions necessary for a successful nonselective NOP/opioid agonist. Finally, considering that NOP receptor agonists have demonstrated antitussive effects *in vivo*(33−37) and that opioids are effective drugs currently in use to treat cough,^38^ the most potent mixed NOP/opioid agonist has been evaluated *in vivo* for its antitussive effects in guinea pigs.

## Results

### Chemistry

The peptide derivatives reported in Tables 1–4 were prepared through automated Fmoc/*t*Bu-based solid-phase peptide synthesis (SPPS) on a Rink amide MBHA resin. Commercially available protected amino acids were employed as synthetic precursors of the target peptides except for Fmoc-2′,6′-dimethyl-tyrosine (Fmoc-Dmt-OH) that was instead synthesized in analogy to an approach previously published by Wang *et al.*(39) (Scheme S1 of the Supporting Information). Specifically, H-Tyr-OH was first esterified to H-Tyr-OMe under standard conditions, and then, the phenolic hydroxyl was protected with a *tert*-butyldimethylsilyl ether moiety before the following coupling with picolinic acid. The latter function worked as a directing group for the subsequent Pd(OAc)_2_-catalyzed C–H alkylation with CH_3_I and K_2_CO_3_ allowing the simultaneous and regioselective introduction of two methyl groups at the ortho-positions of the aromatic ring. Then, full deprotection under strongly acidic conditions, followed by treatment with Fmoc-Cl, led to the desired 2′,6′-dimethyl tyrosine scaffold (detailed procedures and analytical characterizations of Fmoc-Dmt-OH and its precursors have been reported in the Supporting Information). The structures of other nonproteinogenic amino acids employed in this work have been depicted in Table S1.

**Table 1:** Effects of Standard Agonists and a First Series of [Tyr^1^,Xaa^5^]N/OFQ(1-13)-NH_2_ Derivatives at NOP and mu Opioid Receptors in Calcium Mobilization Studies

		NOP		mu		NOP/mu
		pEC_50_ (CL_95%_)	*E*_max_ ± S.E.M.	pEC_50_ (CL_95%_)	*E*_max_ ± S.E.M	CR
**1**	N/OFQ(1-13)NH_2_	9.29 (9.12–9.46)	368 ± 11	inactive		<0.001
**2**	dermorphin	inactive		7.71 (7.35–8.07)	380 ± 15	>50
**3**	[Tyr^1^]N/OFQ(1-13)-NH_2_	9.23 (9.07–9.39)	380 ± 11	crc incomplete; 10 μM: 197 ± 69		0.001
**4**	[Tyr^1^,Asn^5^]N/OFQ(1-13)-NH_2_	8.70 (8.26–9.11)	327 ± 15	crc incomplete; 10 μM: 209 ± 29		0.002
**5**	[Tyr^1^,Val^5^]N/OFQ(1-13)-NH_2_	8.70 (8.53–8.87)	334 ± 19	crc incomplete; 10 μM: 254 ± 19		0.002
**6**	[Tyr^1^,Lys(Ac)^5^]N/OFQ(1-13)-NH_2_	8.55 (7.88–9.22)	370 ± 16	crc incomplete; 10 μM: 161 ± 31		0.003
**7**	[Tyr^1^,Abu^5^]N/OFQ(1-13)-NH_2_	8.15 (7.61–8.69)	366 ± 20	crc incomplete; 10 μM: 213 ± 37		0.007
**8**	[Tyr^1^,Lys^5^]N/OFQ(1-13)-NH_2_	7.26 (6.74–7.78)	366 ± 6	crc incomplete; 10 μM: 113 ± 35		0.05
**9**	[Tyr^1^,Dap^5^]N/OFQ(1-13)-NH_2_	7.20 (6.95–7.45)	367 ± 9	crc incomplete; 10 μM: 144 ± 44		0.06
**10**	[Tyr^1^,Dab^5^]N/OFQ(1-13)-NH_2_	6.43 (6.28–6.58)	340 ± 22	crc incomplete; 10 μM: 41 ± 4		0.37
**11**	[Tyr^1^,Leu^5^]N/OFQ(1-13)-NH_2_	8.03 (7.46–8.60)	339 ± 24	6.08 (5.95–6.21)	339 ± 25	0.01
**12**	[Tyr^1^,Nle^5^]N/OFQ(1-13)-NH_2_	8.77 (8.45–9.09)	375 ± 9	6.74 (6.15–7.33)	323 ± 4	0.01
**13**	[Tyr^1^,Nva^5^]N/OFQ(1-13)-NH_2_	8.43 (7.55–9.31)	358 ± 14	6.25 (5.59–6.91)	313 ± 19	0.007
**14**	[Tyr^1,5^]N/OFQ(1-13)-NH_2_	7.07 (6.68–7.46)	332 ± 19	6.76 (6.34–7.18)	357 ± 21	0.49

**Table 2:** Effects of Standard Agonists and [Tyr^1^,Xaa^5^]N/OFQ(1-13)-NH_2_ Derivatives with Different Aromatic Residues as Xaa^5^ at NOP and mu Opioid Receptors in Calcium Mobilization Studies

		NOP		mu		NOP/mu
		pEC_50_ (CL_95%_)	*E*_max_ ± S.E.M.	pEC_50_ (CL_95%_)	*E*_max_ ± S.E.M.	CR
**1**	N/OFQ(1-13)-NH_2_	9.40 (9.19–9.61)	288 ± 15	inactive		<0.001
**2**	dermorphin	inactive		7.83 (7.56–8.11)	306 ± 23	>50
**3**	[Tyr^1^]N/OFQ(1-13)-NH_2_	9.13 (8.83–9.43)	266 ± 14	crc incomplete; 10 μM: 217 ± 29		0.001
**15**	[Tyr^1^,Phe^5^]N/OFQ(1-13)-NH_2_	7.72 (7.56–7.87)	242 ± 11	6.41 (5.85–6.97)	315 ± 32	0.05
**16**	[Tyr^1^,His^5^]N/OFQ(1-13)-NH_2_	7.39 (7.16–7.62)	249 ± 23	crc incomplete; 10 μM: 230 ± 37		0.05
**17**	[Tyr^1^,Trp^5^]N/OFQ(1-13)-NH_2_	7.27 (7.19–7.35)	288 ± 28	crc incomplete; 10 μM: 193 ± 31		0.07
**18**	[Tyr^1^,hPhe^5^]N/OFQ(1-13)-NH_2_	8.70 (8.24–9.16)	301 ± 26	crc incomplete; 10 μM: 228 ± 29		0.003
**19**	[Tyr^1^,Phg^5^]N/OFQ(1-13)-NH_2_	7.57 (7.12–8.02)	293 ± 31	6.81 (6.33–7.29)	287 ± 48	0.17
**20**	[Tyr^1^,p(OCH_3_)Phe^5^]N/OFQ(1-13)-NH_2_	7.88 (7.81–7.95)	246 ± 18	crc incomplete; 10 μM: 221 ± 49		0.02
**21**	[Tyr^1^,(pF)Phe^5^]N/OFQ(1-13)-NH_2_	7.31 (6.81–7.81)	311 ± 22	crc incomplete; 10 μM: 256 ± 55		0.06
**22**	[Tyr^1^,(pNO_2_)Phe^5^]N/OFQ(1-13)-NH_2_	7.31 (6.90–7.72)	305 ± 22	crc incomplete; 10 μM: 192 ± 64		0.06
**23**	[Tyr^1^,Dip^5^]N/OFQ(1-13)-NH_2_	6.61 (6.13–7.09)	240 ± 26	6.78 (6.28–7.27)	331 ± 32	1.48
**24**	[Tyr^1^,Bip^5^]N/OFQ(1-13)-NH_2_	6.93 (6.63–7.22)	272 ± 29	crc incomplete; 10 μM: 151 ± 66		0.15
**25**	[Tyr^1^,1Nal^5^]N/OFQ(1-13)-NH_2_	7.17 (6.82–7.52)	245 ± 16	6.08 (5.67–6.49)	319 ± 31	0.08
**26**	[Tyr^1^,2Nal^5^]N/OFQ(1-13)-NH_2_	6.82 (6.60–7.04)	266 ± 15	crc incomplete; 10 μM: 244 ± 10		0.19
**27**	[Tyr^1^,(pNH_2_)Phe^5^]N/OFQ(1-13)-NH_2_	7.52 (7.11–7.93)	274 ± 15	6.43 (5.80–7.05)	329 ± 37	0.08
**28**	[Tyr^1^,Dmt^5^]N/OFQ(1-13)-NH_2_	7.75 (7.22–8.27)	251 ± 22	6.71 (6.36–7.07)	301 ± 35	0.09

**Table 3:** Effects of Standard Agonists and [Dmt^1^,Xaa^5^]N/OFQ(1-13)-NH_2_ Derivatives at NOP and mu Opioid Receptors in Calcium Mobilization Studies

		NOP		mu		NOP/mu
		pEC_50_ (CL_95%_)	*E*_max_ ± S.E.M.	pEC_50_ (CL_95%_)	*E*_max_ ± S.E.M.	CR
**1**	N/OFQ(1-13)-NH_2_	9.59 (9.30–9.88)	289 ± 34	inactive		<0.001
**2**	dermorphin	inactive		8.15 (7.97–8.33)	359 ± 18	>100
**3**	[Tyr^1^]N/OFQ(1-13)-NH_2_	9.15 (8.48–9.82)	236 ± 13	5.80 (5.22–6.38)	251 ± 34	<0.001
**29**	[Dmt^1^]N/OFQ(1-13)-NH_2_	8.57 (8.26–8.87)	294 ± 22	7.37 (7.12–7.51)	311 ± 20	0.06
**30**	[Dmt^1^,Tyr^5^]N/OFQ(1-13)-NH_2_	6.91 (6.81–7.01)	304 ± 21	7.81 (7.47–8.15)	410 ± 25	7.94
**31**	[Dmt^1^,Phe^5^]N/OFQ(1-13)-NH_2_	7.25 (6.69–7.81)	277 ± 28	8.19 (7.71–8.66)	335 ± 22	8.71
**32**	[Dmt^1^,Phg^5^]N/OFQ(1-13)-NH_2_	6.95 (6.78–7.12)	282 ± 12	8.54 (8.13–8.96)	339 ± 28	39
**33**	[Dmt^1^,1Nal^5^]N/OFQ(1-13)-NH_2_	7.22 (6.91–7.54)	286 ± 19	7.80 (7.51–8.09)	349 ± 20	3.80
**34**	[Dmt^1^,(pNH_2_)Phe^5^]N/OFQ(1-13)-NH_2_	7.58 (7.37–7.79)	258 ± 20	7.82 (7.35–8.29)	358 ± 15	1.73
**35**	[Dmt^1,5^]N/OFQ(1-13)-NH_2_	8.39 (8.05–8.72)	270 ± 25	8.08 (7.74–8.42)	354 ± 16	0.49

**Table 4:** Effects of Standard Agonists and [Dmt^1,5^]N/OFQ(1-13)-NH_2_ at NOP and mu Opioid Receptors in DMR Studies

		NOP		mu		mu/NOP
		pEC_50_ (CL_95%_)	*E*_max_ ± S.E.M.	pEC_50_ (CL_95%_)	*E*_max_ ± S.E.M.	CR
**36**	N/OFQ	9.37 (8.96–9.79)	209 ± 14	inactive		<0.001
**2**	dermorphin	inactive		8.92 (8.74–9.10)	151 ± 16	>500
**35**	[Dmt^1,5^]N/OFQ(1-13)-NH_2_	7.71 (6.65–8.76)	223 ± 11	8.64 (8.28–9.01)	202 ± 20	8.51

### *In Vitro* Structure–Activity Relationship

N/OFQ(1-13)-NH_2_ stimulated calcium mobilization with high potency and maximal effects in cells coexpressing NOP receptors and chimeric G proteins, while being inactive in cells expressing the mu opioid receptor. On the contrary, dermorphin stimulated calcium mobilization with high potency and maximal effects in mu expressing cells, while it was inactive in NOP cells (Table 1). The substitution of Phe^1^ with Tyr as in [Tyr^1^]N/OFQ(1-13)-NH_2_ did not affect NOP potency while promoting a minor increase in mu potency. Thr^5^ in [Tyr^1^]N/OFQ(1-13)-NH_2_ was replaced with a series of both proteinogenic and nonproteinogenic amino acids with different polar/nonpolar, aliphatic/aromatic, linear/branched side chains with the aim to explore the effect of several structural parameters on the biological activity. The substitution of Thr^5^ with Asn, Val, Lys(Ac) caused a slight (<10-fold) reduction in NOP potency and no modification of mu potency. The same substitution with Abu, Lys, Dap, and Dab induced a larger loss (>10-fold) of NOP potency. The introduction in position 5 of Leu, Nle, and Nva promoted a moderate decrease in NOP potency associated with a significant increase in mu potency. A similar increase in mu potency was achieved with [Tyr^1,5^ ]N/OFQ(1-13)-NH_2_, which however displayed a larger decrease in NOP potency; thus, the NOP/mu concentration ratio for this peptide was near 1 (Table 1). None of the amino acid substitutions evaluated in Table 1 modified ligand efficacy at both NOP and mu receptors. Based on these results, aromatic residues were selected for further modifications of position 5 of [Tyr^1^]N/OFQ(1-13)-NH_2_.

As shown in Table 2, 14 compounds with an aromatic residue substituting Thr^5^ in [Tyr^1^]N/OFQ(1-13)-NH_2_ were assayed in NOP and mu receptor expressing cells. The different amino acids did not modify ligand efficacy but produced different effects on NOP and mu potency. In particular, the NOP potency of these derivatives was in the range of 8.70–6.61, while the mu potency of these compounds was <6 with the exceptions of peptides substituted with Phe, Phg, 1Nal, (pNH_2_)Phe, and Dmt (range 6.08–6.81). Then, for further investigation, we selected those sequences showing pEC_50_ values >7 for the NOP receptor and >6 for the mu receptor associated with an NOP/mu concentration ratio >0.05. These criteria were matched by [Tyr^1^]N/OFQ(1-13)-NH_2_ derivatives substituted in position 5 with Tyr, Phe, Phg, 1Nal, (pNH_2_)Phe, and Dmt.

The third series of peptides was generated by substituting Tyr^1^ with Dmt that is known to increase opioid receptor potency.^40^ In fact, as shown in Table 3, [Dmt^1^]N/OFQ(1-13)-NH_2_ displayed a moderate (10-fold) decrease in NOP potency compared to [Tyr^1^]N/OFQ(1-13)-NH_2_ associated to a more pronounced increase (approx. 40-fold) in mu potency. The substitution of Thr^5^ of [Dmt^1^]N/OFQ(1-13)-NH_2_ with the above-mentioned amino acids generated results similar to those obtained with [Tyr^1^]N/OFQ(1-13)-NH_2_, that is, a slight to moderate decrease in NOP potency associated to a large increase in mu potency. The most exciting result has been obtained with [Dmt^1,5^]N/OFQ(1-13)-NH_2_ that displayed similar and high potency at both NOP and mu receptors.

[Dmt^1,5^]N/OFQ(1-13)-NH_2_ was further evaluated in dynamic mass redistribution (DMR) experiments performed on CHO cells expressing the human NOP and mu receptors. As summarized in Table 4, N/OFQ elicited a concentration-dependent positive DMR signal in cells expressing the NOP receptor being inactive in mu expressing cells. Opposite results were obtained with dermorphin that behaves as a mu-selective agonist. [Dmt^1,5^].N/OFQ(1-13)-NH_2_ elicited a robust DMR response in both cell lines with similar maximal effects to standard agonists. [Dmt^1,5^].N/OFQ(1-13)-NH_2_ displayed nanomolar potency at both NOP and the mu receptor with a mu/NOP potency ratio of 8.51 (Table 4).

**Figure 2:**
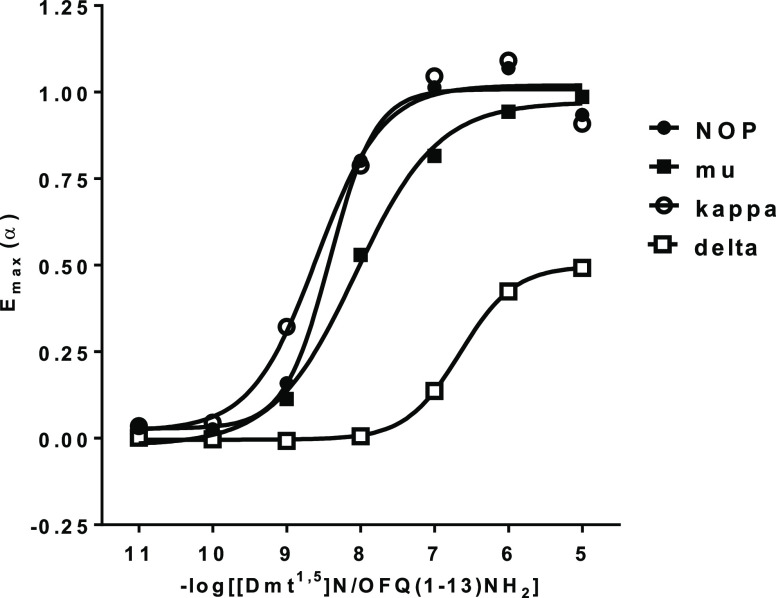
Effects of [Dmt^1,5^]N/OFQ(1-13)-NH_2_ at NOP and classical opioid receptors in calcium mobilization studies.

Finally, the agonist properties of [Dmt^1,5^]N/OFQ(1-13)-NH_2_ were evaluated at delta and kappa opioid receptors in calcium mobilization experiments. As shown in Figure 2, [Dmt^1,5^]N/OFQ(1-13)-NH_2_ displayed low potency and efficacy at the delta receptor. On the contrary, the peptide showed at the kappa opioid receptor high potency (pEC_50_ = 8.49) similar to that displayed at NOP (pEC_50_ = 8.39) and mu (pEC_50_ = 8.08) receptors. Thus, [Dmt^1,5^]N/OFQ(1-13)-NH_2_ should be classified as a mixed NOP/mu/kappa full agonist.

### Molecular Dynamics

As explained in the Experimental Section, MD simulations have been performed setting up nine-residue long peptides (i.e., [Phe/Dmt^1^,Thr/Dmt^5^]N/OFQ(1-9)-NH_2_) due to the fact that longer peptides lack reliable starting conformations by molecular docking. Moreover, in the following, we will focus on the first five residues of the peptides, those entering the mu opioid receptor orthosteric site, as residues 6–9 represent the more flexible part of the peptides along the MD simulation. The results obtained for [Dmt^1,5^]N/OFQ(1-9)-NH_2_ were compared with those obtained by similar simulations performed on the mu agonist peptide DAMGO and also on N/OFQ(1-9)-NH_2_ as a sort of negative control since this peptide lacks mu receptor affinity.^26^

**Figure 3:**
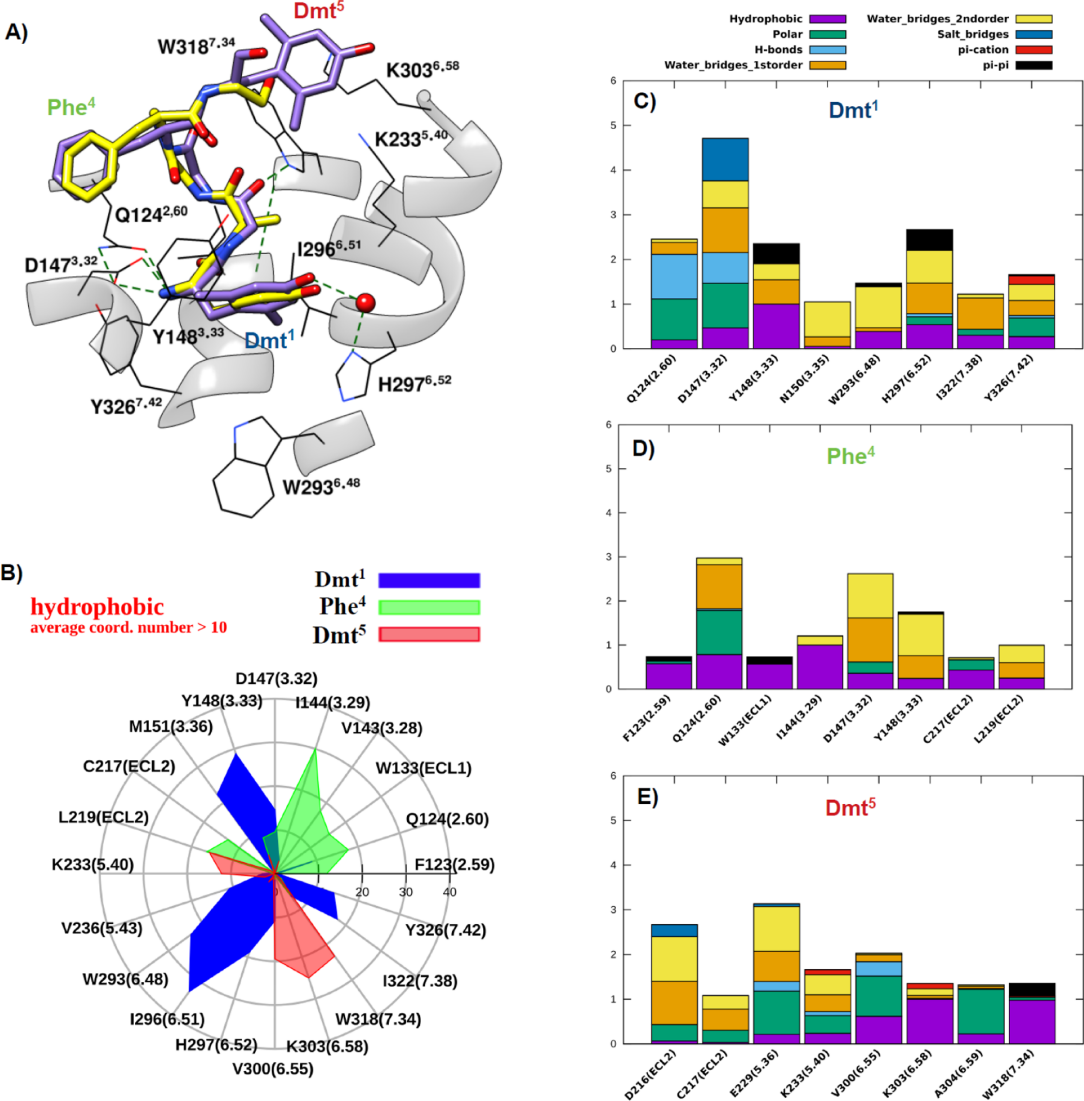
(A) Orthosteric site of [Dmt^1,5^]N/OFQ(1-9)-NH_2_ (colored purple) in the active mu receptor, according to “*in silico*” docking and MD (starting receptor structure from PDB code 6DDF). Only the first five residues are shown. The reported DAMGO conformation (the same PDB code) is superimposed (yellow). (B) Hydrophobic contacts between Dmt^1^, Phe^4^, and Dmt^5^ with their neighboring residues. (C–E) Interaction histograms of residues Dmt^1^, Phe^4^, and Dmt^5^, respectively, including hydrophobic, polar, H-bonds, water bridges of first and second order, salt bridges, and π–cation and π–π stacking as derived from long-lasting MD.

**Figure 4:**
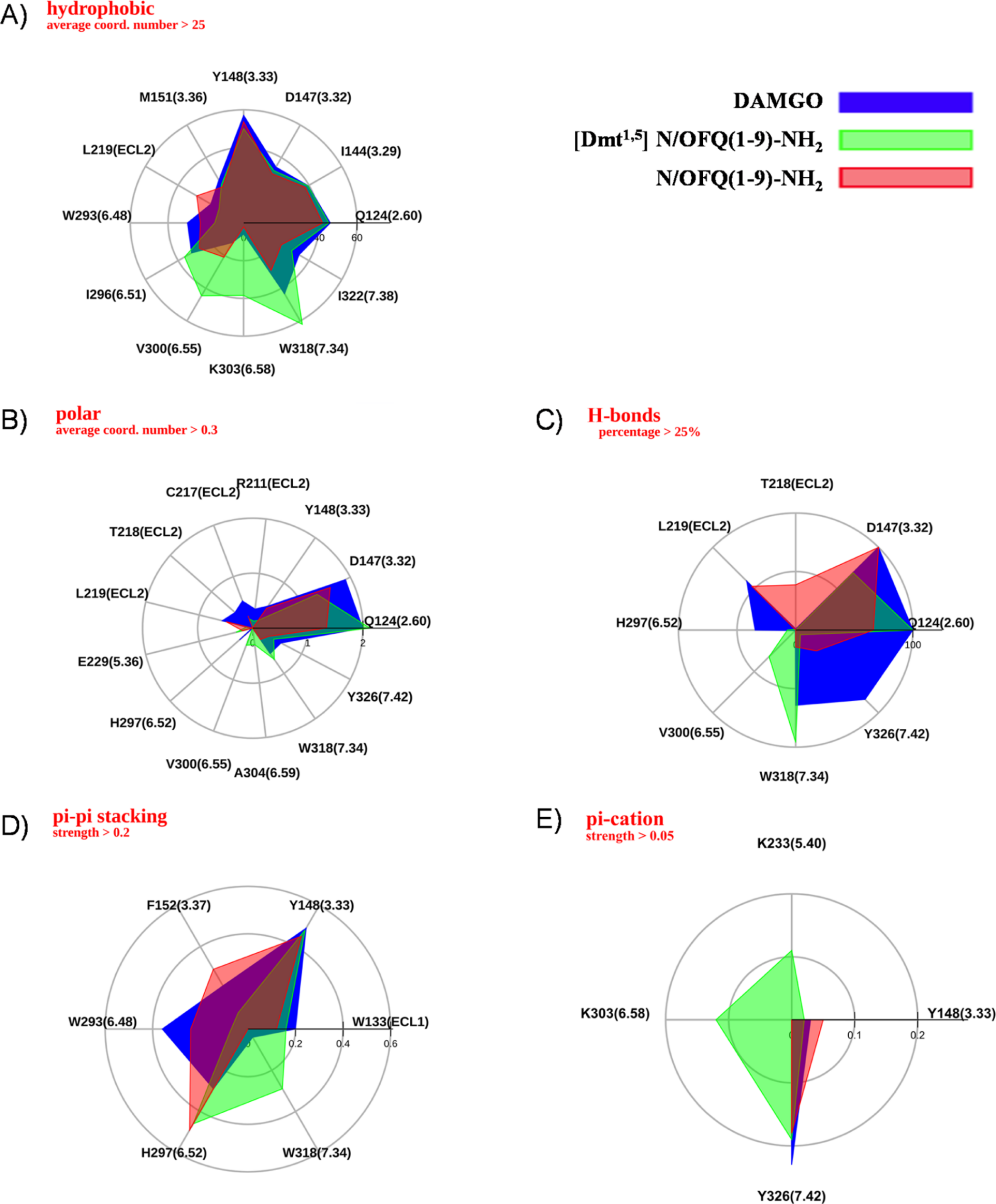
Maps of hydrophobic (A), polar (B), H-bond (C), π–π stacking (D), and π–cation (E) interactions between the mu receptor and the studied ligands along MD trajectories. Only residues 1–5 are considered for [Dmt^1,5^]N/OFQ(1-9)-NH_2_ and N/OFQ(1-9)-NH_2_.

In Figure 3A, the 3D conformation obtained after docking and MD for [Dmt^1,5^]N/OFQ(1-9)-NH_2_ (colored purple) is superimposed to the known one reported for DAMGO (colored yellow, PDB code 6DDF).^30^ Subsequent panels (Figure 3B–E) show the main interactions relating to each of the three aromatic residues, that is, Dmt^1^, Phe^4^, and Dmt^5^. Furthermore, a general comparison between the results of MD simulations performed on DAMGO (blue), [Dmt^1,5^]N/OFQ(1-9)-NH_2_ (green), and N/OFQ(1-9)-NH_2_ (red) is shown in Figure 4. In this figure, patterns of the main receptor-peptide interactions provided are displayed and superimposed, that is, hydrophobic and polar average number of contacts (Figure 4A,B), percentage of formation of hydrogen bonds, and average “strength” of π–π stacking and π–cation interaction (Figure 4C–E, respectively). Accordingly, the representative conformation of [Dmt^1,5^]N/OFQ(1-9)-NH_2_ in the orthosteric site largely overlaps with that of DAMGO (Figure 3A). The N-terminus of Dmt^1^ forms salt bridge/hydrogen bond contacts with D^147^ (a residue conserved all along the opioid family) similar to both DAMGO and the morphinan agonist BU72 (PDB code 5C1M).^29^ MD simulations show that this important interaction is strongly stabilized by the presence of another conserved residue, Q^124^ (TM2), whose nitrogen and oxygen side-chain atoms reinforce the hydrogen bond network by contacts with both the carboxyl oxygen of D^147^ and the N-terminus of Dmt^1^. Moreover, water bridges fill the small remaining volume between the D^147^ and Q^124^ side chains and the backbone donor/acceptors of Dmt^1^, Gly^3^, and Gly^2^, with the latter being in direct H-bond with W^318^ of TM7.

Dmt^1^ is also in direct hydrophobic contact with TM6 residues (W^293^, H^297^, and especially I^296^, Figure 3B,C). Along the MD trajectories, its aromatic head moves alternating first- and second-order water bridges with H^297^ of TM6 (Figure 3C). Partial π–π stacking between the Dmt^1^ and H297 rings is also observed during the simulations. Hydrophobic, π-stacking, and water bridge contacts between Dmt^1^ and Y^148^ (TM3), H^297^ and W^293^ (TM6) frequently occur (Figure 3C). The latter residue, in the so-called receptor polar cavity, is thought to be very important for the activation mechanism in many class A GPCRs, and these interactions, although not fully stable, could contribute to stabilize the receptor active state.

The formation of alternating second-order water bridges (along 78% of the trajectory) shows that the N-terminus of Dmt^1^, together with D^147^, is also in contact with N^150^ (Figure 3C), an important conserved residue that in the reported high-resolution structure of the inactive delta receptor^41^ is shown to connect the orthosteric site to the sodium pocket in the central part of the receptor.

While Dmt^1^ interacts with both TM3 (more than 40 contacts with residues Y^148^ and M^151^) and TM6 (about 80 contacts with residues W^293^, I^296^, and H^297^), Phe^4^ is immersed in the same hydrophobic pocket as the phenyl group of DAMGO between TM2 (residues F^123^ and Q^124^) and TM3 (residues V^143^ and I^144^) (Figure 3B,D), still participating with its amidic nitrogen and water bridges to the main hydrogen bond network linking the peptide to D^147^ and Q^124^ (Figure 3D).

The Dmt^5^ peptide residue mainly interacts with residues not conserved within the opiate family, that is, E^229^ and K^233^ of TM5, V^300^, and K^303^ of TM6, and W^318^ of TM7. Movements of this ring allow an alternation of nonpolar interactions with the aliphatic chains of K^303^ (TM6) and K^233^ (TM5) (Figure 3B) and of possible π–cation interactions with the positively charged amine of both the same K residues (Figure 3E). Similarly, the amidic oxygens of Gly^3^, Dmt^5^, and Gly^6^ alternate in H-bond or water bridge contacts with R^211^ (ECL2) and E^229^ (TM5) on two opposite sides of the receptor.

Details on the MD simulations of DAMGO and [Dmt^1,5^]N/OFQ(1-9)-NH_2_ in complex with the mu receptor are given in Figures S1 and S2, reporting the root-mean-square deviation (RMSD) analyses and clustering outcomes for each of the investigated peptides. Moreover, the RMSD analysis (Figure S3) and the representative conformation of residues 1–5 of N/OFQ(1-9)-NH_2_ (purple) are shown, compared to DAMGO (yellow). MD shows that the interactions of N/OFQ(1-9)-NH_2_ with TM6 are strongly diminished; in addition to the absence of polar contacts and the water density between residues 1–5 of the peptide and TM6, there are only about 20 nonpolar contacts (between Phe^1^ and W^293^ and between Phe^1^ and F^236^), while both polar and nonpolar interactions with TM3 increase.

In Figure 4, the maps of hydrophobic, polar, hydrogen bond, π–π stacking, and π–cation interactions for the peptides under study are superimposed for an overall immediate comparison. The hydrophobic and polar interaction maps are widely superimposable on all peptides (Figure 4A,B), attesting the similarity of their conformation inside the orthosteric site, with an increase of nonpolar contacts between [Dmt^1,5^]N/OFQ(1-9)-NH_2_ and TM6 (I^296^, V^300^, and K^303^) essentially due to the aromatic ring of Dmt^5^. The N-terminus of all three peptides forms hydrogen bonds with D^147^ and Q^124^ (Figure 4C). More interestingly, according to our simulation, the H-bond contact reported in the crystal structure between the amidic oxygen of Gly^3^ of DAMGO and the indole nitrogen of W^318^ is not fully stable; in the same time, the phenolic head of Tyr^1^ tends to extend toward the so-called “polar cavity” between Y^326^ and W^293^ in the intracellular side (Figure S1C), with the possibility to form π-stacking with W^293^ (Figure 4D) and a H-bond besides a π–cation contact between its N-terminus and the phenol group of Y^326^. On the other hand, the H-bond between Gly^2^ of [Dmt^1,5^]N/OFQ(1-9)-NH_2_ and W^318^ remains quite stable, as reinforced by partial π-stacking between W^318^ and Dmt^5^ (Figures 3E and 4D), while the Dmt^1^ phenolic head, sterically hindered by the two methyl groups, does not extend toward the polar cavity. Concerning N/OFQ (1-9)-NH_2_, Phe^1^ has negligible hydrogen and water bond contacts with the inner side of the receptor, and the contacts between Phe^4^, Thr^5^ of the peptide and T^218^, L^219^ of extracellular loop 2 (ECL2) are stronger (Figure 4C). The N-terminus of the three peptides can form π–cation interactions with the aromatic ring of Y^326^, while as mentioned above, π–cation contributions due to interactions of K^233^ and K^303^ are exclusive of [Dmt^1,5^]N/OFQ(1-9)-NH_2_ (Figure 4E).

### *In Vivo* Experiments: [Dmt^1,5^]N/OFQ(1-13)-NH_2_ Effect on Citric Acid-Induced Cough in the Conscious Guinea Pig

**Figure 5:**
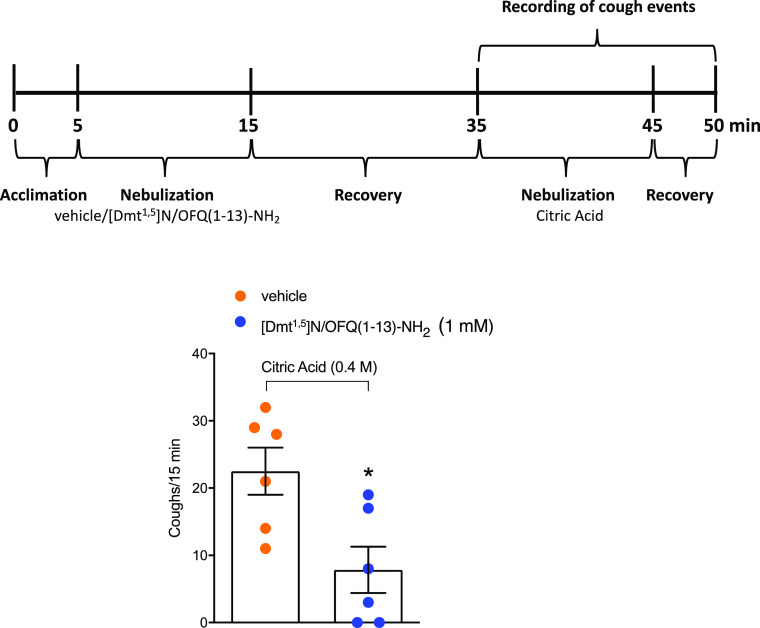
Effect of [Dmt^1,5^]N/OFQ(1-13)-NH_2_ on citric acid-induced cough in conscious guinea pigs. Schematic representation of the experimental procedure for the cough measurement in conscious guinea pigs and pooled data of cough number after [Dmt^1,5^]N/OFQ(1-13)-NH_2_ (1 mM) or vehicle (0.9% NaCl) nebulization, 30 min before the nebulization of the tussive agent, citric acid (0.4 M). Values are the mean ± SEM of the numbers of coughs/15 min, with data points overlaid (*n* = 6 guinea pigs for each condition). *p < 0.05 vs vehicle, Student’s *t*-test.

To test the antitussive effect of [Dmt^1,5^]N/OFQ(1-13)-NH_2_, we used a model of cough induced by citric acid in guinea pigs. Data showed that the coadministration of [Dmt^1,5^]N/OFQ(1-13)-NH_2_ with citric acid did not affect the tussive response (coughs/15 min: vehicle = 17.83 ± 3.95 vs [Dmt^1,5^]N/OFQ(1-13)-NH_2_ 17.67 ± 1.73). However, the nebulization with [Dmt^1,5^]N/OFQ(1-13)-NH_2_ before (30 min) the challenge with the tussive agent significantly reduced the cough number induced by citric acid (Figure 5).

## Discussion

This structure activity investigation was aimed at the identification of novel peptides acting as mixed NOP/mu receptor agonists. To this aim, we substituted Phe^1^ of N/OFQ(1-13)-NH_2_ with amino acids containing a phenol moiety and Thr^5^ with several proteinogenic and nonproteinogenic residues. Novel peptides were investigated in calcium mobilization experiments performed in cells expressing the human recombinant receptors and chimeric G proteins. The structure activity investigation led to the identification of the potent mixed agonist [Dmt^1,5^]N/OFQ(1-13)-NH_2_ whose NOP and mu agonist properties were confirmed in DMR studies. Moreover, [Dmt^1,5^]N/OFQ(1-13)-NH_2_ was also able to potently stimulate kappa but not delta opioid receptors. The capability of this peptide to bind the mu receptor has been also investigated in MD studies that suggested a similar active conformation for [Dmt^1,5^]N/OFQ(1-13)-NH_2_ and DAMGO and crucial interactions with D^147^ and H^297^. Moreover, the binding of [Dmt^1,5^]N/OFQ(1-13)-NH_2_ is reinforced by additional polar interactions of Dmt^5^ with K^223^ and K^303^. Finally, [Dmt^1,5^]N/OFQ(1-13)-NH_2_ elicited a robust antitussive action *in vivo* in a model of cough induced by nebulization of citric acid in conscious guinea pigs.

Previous studies demonstrated that the substitution of Phe^1^ in N/OFQ with Tyr reduces NOP selectivity over opioid receptors^26,42^ and that Thr^5^ of N/OFQ(1-13)-NH_2_ can be substituted with different amino acids with no changes in peptide efficacy and relatively little modifications of potency.^25^ Thus, we selected a series of amino acids to substitute Thr^5^ in [Tyr^1^]N/OFQ(1-13)-NH_2_ in order to increase the mu receptor activity of the peptide derivatives. The results obtained with [Tyr^1^]N/OFQ(1-13)-NH_2_ derivatives were similar to those previously obtained with N/OFQ(1-13)-NH_2_ derivatives in terms of NOP receptor activity. As far as the mu receptor is concerned, an increase in potency has been obtained with Leu, Nle, Nva, and Tyr. These results are not unexpected since Leu in position 5 is found in naturally occurring opioid ligands (Leu-enkephalin and dynorphin) and Nle (and possibly Nva) may mimic methionine, which is also present in position 5 of other endogenous opioid peptides (Met-enkephalin, beta-endorphin). In addition, the same can be said for Tyr^5^, which is found in amphibian opioid peptides such as the mu-selective agonist dermorphin. Moreover, previous studies demonstrated that position 5 of enkephalin can be replaced with aromatic residues^43^ or non-natural aliphatic residues^44^ with no major changes of bioactivity. Interestingly, [Tyr^1,5^]N/OFQ(1-13)-NH_2_ displayed very similar potency at NOP and the mu opioid receptor; thus, with the aim to identify potent mixed NOP/mu agonists, further studies were performed substituting position 5 with aromatic amino acids.

Despite the investigation of 14 chemically different aromatic residues, no clear structure activity information was obtained. In fact, with the exception of hPhe^5^, little changes in NOP potency were measured and the same can be said for mu receptor activity. Thus, for further studies, we selected compounds matching the following criteria: pEC_50_ > 7 for the NOP receptor and >6 for the mu receptor, with NOP/mu ratio > 0.05. This let us to select Tyr, Phe, Phg, 1Nal, (pNH_2_)Phe, and Dmt to be substituted in position 5 of [Dmt^1^]N/OFQ(1-13)-NH_2_.

The opioid receptor binding enhancing properties of Dmt in position 1^19, 27, 28^ were confirmed by the present results. In fact, compared to [Tyr^1^]N/OFQ(1-13)-NH_2_, [Dmt^1^]N/OFQ(1-13)-NH_2_ displayed approximately 3-fold reduced potency at NOP associated with almost 100-fold increased potency at the mu receptor. The same pattern of effects, that is, no change or modest reduction of NOP potency associated with a large increase in mu potency was obtained with [Dmt^1^]N/OFQ(1-13)-NH_2_ derivatives substituted in position 5 with Tyr, Phe, Phg, 1Nal, and (pNH_2_)Phe. [Dmt^1,5^]N/OFQ(1-13)-NH_2_ is however the exception to this rule; in fact, this peptide displayed, compared to [Tyr^1^,Dmt^5^]N/OFQ(1-13)-NH_2_, increased potency at both NOP and mu receptors. This led to an NOP/mu ratio of potency of [Dmt^1,5^]N/OFQ(1-13)-NH_2_ near 1. Interestingly, a very similar NOP/mu ratio was displayed by [Tyr^1,5^]N/OFQ(1-13)-NH_2_, which was however approximately 30-fold less potent at both receptors.

The calcium mobilization assay used in the present study has been previously set up^45,46^ in our laboratories and then validated by investigating a large number of NOP and opioid receptor ligands.^19,23,47^ However, this assay is based on the aberrant signaling generated by the expression of chimeric G proteins; therefore, we reassessed the pharmacological effects of [Dmt^1,5^]N/OFQ(1-13)-NH_2_ with the DMR assay. This test measures the physiological G_i_-dependent signaling of NOP and opioid receptors as demonstrated by its sensitivity to pertussis toxin treatment.^48,49^ DMR studies confirmed the mixed mu/NOP full agonist properties of [Dmt^1,5^]N/OFQ(1-13)-NH_2_.

Finally, the effects of [Dmt^1,5^]N/OFQ(1-13)-NH_2_ at kappa and delta opioid receptors were investigated. At delta receptors, [Dmt^1,5^]N/OFQ(1-13)-NH_2_ displayed low potency and efficacy, while it behaved as a potent full agonist at the kappa receptor. Of note, the kappa potency of [Dmt^1,5^]N/OFQ(1-13)-NH_2_ was similar to that shown at NOP and mu receptors. These results were not unexpected. In fact, binding experiments performed in guinea-pig brain membranes demonstrated the following rank order of affinity for [Tyr^1^]N/OFQ(1-13)-NH_2_: NOP > mu = kappa > delta.^26^ Moreover, similar results have been previously obtained in functional studies performed with human recombinant receptors with [Dmt^1^]N/OFQ(1-13)-NH_2_ that displayed the following rank order of potency: NOP = mu > kappa > delta.^19^ Collectively, these findings indicate that modifications of position 1 of N/OFQ such as Tyr and Dmt are sufficient for increasing mu and kappa but not delta receptor binding. Most probably, this is due to the fact that the C-terminal portion of N/OFQ is enriched in positively charged residues that may favor mu and kappa interactions but are detrimental for delta receptor binding.^50^

To get insights into the mechanisms by which [Dmt^1,5^]N/OFQ(1-13)-NH_2_ binds the mu receptor, MD studies were performed using the recently solved DAMGO-mu receptor-G_i_ complex.^30^ The results obtained with [Dmt^1,5^]N/OFQ(1-9)-NH_2_ were compared with those of DAMGO and N/OFQ(1-9)-NH_2_ used as the positive and negative control, respectively. These studies show that beyond the pivotal and expected interaction between the [Dmt^1,5^]N/OFQ(1-9)-NH_2_ N-terminus and D^147^, the phenol oxygen of Dmt^1^ can make first- or second-order water bridges with H^297^ (Gln in NOP) of TM6 of the mu receptor. This is in good agreement with the observation of a water bridge between the agonist BU72 and H^297^ in the active mu receptor and other small molecules or peptide mimetic agonists of kappa and delta receptors^30^ and can account for the reduction of NOP selectivity and the increase of mu potency as simply induced by the presence of the phenol groups of Tyr^1^ or Dmt^1^ in N/OFQ instead of the phenyl group of Phe^1^. Partial π–π stacking between Dmt^1^ (or Tyr^1^) and H297 could further contribute to peptide stabilization in the orthosteric site, thus enhancing these effects. Analogous contacts have been reported for cocrystallized mu^51^ and delta^52^ but not NOP^53^ antagonists. Importantly, Phe^1^ of the N/OFQ sequence cannot form water bridges with H^297^, and this is most probably the reason for the lack of mu affinity of the peptide.

Interestingly, as stated above, Dmt^5^ mainly interacts with residues that differ within the opiate family, that is, E^229^ (G in NOP, D in kappa and delta) and K^233^ (A in NOP) of TM5, V^300^ (I in kappa), and K^303^ (W, E, and Q in delta, kappa, and NOP receptor, respectively) of TM6, and W^318^ (L in NOP) of TM7. While the carbonyl oxygen of Dmt^5^ is in water bridge contact with E^229^, its aromatic bulky head is stacked between the aliphatic chains of K^303^ and K^233^, making possible π–cation interactions with the positively charged amine of both lysines (Figure 3B,E). In the reported crystal structure of mu-DAMGO^30^ (PDB code 6DDF), the K^303^ positive charge is found at a 3.3 Å distance of the carbonyl oxygen of N(Me)-Phe of DAMGO, compatible with a weak H-bond, whereas K^233^ does not appear to contribute to the stabilization of the mu active state induced by both DAMGO and BU72;^29^ the K^233^ amine group is found covalently linked to the antagonist β-funaltrexamine in the crystal structure of the inactive mu receptor.^51^ As K^303^ and K^233^ are present in the mu but not the NOP receptor, the above-mentioned interactions between Dmt^5^ and the two lysine residues could contribute to explain the mu-selective increase of affinity of [Dmt^1,5^]N/OFQ(1-13)-NH_2_ compared to [Dmt^1^]N/OFQ(1-13)-NH_2_, thus making [Dmt^1,5^]N/OFQ(1-13)-NH_2_ a mixed mu/NOP agonist. Last, as observed along the MD runs, the indole nitrogen of W^318^ in TM7 does not interact with N/OFQ but can form H-bond contact with Gly^2^ of [Dmt^1,5^]N/OFQ(1-9)-NH_2_ as well as with DAMGO (49 and 63% of the trajectory, respectively). Thus, beyond differences in steric hindrance of Tyr^1^ and Dmt^1^ that may generically contribute to a larger hydrophobic core for the last one, the entity of the interaction between W^318^ and Gly^2^ could also contribute to explain the enhanced potency of [Dmt^1,5^] N/OFQ(1-13)-NH_2_. Data obtained from this molecular modeling investigation are in agreement with those reported by recent studies performed on a series of cyclic opioid peptides.^54^

The role of the N/OFQ-NOP receptor system has been widely reported in several biological functions at the central and peripheral levels, including the cough reflex.^4^ Previous studies showed that NOP receptor agonists given centrally or peripherally suppress capsaicin and acid inhalation-induced cough in guinea pigs.^33−37^ Moreover, opioid drugs are widely used as antitussive agents,^38^ and inhalation of encephalin was shown to be effective in reducing cough reflex *in vivo*.^55^ The novel mixed NOP/opioid agonist [Dmt^1,5^]N/OFQ(1-13)-NH_2_ showed an inhibitory activity against citric acid-induced cough in guinea pigs, thus demonstrating the *in vivo* activity of the compound. However, further studies are needed to investigate the receptor mechanism involved in the antitussive action of the molecule.

## Conclusions

In this study, starting from the NOP-selective sequence of N/OFQ(1-13)-NH_2_, we developed a structure activity investigation focused on positions 1 and 5. Regarding position 1, a phenol moiety is required to increase mu receptor binding, and regarding position 5, aromatic residues generated the best results in terms of similar potency at NOP and mu receptors. This study led to the identification of [Dmt^1,5^]N/OFQ(1-13)-NH_2_ as the most potent mixed peptide agonist for NOP and mu receptors so far described in the literature. MD studies shed light on the molecular mechanisms adopted by this peptide to bind the active form of the mu receptor: some features of the mode of binding of [Dmt^1,5^]N/OFQ(1-9)-NH_2_ are superimposable to those of DAMGO, that is, the ionic bond with D^147^ of TM3 and the H-bond network with H^297^ of TM6, while others are peculiar of [Dmt^1,5^]N/OFQ(1-9)-NH_2_, that is, polar interactions of Dmt^5^ with K^223^ and K^303^ of TM5 and TM6, respectively.

[Dmt^1,5^]N/OFQ(1-13)-NH_2_ is a novel mixed agonist for NOP and mu receptors that exerted antitussive effects in an *in vivo* model of cough. The compound will be evaluated in future studies for its antinociceptive properties. In fact, mixed NOP/mu agonists of both peptide and nonpeptide structures have been consistently demonstrated in preclinical studies to promote antinociceptive effects similar to those of morphine being however better tolerated particularly in terms of respiratory depression, tolerance, and abuse liability.^13^ Importantly, phase II and III clinical studies performed with the mixed NOP/mu agonist cebranopadol have confirmed this favorable profile in pain patients.^9,56^ Nowadays, the availability of safer analgesic drugs is particularly needed for facing the opioid epidemic that leads to a progressive increase of fatal overdoses over the past 2 decades.^57^

## Experimental Section

### Chemistry

#### Materials and Methods

All solvents and reagents were purchased from Sigma-Aldrich and Fisher Scientific. Enantiopure Fmoc-protected amino acids and the resins for SPPS were purchased from AAPPTec. Peptides were synthesized using a standard Fmoc/*t*-butyl strategy^58^ with a Syro XP multiple peptide synthesizer (MultiSynTech GmbH, Witten Germany) on a Rink amide MBHA resin (4-(2′,4′-dimethoxyphenyl-Fmoc-aminomethyl)-phenoxyacetamido-norleucyl-MBHA resin; loading 0.55 mmol/g). Fmoc-amino acids were used with a 4-fold excess on a 0.11 mM scale of the resin and coupled to the growing peptide chain using *N*,*N*′-diisopropylcarbodiimide and 1-hydroxybenzotriazole (DIC/HOBt, 4-fold excess) for 1 h at room temperature. Each Fmoc removal step was performed using 40% piperidine in *N*,*N*-dimethylformamide, and all the subsequent couplings were repeated until the desired peptide-bound resin was completed. The cleavage cocktail to obtain the peptides from the resin consisted of 95% trifluoroacetic acid, 2.5% water, and 2.5% triethylsilane, and cleavages were conducted for 3 h at room temperature. After filtration of the resin, diethyl ether was added to the filtrate to promote precipitation of the peptide products that were finally isolated by centrifugation. Reverse-phase purification of crude peptides was carried out on a Waters Prep 600 high-performance liquid chromatography (HPLC) system with a Jupiter column C18 (250 × 30 mm, 300 Å, 15 μm spherical particle size) using a gradient, programmed time by time, of acetonitrile/water [with 0.1% trifluoroacetic acid (TFA)] at a flow rate of 20 mL/min. Nonpeptide derivatives were purified through flash column chromatography using a Biotage System Isolera One. Analytical HPLC was performed with a Beckman 116 liquid chromatograph furnished of a UV detector. The purity of peptides in Table 1 was assessed with a Symmetry C18 column (4.6 × 75 mm, 3.5 μm particle size, SYSTEM GOLD) at a flow rate of 0.5 mL/min using a linear gradient from 100% of A (water + 0.1% TFA) to 100% of B (acetonitrile + 0.1% TFA) over a period of 25 min. The purity of peptides in Tables 2 and 3 was assessed with an Agilent Zorbax C18 column (4.6 × 150 mm, 3.5 μm particle size, KARAT32) at a flow rate of 0.7 mL/min using a linear gradient from 100% of A (water + 0.1% TFA) to 100% of B (acetonitrile + 0.1% TFA) over a period of 25 min. All final compounds were monitored at 220 nm showing ≥95% purity, and their molecular weights were confirmed using an ESI Micromass ZQ, Waters (HPLC chromatograms and ESI mass spectra of the final peptide derivatives have been reported in the Supporting Information). ^1^H and ^13^C NMR spectra were recorded for nonpeptide derivatives on a Varian 400 MHz instrument, and all experiments were performed in deuterated DMSO using its residual shifts as reference (s: singolet, d: doublet, dd: double doublet, t: triplet, m: multiplet).

### *In Vitro* Pharmacological Studies

#### Drugs and Reagents

[D-Pen^2^,D-Pen^5^]enkephalin (DPDPE) and naltrexone were purchased from Tocris Bioscience (Bristol, UK). Concentrated solutions (1 mM) were made in bidistilled water and kept at −20 °C until use. The medium and reagents for cell culture were from Euroclone (Milan, Italy). Fluo-4 AM and pluronic acid were from Invitrogen/ThermoFisher Scientific (Waltham, USA). *N*-(2-Hydroxyethyl)piperazine-*N*′-ethanesulfonic acid (HEPES), probenecid, brilliant black, and bovine serum albumin (BSA) fraction V were from Sigma-Aldrich (St. Louis, USA).

#### Calcium Mobilization Assay

CHO cells stably coexpressing the human NOP or kappa or the mu receptor and the C-terminally modified G_αqi5_ and CHO cells coexpressing the delta receptor and the G_αqG66Di5_ protein were generated and cultured as described previously.^45,46^ Cells were maintained in Dulbecco’s modified Eagle’s medium/nutrient mixture F-12 (DEMEM/F-12) supplemented with 10% FBS, 100 U/mL penicillin and 100 μg/mL streptomycin, 100 μg/mL hygromicin B, and 200 μg/mL G418 and cultured at 37 °C in 5% CO_2_ humidified air. Cells were seeded at a density of 50,000 cells/well into 96-well black, clear-bottom plates. The following day, the cells were incubated with Hanks’ balanced salt solution (HBSS) supplemented with 2.5 mM probenecid, 3 μM of the calcium-sensitive fluorescent dye Fluo-4 AM, and 0.01% pluronic acid for 30 min at 37 °C. After that time, the loading solution was aspirated and 100 μL/well of HBSS supplemented with 20 mM HEPES, 2.5 mM probenecid, and 500 μM brilliant black was added. Serial dilutions were carried out in HBSS/HEPES (20 mM) buffer (containing 0.02% BSA fraction V). After placing both plates (cell culture and master plate) into the fluorometric imaging plate reader FlexStation II (Molecular Devices, Sunnyvale, CA), fluorescence changes were measured. On-line additions were carried out in a volume of 50 μL/well. To facilitate drug diffusion into the wells, the present studies were performed at 37 °C. Maximum change in fluorescence, expressed as percent over the baseline fluorescence, was used to determine agonist response.

#### DMR Assay

CHO cells stably expressing the human NOP and mu receptors were kindly provided by D.G. Lambert (University of Leicester, UK). Cells were cultured in DMEM/F-12 medium supplemented with 10% FBS, 100 U/mL penicillin, 100 μg/mL streptomycin, and 2 mmol/L l-glutamine. The medium was supplemented with 400 μg/mL G418 to maintain expression. Cells were cultured at 37 °C in 5% CO_2_ humidified air. For DMR measurements, the label-free EnSight Multimode Plate Reader (Perkin Elmer, MA, US) was used. Cells were seeded 15,000 cells/well in a volume of 30 μL onto fibronectin-coated 384-well DMR microplates and cultured for 20 h to obtain confluent monolayers. Cells were starved in the assay buffer (HBSS with 20 mM HEPES, 0.01% BSA fraction V) for 90 min before the test. Serial dilutions were made in the assay buffer. After reading the baseline, compounds were added in a volume of 10 μL; then, DMR changes were recorded for 60 min. Responses were described as picometer (pm) shifts over time (sec) following subtraction of values from vehicle-treated wells. Maximum picometer (pm) modification (peak) was used to generate concentration response curves. All the experiments were carried out at 37 °C.

#### Data Analysis and Terminology

The pharmacological terminology adopted in this paper is consistent with IUPHAR recommendations.^59^ All data are expressed as the mean ± standard error of the mean (SEM) of at least three experiments performed in duplicate. For potency values, 95% confidence limits (CL_95%_) were indicated. Agonist potencies are given as pEC_50_, that is, the negative logarithm to base 10 of the molar concentration of an agonist that produces 50% of the maximal effect of that agonist. Concentration-response curves to agonists were fitted to the classical four-parameter logistic nonlinear regression model:

Effect = Baseline + (*E*_max_ – Baseline)/(1 + 10^(LogEC_50_ – Log[compound]) × Hillslope^). Curve fitting was performed using PRISM 6.0 (GraphPad Software Inc., San Diego).

### Molecular Dynamics

The setup of an *in silico* model of the non-natural peptides [Dmt^1,5^] N/OFQ(1-9)-NH_2_ and N/OFQ(1-9)-NH_2_ in complex with the human mu receptor has been described in the Supporting Information. Classical MD simulations of these two receptor-peptide complexes were performed and compared with an MD simulation of the experimental system DAMGO-mu receptor-G_i_ protein complex as derived by the PDB file 6DDF.^30^ The GROMACS 2018.3 package^60^ was used under the AMBER parm99sb force field^61^ at the full atomistic level using a TIP3P water solvent and an explicit pre-equilibrated phospholipid bilayer of 128 POPC (1-palmitoyl-2-oleoyl-*sn*-glycero-3-phosphocholine) molecules obtained by the Prof. Tieleman website (http://moose.bio.ucalgary.ca). All the MD sessions were performed in a water–membrane system prepared as previously described.^31,32^ The receptor-peptide-membrane systems were solvated in a triclinic water box (having basis vector lengths of 7, 7.4, and 9.3 nm) under periodic boundary conditions for a total number of about 45,000 atoms (6400 solvent molecules). The total charge of the system was neutralized by randomly substituting water molecules with Na^+^ ions and Cl^–^ ions to obtain neutrality with a 0.15M salt concentration. Following a steepest descent minimization algorithm, the system was equilibrated under canonical ensemble (*NVT*) conditions for 300 ps using a V-rescale, modified Berendsen thermostat with position restrains for both the receptor-peptide complex and the lipids and thereafter in a isothermal–isobaric ensemble (*NPT*) for 500 ps, applying position restraints to the heavy atoms of the protein-peptide complex, and using a Nose–Hoover thermostat and a Parrinello-Rahman barostat at 1 atm with a relaxation time of 2.0 ps. The MD simulation of the mu receptor-DAMGO-G_i_ protein was carried out on the whole ternary complex without positional restraints. On the other hand, in order to reduce the computational time, in the two mu receptor-peptide complexes, the G_i_ protein was not included in the system, but all residues within 5 Å of the G_i_ protein interface were restrained to the initial structure of the activated receptor using 5.0 kcal mol^–1^ Å^–2^ harmonic restraints applied to non-hydrogen atoms. Using such restraints ensures that the receptor maintains an active conformation throughout the simulation. MD runs were performed under *NPT* conditions at 300 K with a T-coupling constant of 1 ps. van der Waals interactions were modeled using a 6–12 Lennard-Jones potential with a 1.2 nm cutoff. Long-range electrostatic interactions were calculated, with a cutoff for the real space term of 1.2 nm. All covalent bonds were constrained using the LINCS algorithm. The time step employed was 2 fs, and the coordinates were saved every 5 ps for analysis.

The MD analysis of the DAMGO-mu receptor-G_i_ protein complex (Figure S1) shows an overall stability of the starting configuration (corresponding to the crystal structure) with some motion of the phenolic head toward the intracellular side of the receptor, still conserving the water bridge contact with H^297^. A non-negligible rearrangement is observed (Figures S2 and S3) along the MD sessions, starting from the docked conformations of [Dmt^1,5^]N/OFQ(1-9)-NH_2_ and N/OFQ(1-9)-NH_2_, probably due to the limitations of the docking procedures applied to molecules with a large number of torsions, and confirms the importance of performing long-lasting MD sessions. Analysis of MD trajectories was performed using state-of-the-art computational tools, as described in the Supporting Information.

#### Artwork

3D images of peptide-receptor structures were obtained by the Chimera software.^62^

### *In Vivo* Pharmacological Studies

#### Animals

Guinea pigs (Dunkin Hartley, male, 400–450 g, Charles River, Milan, Italy) were used. The group size of *n* = 6 animals was determined by sample size estimation using G*Power (v3.1)^63^ to detect the size effect in a post-hoc test with type 1 and 2 error rates of 5 and 20%, respectively. Allocation concealment to the vehicle(s) or treatment group was performed using a randomization procedure (http://www.randomizer.org/). The assessors were blinded to the identity (allocation to the treatment group) of the animals. Guinea pigs were housed in a temperature- and humidity-controlled vivarium (12 h dark/light cycle, free access to food and water) for at least 1 week before the start of the experiments. Cough experiments were done in a quiet, temperature-controlled (20–22 °C) room between 9 am and 5 pm and were performed by an operator blinded to the treatment. All experiments were carried out according to the European Union (EU) guidelines for animal care procedures and the Italian legislation (DLgs 26/2014) application of the EU Directive 2010/63/EU. All animal studies were approved by the Animal Ethics Committee of the University of Florence and the Italian Ministry of Health (permit #450/2019-PR) and followed the animal research reporting *in vivo* experiment (ARRIVE) guidelines.

#### Measurement of Cough in Conscious Guinea Pigs

Cough experiments were performed using a whole-body plethysmography system (Buxco, Wilmington, NC, USA, upgraded version 2018).^64^ The apparatus consists of four plethysmographs (four transparent Perspex chambers) ventilated with a constant airflow and each provided by a nebulizing head (Aerogen) and adjustable bias flow rates for acclimation and nebulization. The particle size presents an aerodynamic mass median diameter of 6 μm, and the output of the nebulizing heads can be set in the range between 0 and 0.4 mL per minute. The number of elicited coughs was automatically counted using the instrument. The nebulization rate used in the following experiments was 0.15 mL/min, and the air flows were 1750 mL/min during the acclimation phase and 800 mL/min during nebulization. These rates were previously found in our lab to elicit a significant number of cough events in the citric acid-induced cough model.

On the day of experiments, guinea pigs were individually placed into the chambers and let to acclimate for 10 min. To test the antitussive effect of [Dmt^1,5^]N/OFQ(1-13)-NH_2_, two different protocols were used. Protocol 1: after acclimation, a mixture of [Dmt^1,5^]N/OFQ(1-13)-NH_2_ (1 mM) or its vehicle (0.9% NaCl) and the tussive agent, citric acid (0.4 M), was nebulized for 10 min. During the 10 min of nebulization and for 5 min immediately post challenge (recovery period), the number of elicited coughs was automatically recorded using the BUXCO system. Protocol 2: after acclimation, [Dmt^1,5^]N/OFQ(1-13)-NH_2_ (1 mM) or its vehicle (0.9% NaCl) was nebulized for 10 min. After 20 min of recovery, the tussive agent, citric acid (0.4 M), was delivered by aerosol via a nebulizer for 10 min. During the 10 min of the citric acid challenge and 5 min immediately post challenge (recovery period), the number of elicited coughs was automatically recorded using the BUXCO system.

For the *in vivo* experiment, the statistical significance of differences between groups was assessed using Student’s *t*-test.

## Supporting Information Available

Synthesis of Fmoc-2′,6′-dimethyltyrosine; synthetic procedures for the preparation of Fmoc-2′,6′-dimethyltyrosine; structures of nonproteinogenic amino acids; HPLC chromatograms and ESI mass spectra of the final peptide derivatives; model setup of non-natural peptides for MD; model setup of the mu receptor; model setup of the peptide-mu receptor complexes; and analysis of MD trajectories (PDF)
Molecular formula strings (CSV)
Model coordinates: representative structure of the [Dmt^1,5^]N/OFQ(1-9)-NH_2_-mu receptor complex (PDB)
Model coordinates: representative structure of the N/OFQ(1-9)NH_2_-mu receptor complex (PDB)

## Supplementary Material

jm0c02062_si_002.csv

jm0c02062_si_003.pdb

jm0c02062_si_004.pdb

## Abbreviations

CHO cells: chinese hamster ovary cells
CL_95%_: 95% confidence limits
CR: concentration ratio
crc: concentration–response curve
DAMGO: [D-Ala^2^, *N*-MePhe^4^, Gly-ol]-enkephalin
DIC: *N*,*N*′-diisopropylcarbodiimide
DIPEA: *N*,*N*-diisopropylethylamine
DMEM/F-12: Dulbecco’s modified Eagle’s medium/nutrient mixture F-12
DMR: dynamic mass redistribution
DPDPE: [D-Pen^2^,D-Pen^5^]enkephalin
EtOAc: ethyl acetate
FBS: fetal bovine serum
FmocCl: Fmoc chloride
HATU: hexafluorophosphate azabenzotriazole tetramethyl uronium
HBSS: Hanks’ balanced salt solution
LINCS: LINear Constraint Solver
MBHA resin: 4-methylbenzhydrylamine resin
N/OFQ: nociceptin/orphanin FQ
*NPT*: isothermal–isobaric ensemble
*NVT*: canonical ensemble conditions
PWT: peptide welding technology
SEM: standard error of the mean
SPPS: solid-phase peptide synthesis
