# Supplementary material for: Novel Mixed NOP/Opioid Receptor Peptide Agonists
Source: J Med Chem. 2021 May 17;64(10):6656–69. doi: 10.1021/acs.jmedchem.0c02062 (PMC8279409; doi:10.1021/acs.jmedchem.0c02062)
Supplement: Supplementary file 1 — jm0c02062_si_001.pdf [file jm0c02062_si_001.pdf]

Novel mixed NOP/opioid receptor peptide agonists

Salvatore Pacifico[1], Valentina Albanese[1], Davide Illuminati[1], Erika Marzola[1], Martina Fabbri[1], Federica Ferrari[2], Victor A.D. Holanda[2], Chiara Sturaro[2], Davide Malfacini[3], Chiara Ruzza[2, 4*], Claudio Trapella[1,4], Delia Preti[1*], Ettore Lo Cascio[5], Alessandro Arcovito[5, 6], Stefano Della Longa[7*], Martina Marangoni[8], Davide Fattori[8], Romina Nassini[8], Girolamo Calò[3], and Remo Guerrini[1, 4].

[1]*Department of Chemical, Pharmaceutical and Agricultural Sciences, University of Ferrara, Via Luigi Borsari 46, 44121 Ferrara, Italy.*

[2]*Department of Neuroscience and Rehabilitation, Section of Pharmacology, University of Ferrara, Via Fossato di Mortara 17/19, 44121 Ferrara, Italy.*

[3]*Department of Pharmaceutical and Pharmacological Sciences, University of Padova, Largo Meneghetti 2, 35131 Padova, Italy*

[4]*Technopole of Ferrara, LTTA Laboratory for Advanced Therapies, via Fossato di Mortara 70, 44121 Ferrara, Italy.*

[5]*Dipartimento di Scienze Biotecnologiche di Base, Cliniche Intensivologiche e Perioperatorie, Università Cattolica del Sacro Cuore, Largo F. Vito 1, 00168 Roma, Italy.*

[6]*Fondazione Policlinico Universitario A. Gemelli IRCCS, Largo F. Vito 1, 00168 Roma, Italy.*

[7]*Department of Life, Health and Environmental Sciences, University of L'Aquila, Pza S. Tommasi 1, 67100 L'Aquila, Italy*

[8]*Department of Health Sciences, Section of Clinical Pharmacology and Oncology, University of Florence, Viale Pieraccini, 6, 50139, Florence, Italy*

CONTENTS

	Pag.
Scheme S1. Synthesis of Fmoc-2',6'-dimethyltyrosine	S2
Synthetic procedures for the preparation of Fmoc-2',6'-dimethyltyrosine	S3-S5
Table S1.Structures of non-proteinogenic amino acids	S6
HPLC chromatograms and ESI mass spectra of the final peptide derivatives	S7-S41
Model set-up of non-natural peptides for MD	S42
Model set-up of the mu receptor	S42
Model set-up of the peptide-mu receptor complexes	S42
Analysis of molecular dynamics trajectories	S43
Figures S1-S3	S44-S46
References	S47

CHEMISTRY

Scheme S1. Synthesis of Fmoc-2',6'-dimethyl-tyrosine developed in analogy to what previously published by Wang et *al*.[1]

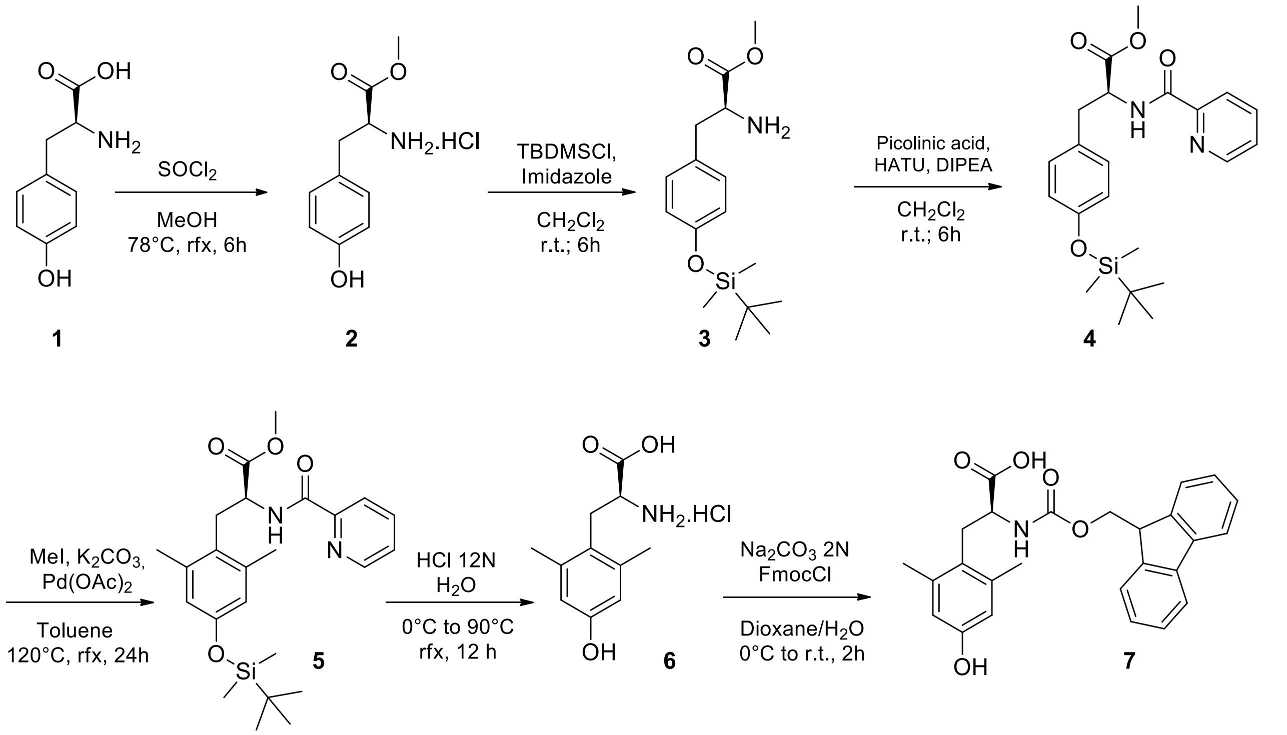

Synthesis of (*S*)-methyl 3-(4-((tert-butyldimethylsilyl)oxy)phenyl)-2-(picolinamido)propanoate (4)

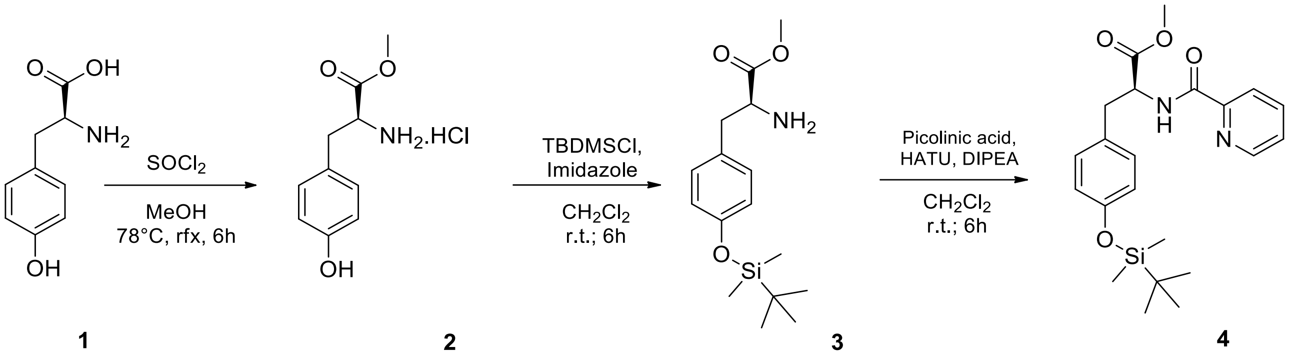

1 2 3 4

To a suspension of (S)-Tyrosine **1** (16.56 mmol) in MeOH (30 mL), $SOCl_2$ (1.1 eq; 18.21 mmol) was added dropwise at 0 °C and the resulting mixture was heated at reflux and left stirring overnight. After the removal of the solvent, the methyl ester derivative **2** was obtained as a white powder that was used in the next step without further purifications (the structure was confirmed by ESI mass analysis of the crude product). The hydrochloride salt **2** was suspended in CH_2Cl_2 (90 mL) then imidazole (1.2 eq; 19.87 mmol) and *tert*-butyldimethylsilyl chloride (1.2 eq; 19.87 mmol) were added to the mixture that was stirred at r.t. overnight. Once completed, the reaction was quenched with sat. $NaHCO_3$ (30 mL), the organic phase was isolated in a separatory funnel and the aqueous phase was further extracted with CH_2Cl_2 (2 x 20 mL). The organic layers were dried over Na_2SO_4, filtered, and concentrated under vacuum. Compound **3** was obtained as a yellowish oil which was used in the next step without further purifications. To a solution of **3** (1 eq; 16.56 mmol) in CH_2Cl_2 (90 mL), HATU (1.2 eq; 19.87 mmol), DIPEA (2.5 eq; 41.39 mmol) and Picolinic acid (1.2 eq; 19.87 mmol) were added. The reaction was stirred at r.t. overnight. The mixture was quenched with NH_4Cl (30 mL), the organic phase was isolated in a separatory funnel and the aqueous phase was further extracted with CH_2Cl_2 (2 x 20 mL). After drying over anhydrous Na_2SO_4, the solvent was removed under vacuum. The crude product was purified via flash column chromatography (AcOEt/Petroleum ether 3,5: 6,5) to obtain **4** as a white powder (Yield of 3 steps 83%).

^{1}H NMR (400 MHz, Chloroform-*d*): δ 8.58 – 8.56 (m, 1H), 8.54 (d, *J* = 8.4 Hz, 1H), 8.11 (dt, *J* = 7.8, 1.1 Hz, 1H), 7.82 (td, *J* = 7.7, 1.7 Hz, 1H), 7.44 – 7.41 (m, 1H), 6.49 (s, 2H), 4.94 (q, *J* = 8.1 Hz, 1H), 3.65 (s, 3H), 3.19 – 3.15 (m, 2H), 0.96 (s, 9H), 0.16 (s, 6H). MS (ESI): $[M+H]^+$ 415.43. T_R: 25.12 min.

Synthesis of (*S*)-methyl 3-(4-((tert-butyldimethylsilyl)oxy)-2,6-dimethylphenyl)-2-(picolinamido)propanoate (5)

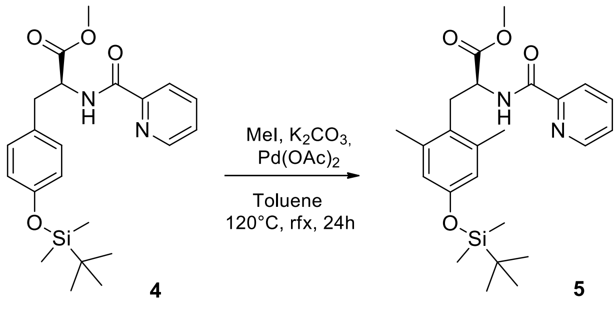

4 **5**

The picolinamido derivative **4** (1 eq; 13.74 mmol) was solubilized in toluene (60 mL) then K_2CO_3 (3 eq; 41.23 mmol), CH_3I (5 eq; 68.71 mmol) and $Pd(OAc)_2$ (0.1 eq; 1.37 mmol) were added and the mixture was left stirring at 120 °C for 24h. The crude mixture was filtered through a celite pad, the filtrate was concentrated under vacuum and purified by flash column chromatography (AcOEt/Petroleum ether 3:7) affording **5** as an orange amorphous solid (Yield: 72%).

^{1}H NMR (400 MHz, Chloroform-*d*): δ 8.58 – 8.56 (m, 1H), 8.54 (d, *J* = 8.5 Hz, 1H), 8.11 (dt, *J* = 7.8, 1.1 Hz, 1H), 7.82 (td, *J* = 7.7, 1.7 Hz, 1H), 7.42 (ddd, *J* = 7.6, 4.8, 1.2 Hz, 1H), 6.49 (s, 2H), 4.94 (q, *J* = 8.1 Hz, 1H), 3.65 (s, 3H), 3.18 – 3.16 (m, 2H), 2.35 – 2.33 (m, 6H), 0.95 (s, 9H), 0.15 (s, 6H). MS (ESI): $[M+H]^+$ 443.31 T_R: 24.12 min.

Synthesis of (*S*)-2-((((9H-fluoren-9-yl)methoxy)carbonyl)amino)-3-(4-hydroxy-2,6-dimethylphenyl)propanoic acid (7)

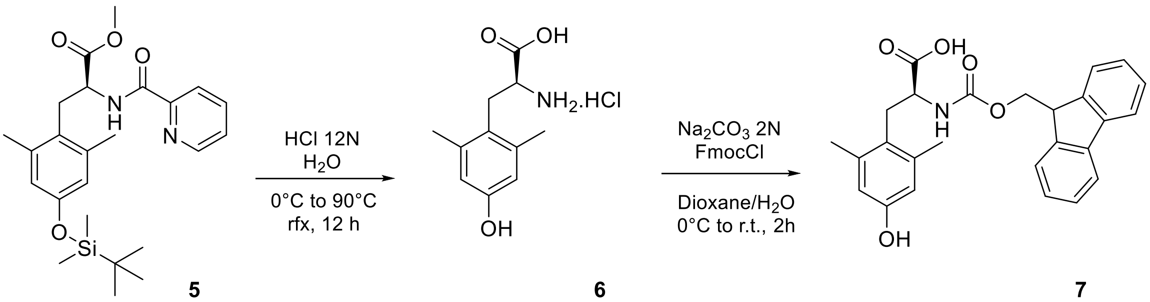

The picolinamido derivative **5** (1 eq; 9.89 mmol) was deprotected by adding 6 N HCl (28 ml; 168 mmol) and refluxing the mixture for 12h. After this time, the reaction was cooled at r.t. and concentrated under vacuum to afford a crude residue that was directly used for the next step. Compound **6** was suspended in a mixture of water/dioxane 1:2 (20 mL: 40 mL) and Na_2CO_3 (3 eq; 29.59 mmol) was added to reach pH 10. Finally, FmocCl (0.9 eq; 8,87 mmol) was added at 0°C. The reaction was left stirring at r.t. for 2h and monitored via TLC (AcOEt/Petroleum ether 7:3 + 1% Acetic acid). Once the reaction was completed, the solvents were concentrated to half volume under vacuum and 1N HCl was added at 0 °C until reaching pH 1-2. The aqueous phase was extracted with AcOEt (3 x 20 mL) and the organic layers were dried over Na_2SO_4, concentrated, and the crude residue was purified by flash chromatography (AcOEt/Petroleum ether 7:3 + 1% Acetic acid). Compound **7** was obtained as a white powder (Yield: 72%).

^{1}H NMR (400 MHz, DMSO-d_6): δ 12.63 (s, 1H), 8.95 (s, 1H), 7.89 (d, *J* = 7.6 Hz, 2H), 7.77 (d, *J* = 8.6 Hz, 1H), 7.70 – 7.67 (m, 2H), 7.41 (td, *J* = 7.6, 3.0 Hz, 2H), 7.32 (q, *J* = 7.3 Hz, 2H), 6.38 (s, 2H), 4.20 – 4.17 (m, 2H), 4.12 – 4.05 (m, 1H), 3.33 (bs, 2H), 3.03 – 2.98 (m, 1H), 2.86 – 2.80 (m, 1H), 2.20 (s, 6H). **^{13}C NMR** (DMSO-d_6): δ 173.70, 155.90, 155.10, 143.83, 143.75, 140.68, 137.77, 127.63, 127.05, 125.31, 125.11, 120.10, 114.82, 65.68, 54.29, 46.57, 30.71, 20.04. MS (ESI): $[M+H]^+$ 431.13.T_R: 19.38 min.

Table S1.Structures of non-proteinogenic amino acids

Abbreviation	Chemical Structure	Abbreviation	Chemical Structure
**Abu**	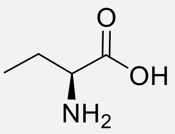	**(pNO₂)Phe**	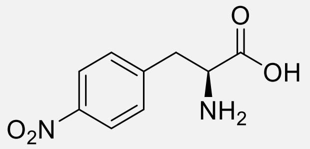
**Dap**	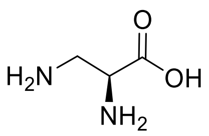	**(pNH₂)Phe**	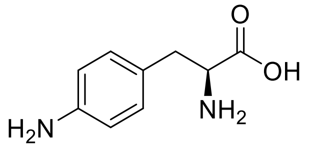
**Dab**	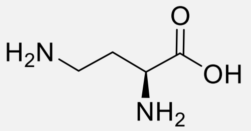	**Dip**	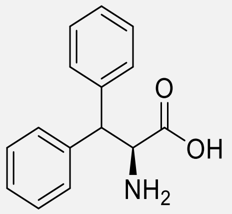
**Nva**	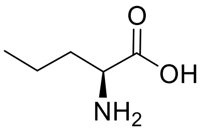	**Bip**	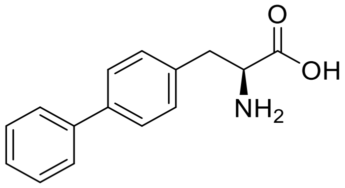
**hPhe**	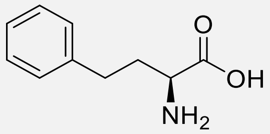	**1Nal**	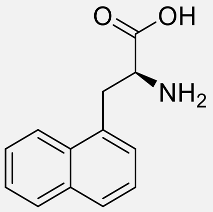
**Phg**	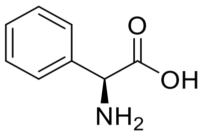	**2Nal**	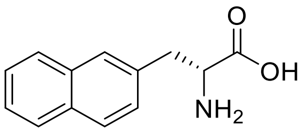
**(pOCH₃)Phe**	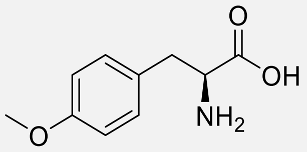	**Dmt**	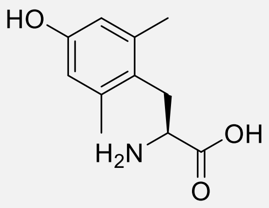
**(pF)Phe**	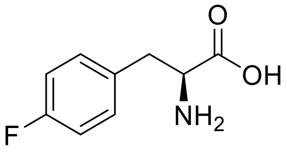		

N/OFQ(1-13)NH_2

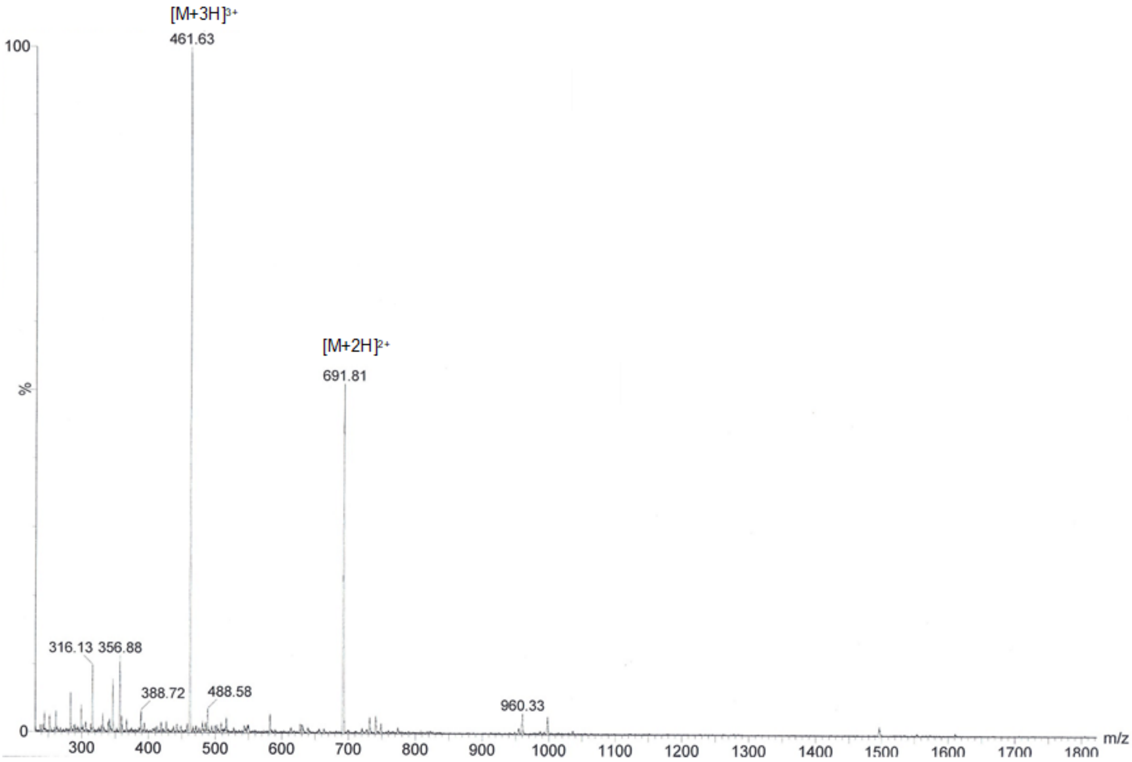
[M+3H]3+
461.63
[M+2H]2+
691.81
316.13 356.88
388.72
488.58
960.33
100
%
0
300
400
500
600
700
800
900
1000
1100
1200
1300
1400
1500
1600
1700
1800
m/z

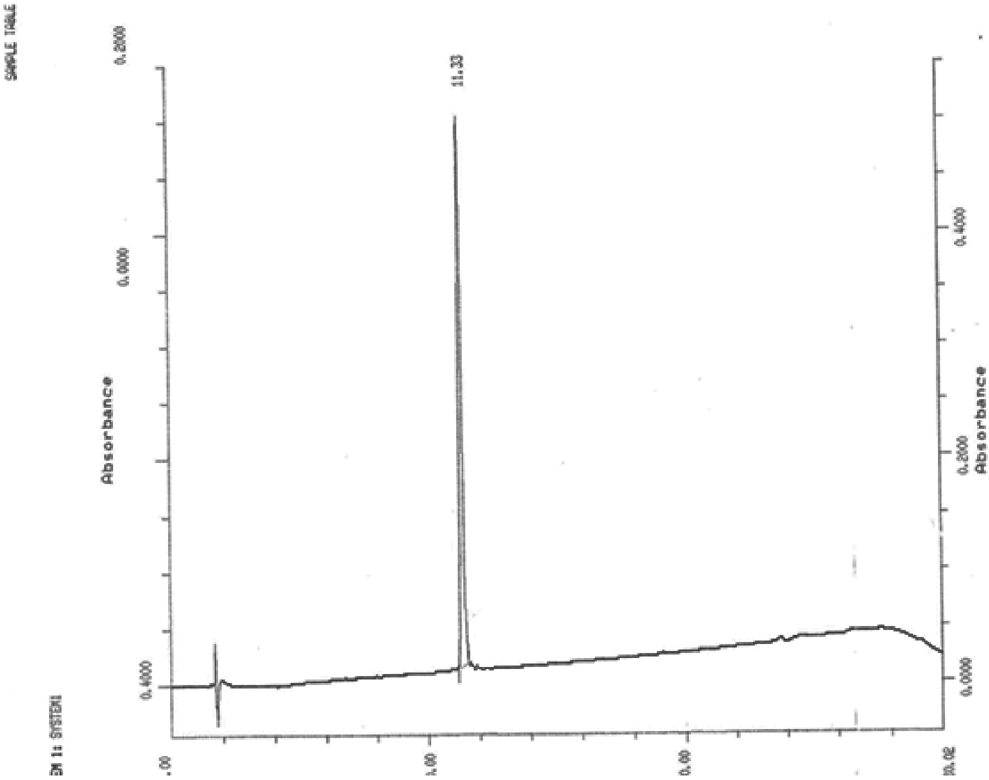
11.33
Absorbance
Absorbance

[Tyr[1]]N/OFQ(1-13)NH_2

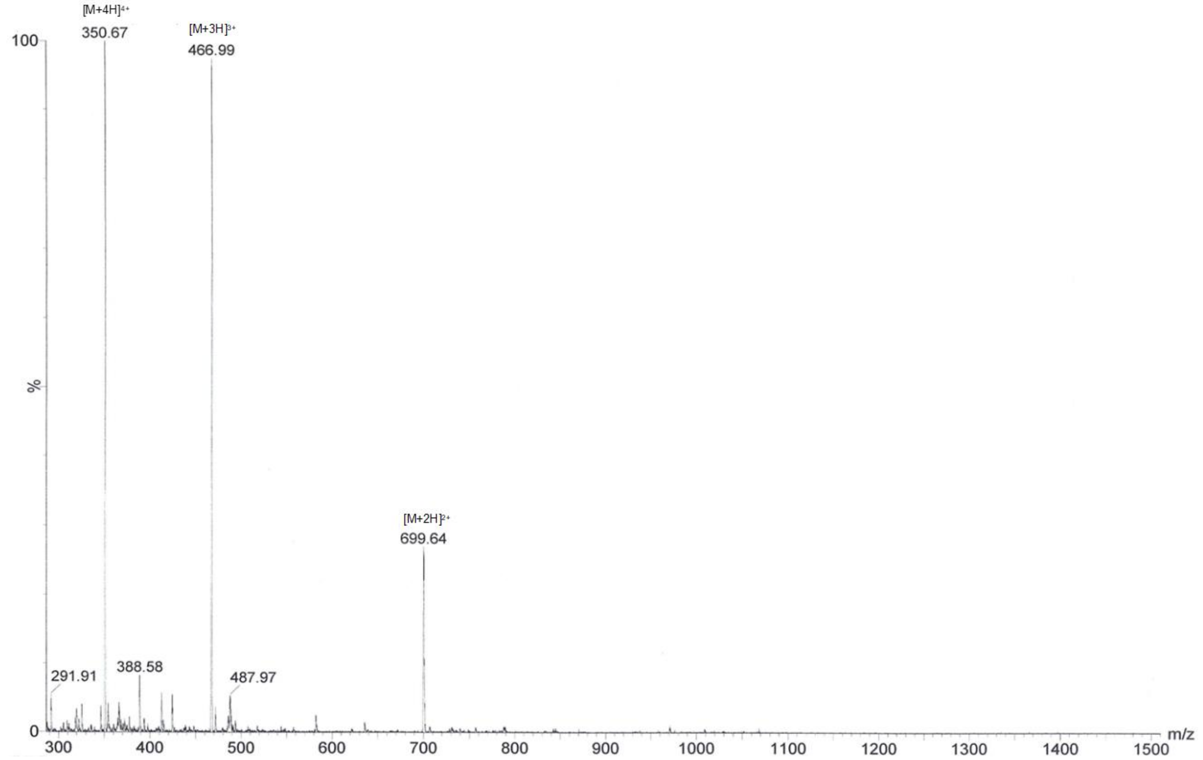

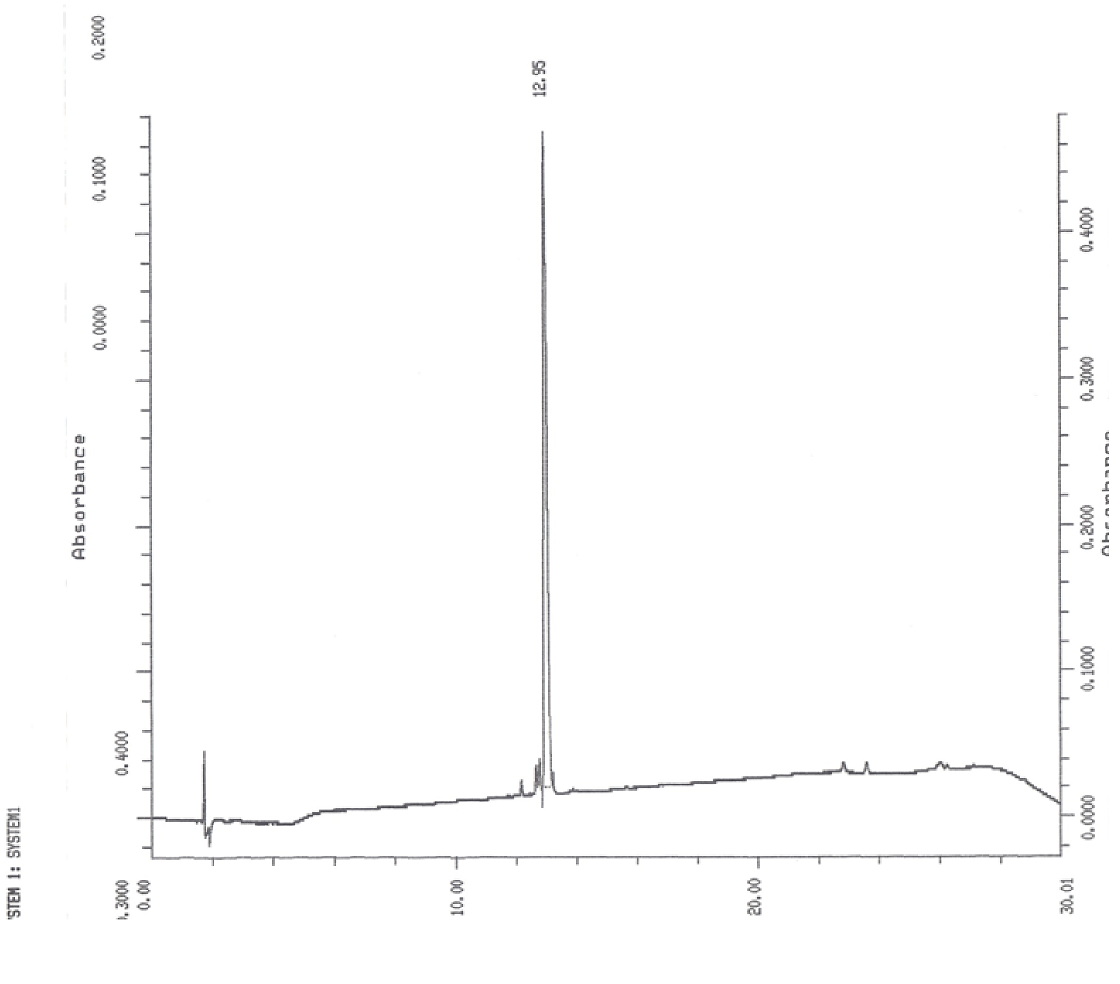

[Dmt[1]]N/OFQ(1-13)NH_2

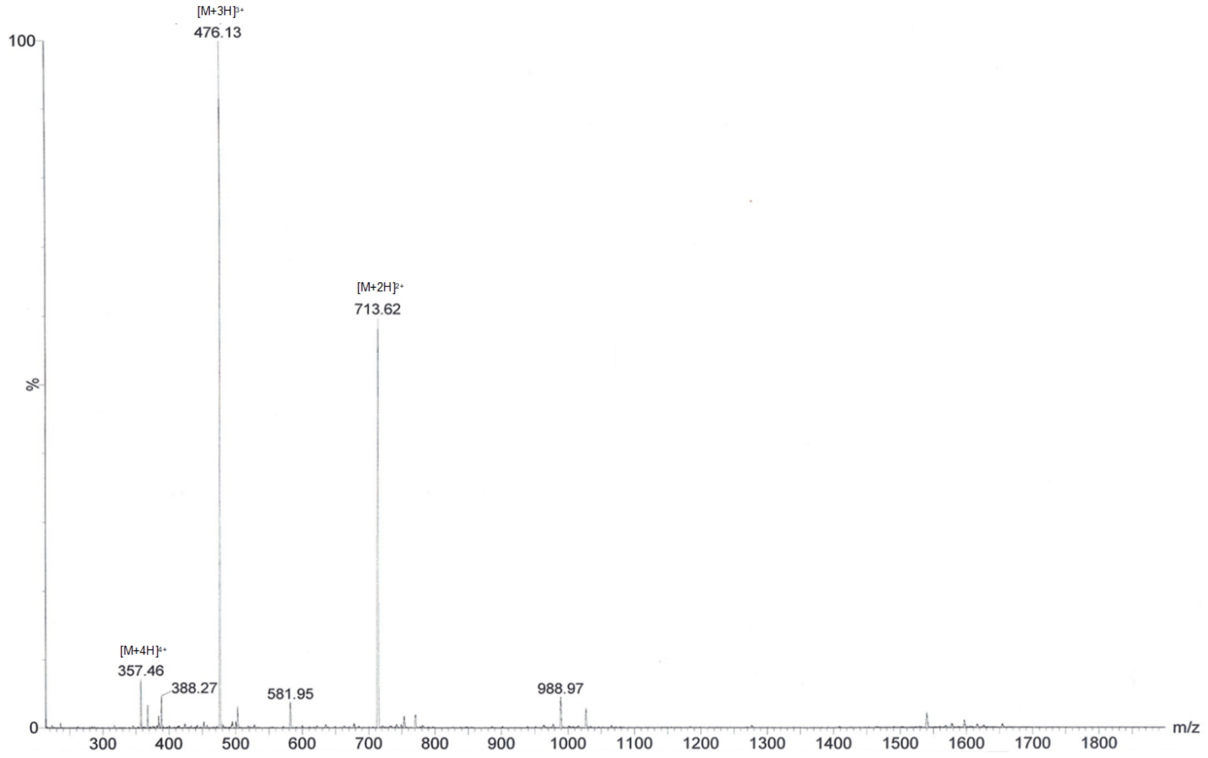

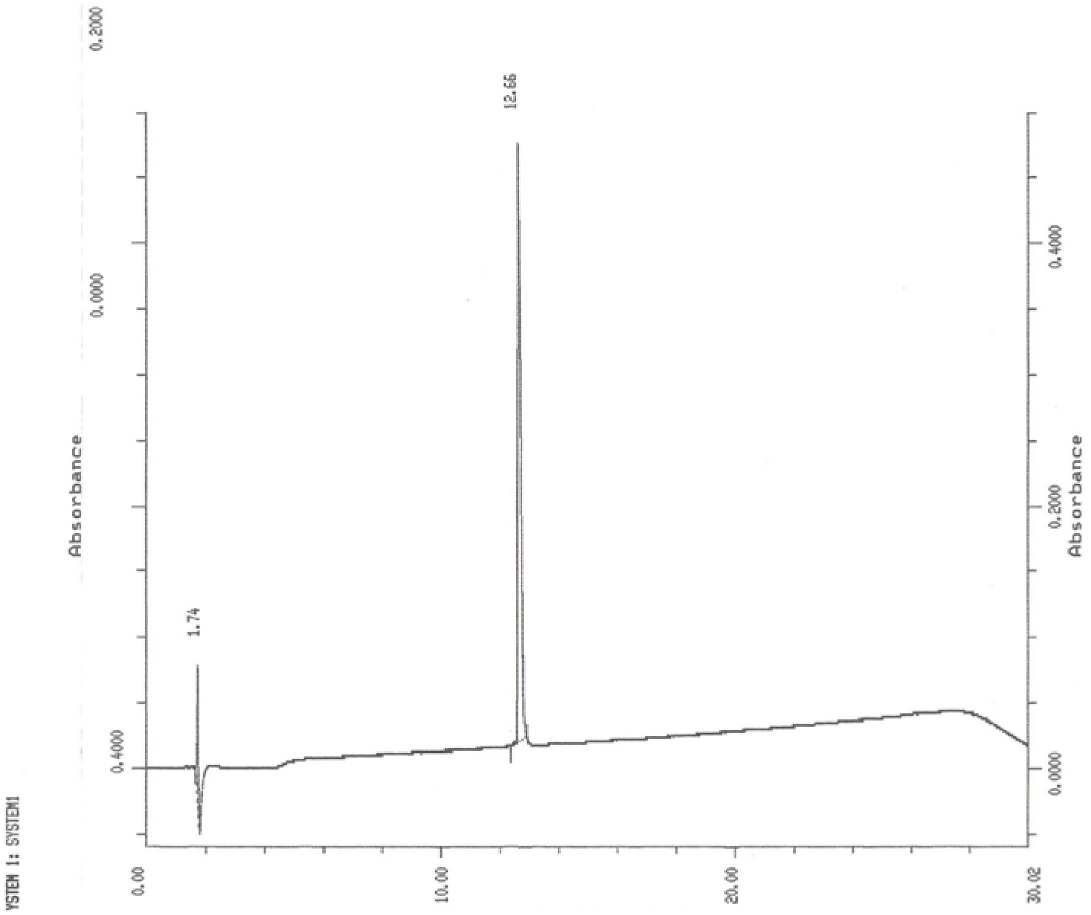

[Dmt[1],Tyr[5]]N/OFQ(1-13)NH_2

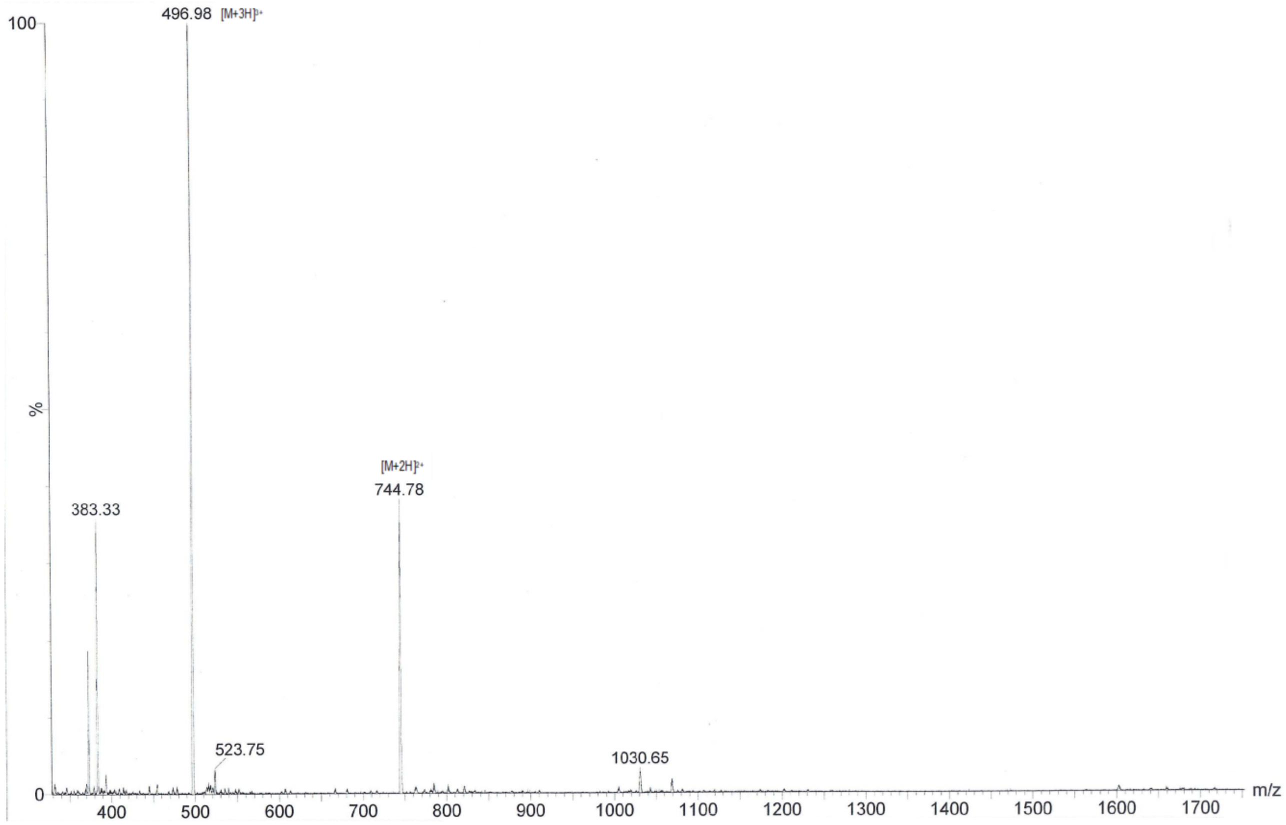

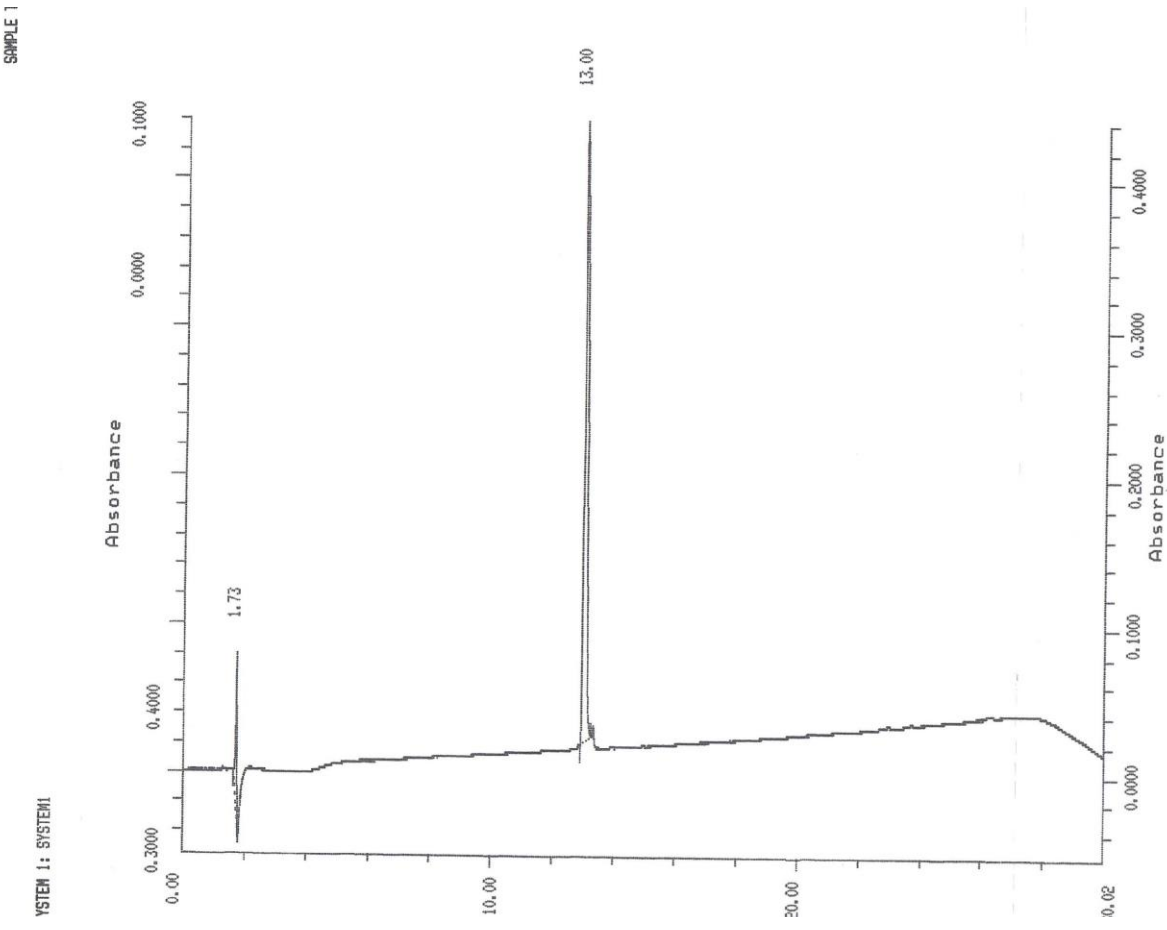

Dermorphin

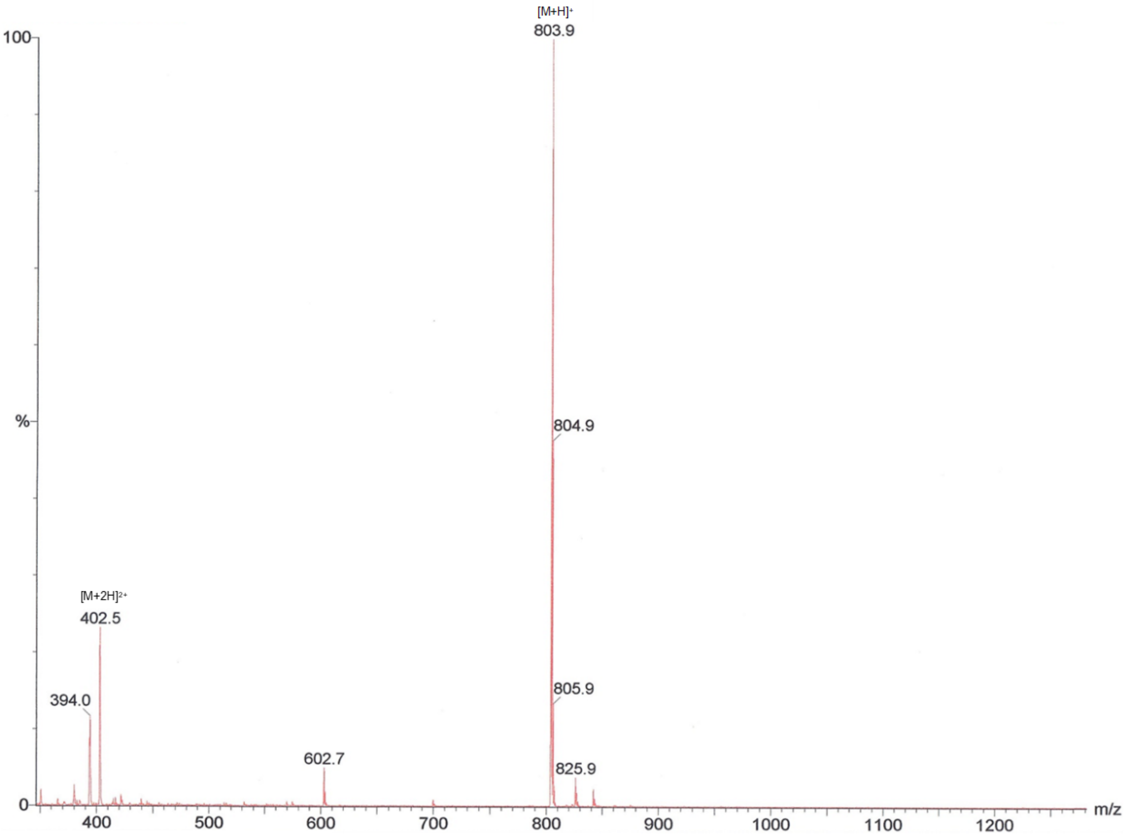

[M+H]+
803.9
100
%
0
804.9
[M+2H]2+
402.5
394.0
805.9
602.7
825.9
400
500
600
700
800
900
1000
1100
1200
m/z

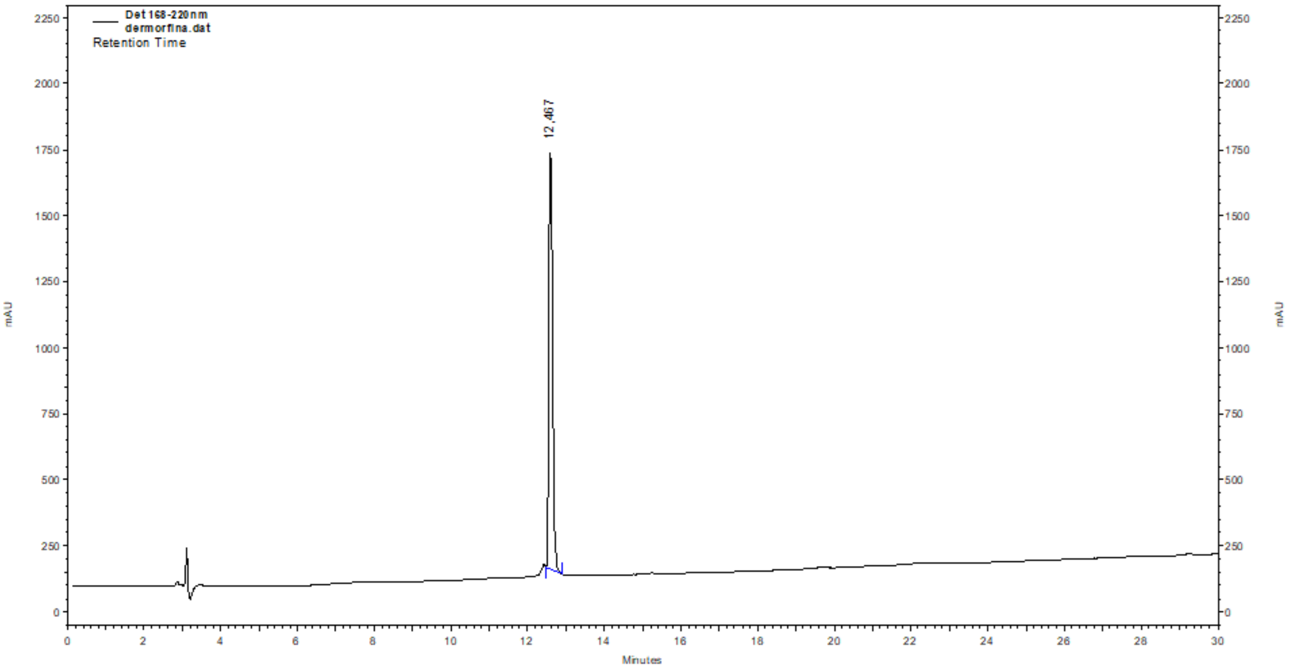

Det 168-220nm
dermorfina.dat
Retention Time
12.467
mAU
Minutes

[Tyr[1]Asn[5]]N/OFQ(1-13)NH$_2$

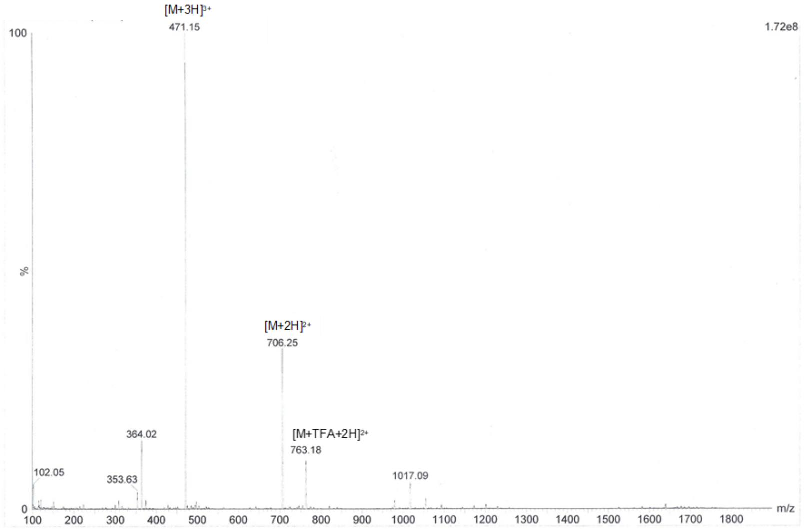

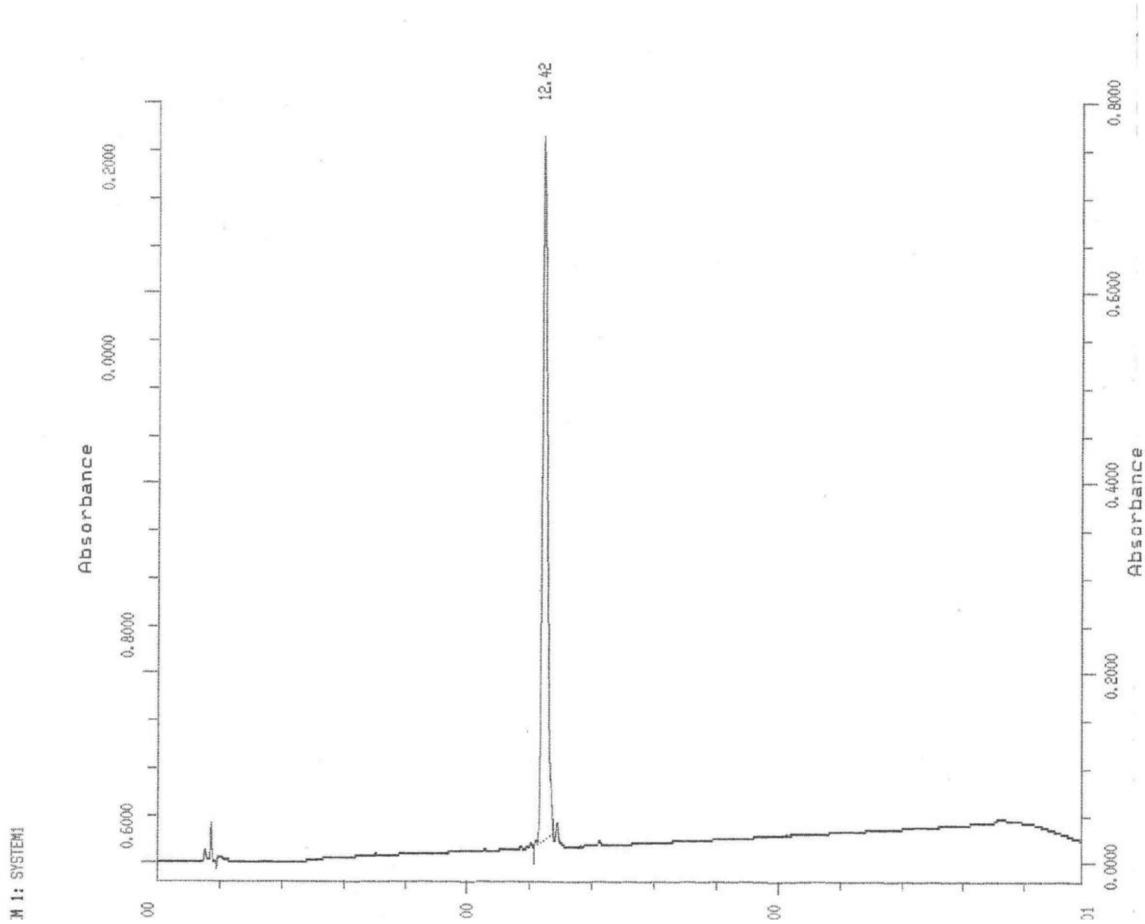

[Tyr[1]Val[5]]N/OFQ(1-13)NH_2

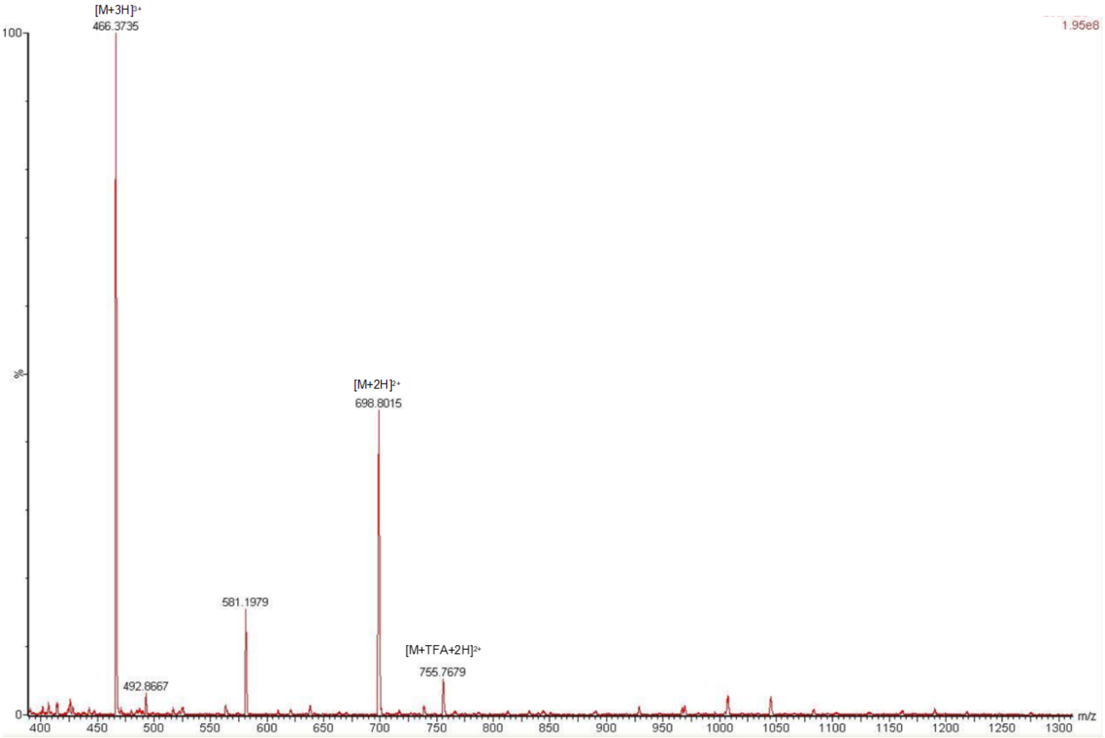

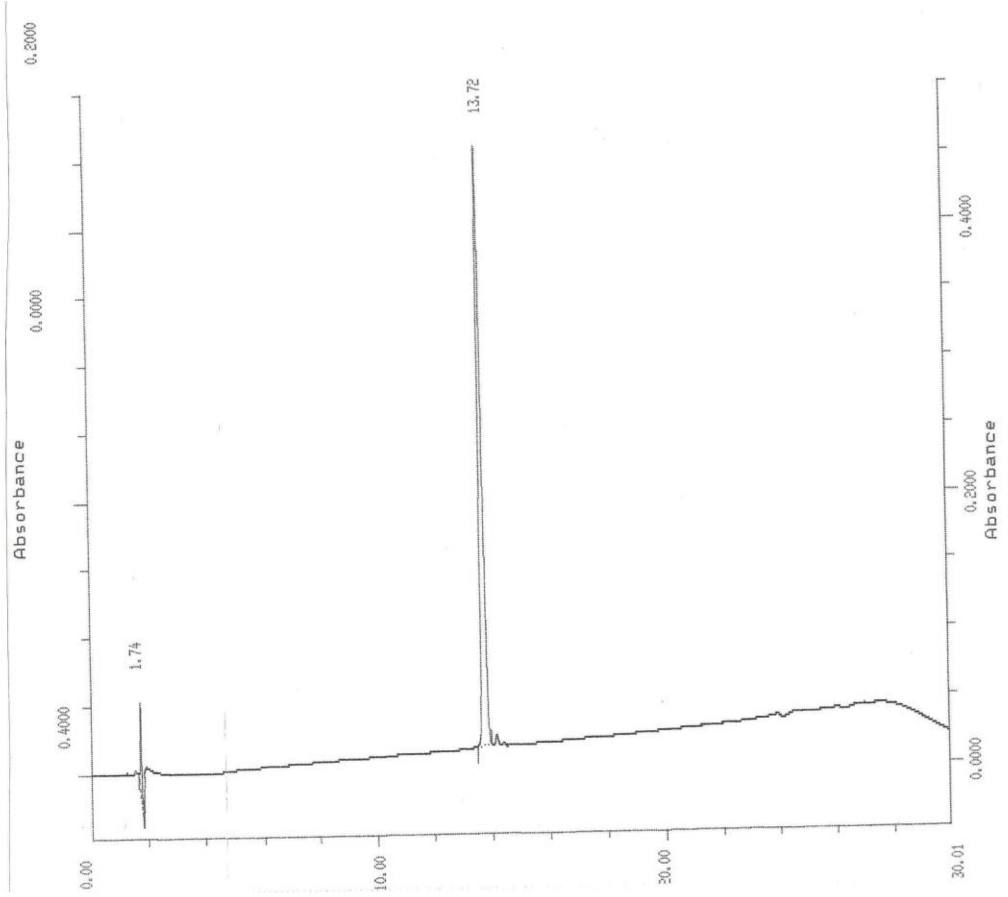

[Tyr[1],Lys(Ac)[5]]N/OFQ(1-13)NH_2

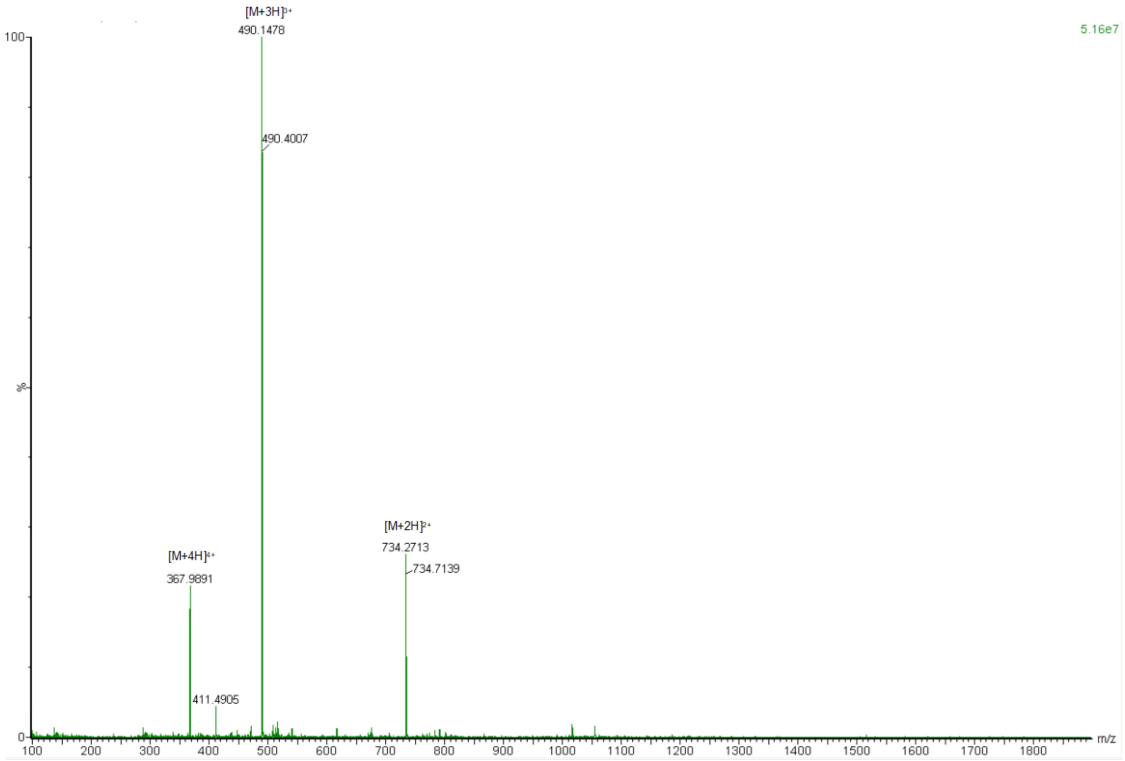

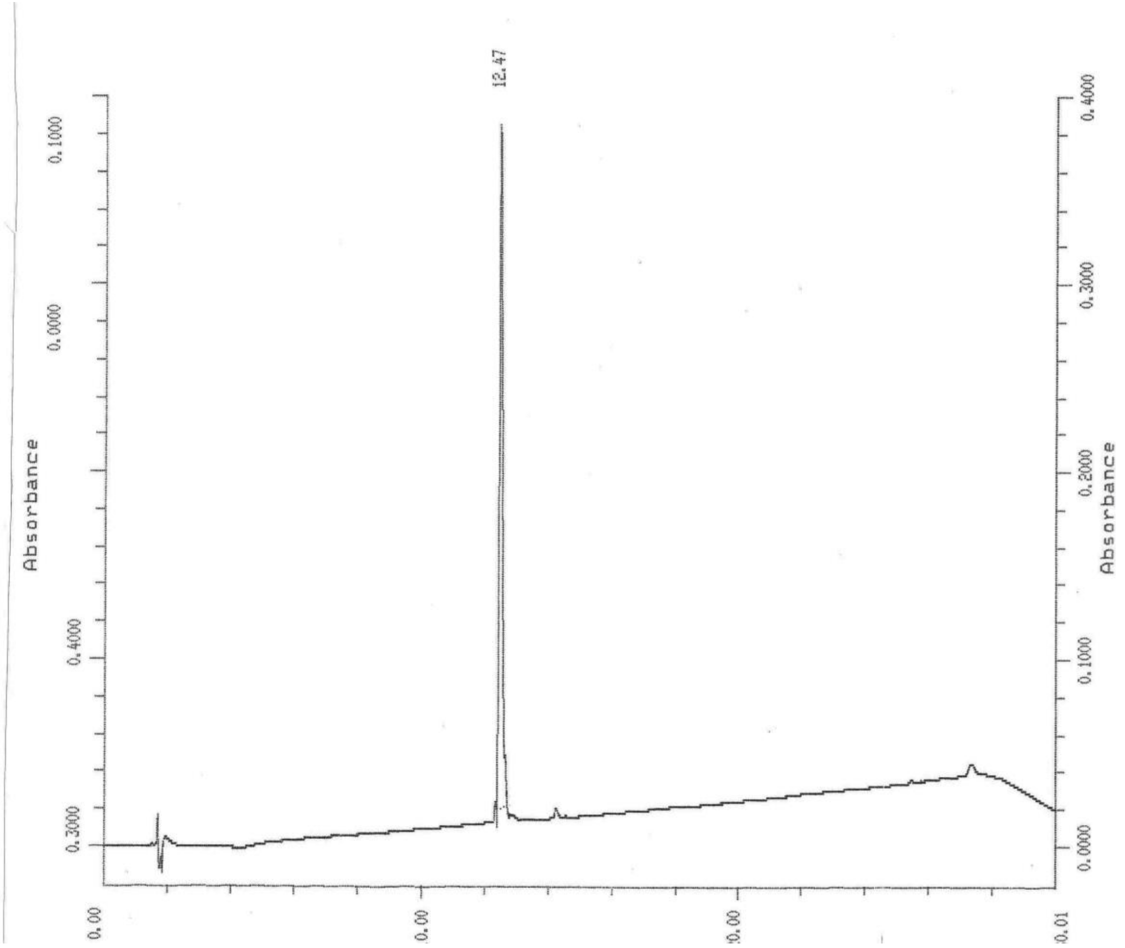

[Tyr[1],Abu[5]]N/OFQ(1-13)NH_2

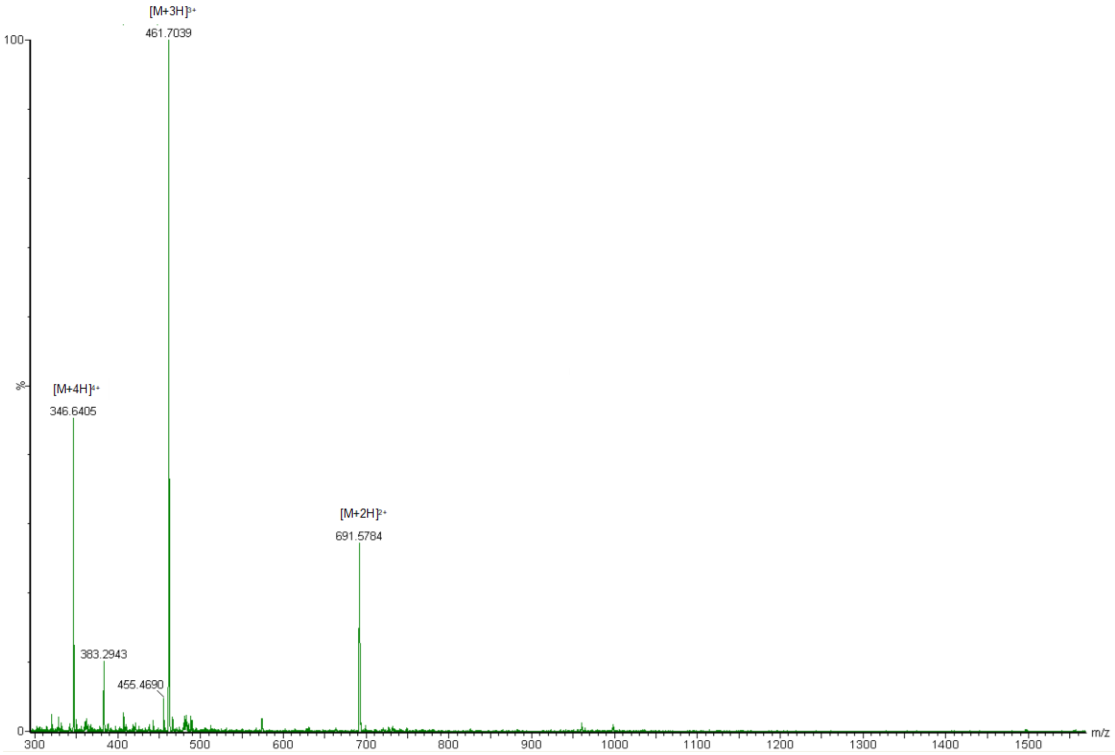

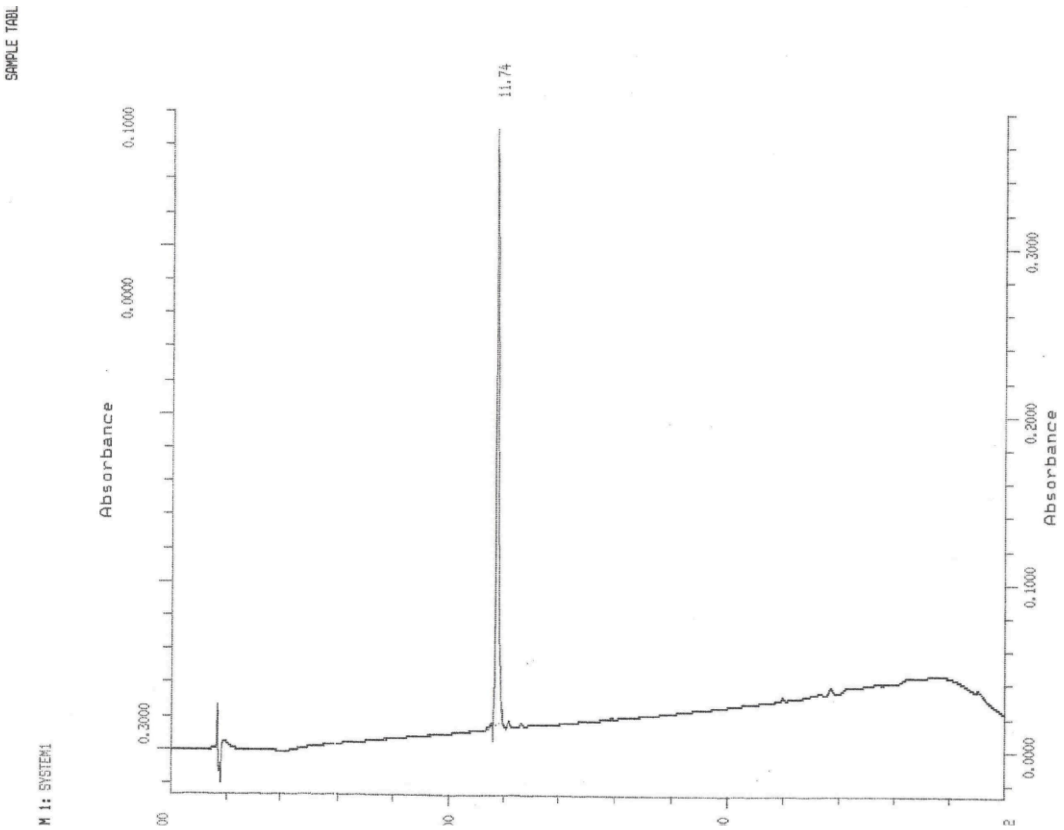

[Tyr[1],Lys[5]]N/OFQ(1-13)NH$_2$

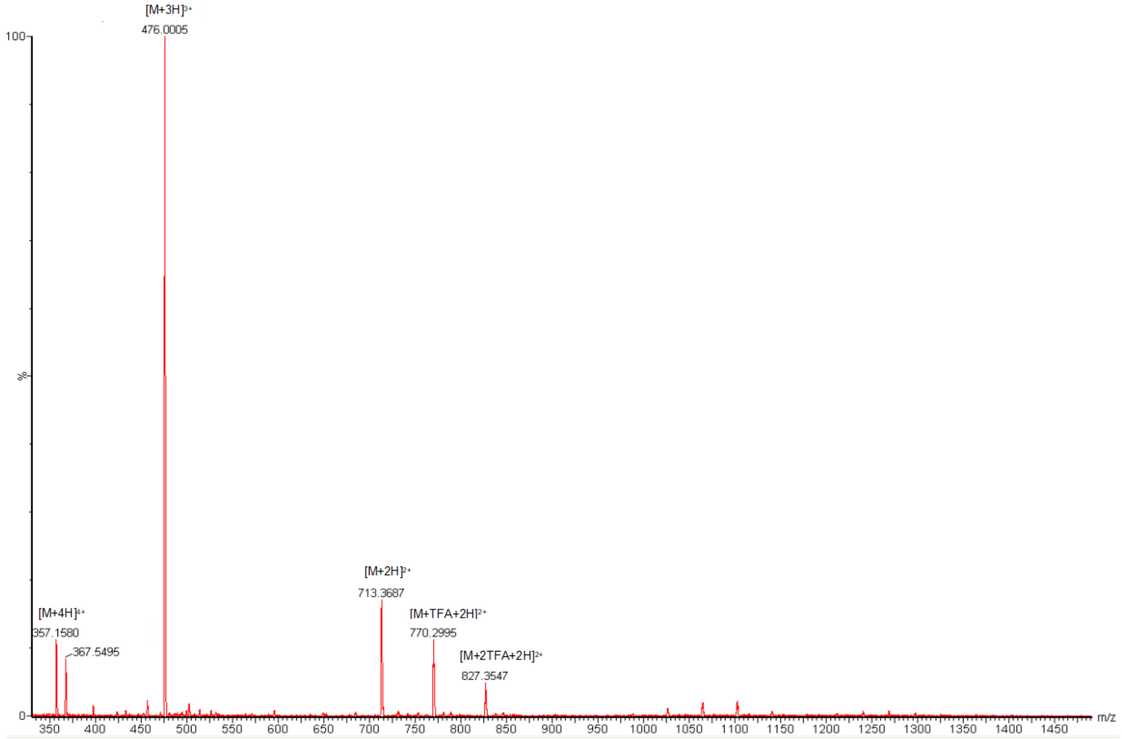

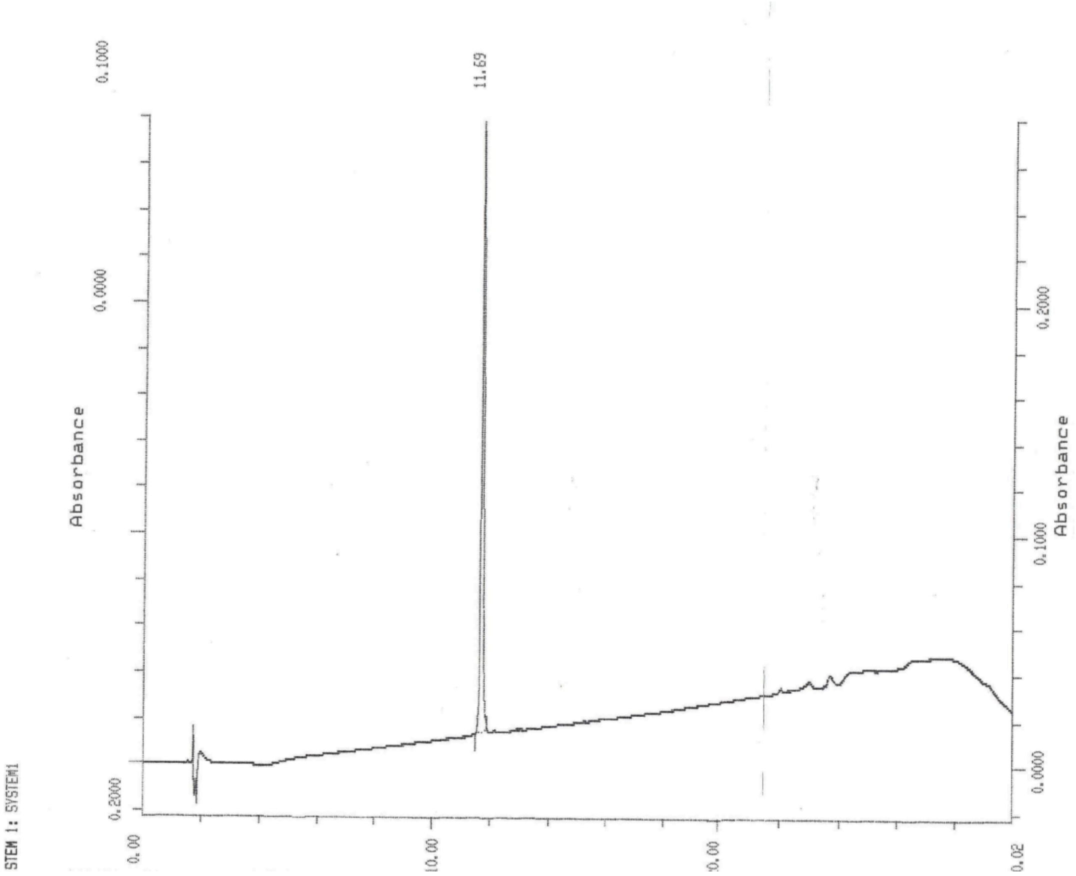

[Tyr[1],Dap[5]]N/OFQ(1-13)NH_2

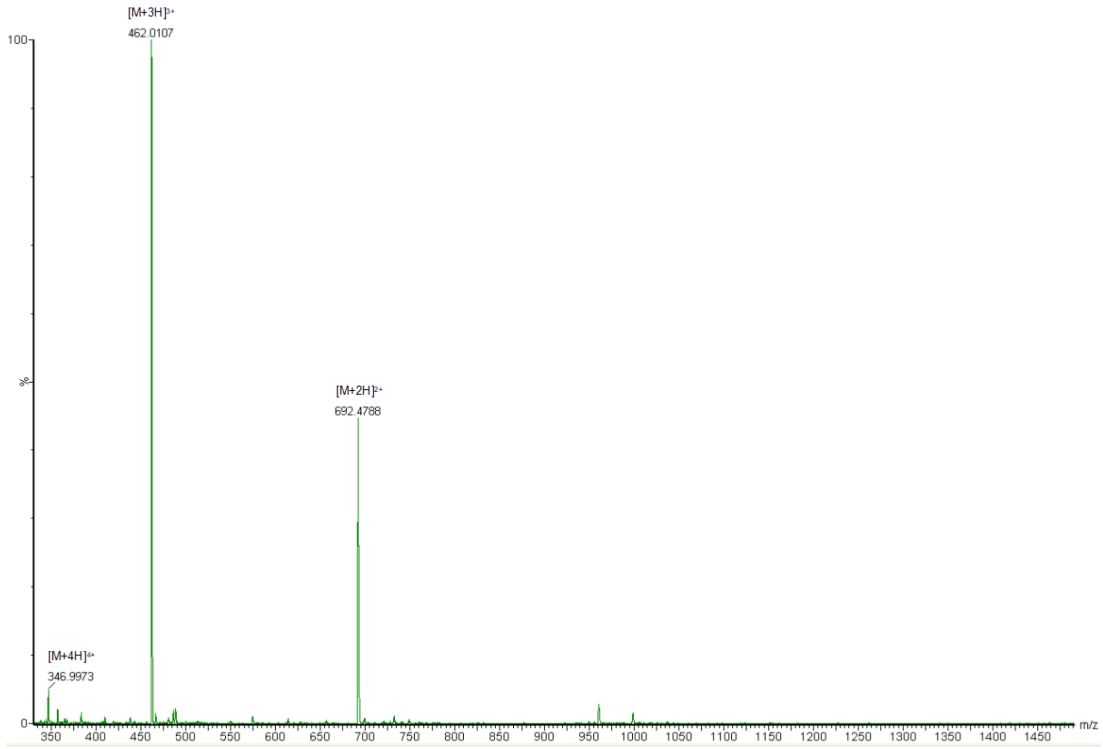

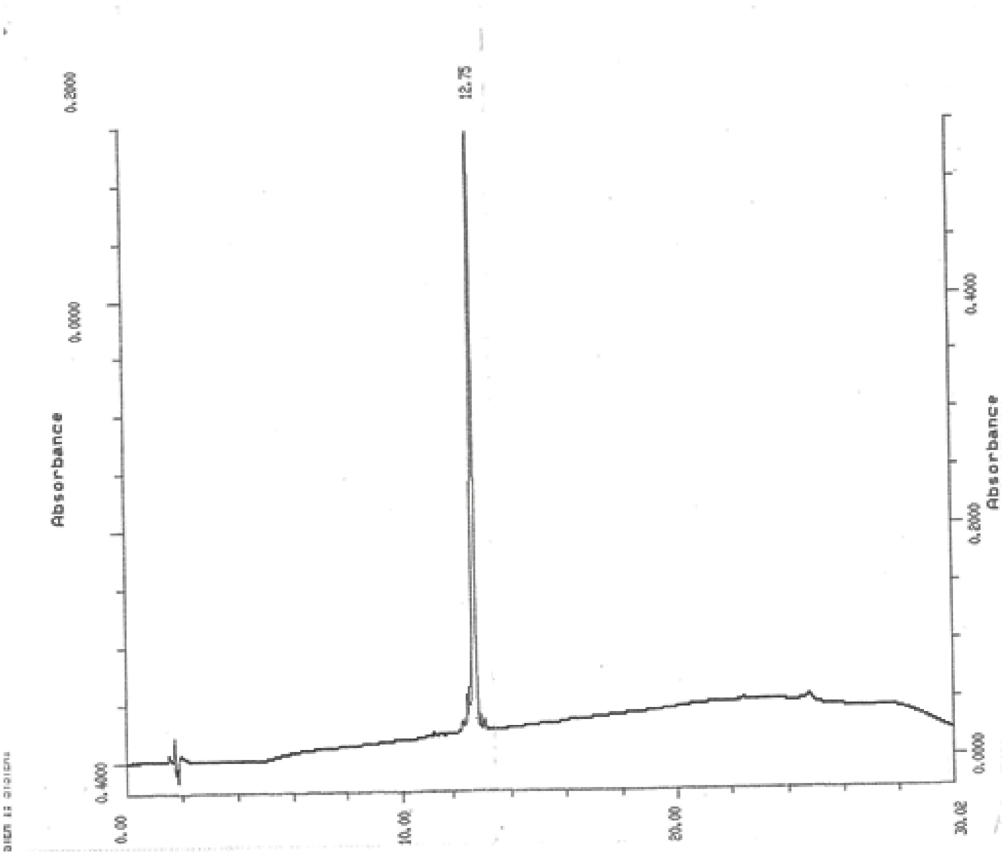

[Tyr[1],Dab[5]]N/OFQ(1-13)NH_2

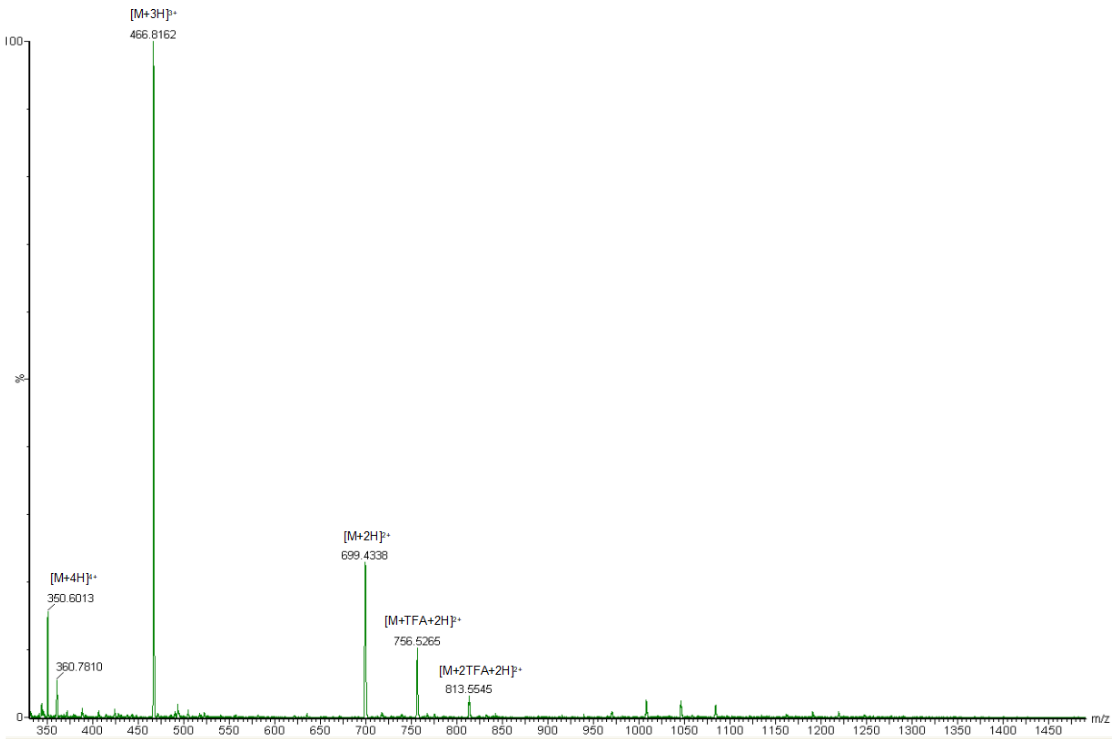

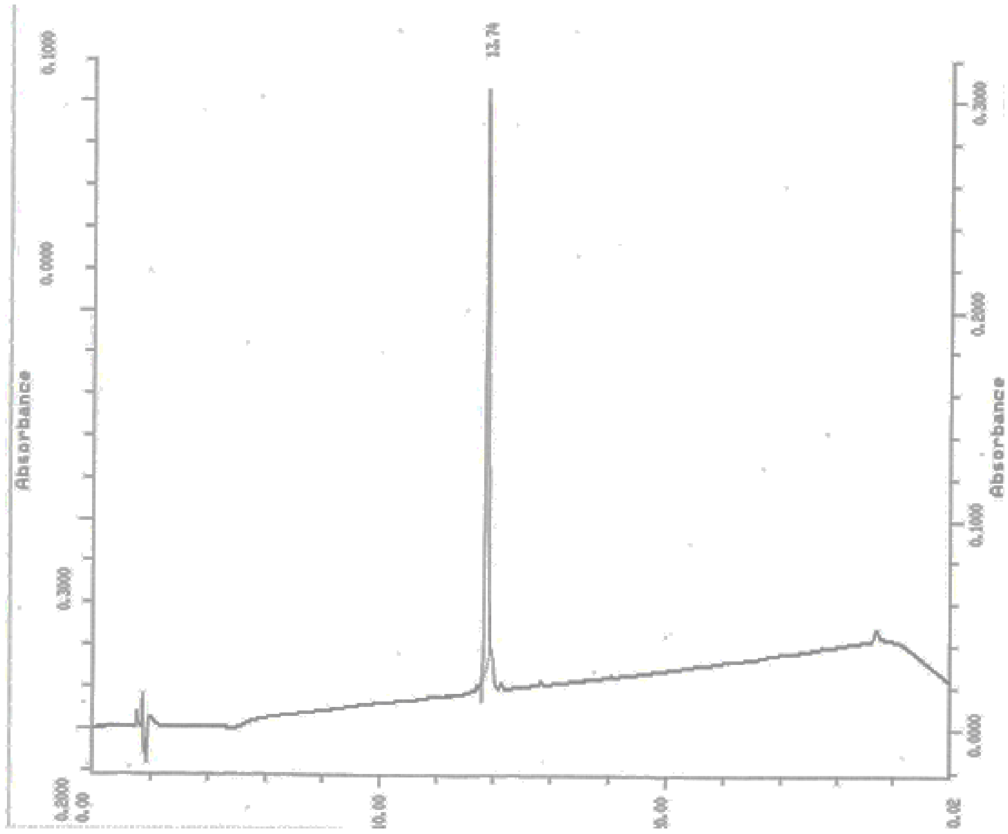

[Tyr[1],Leu[5]]N/OFQ(1-13)NH_2

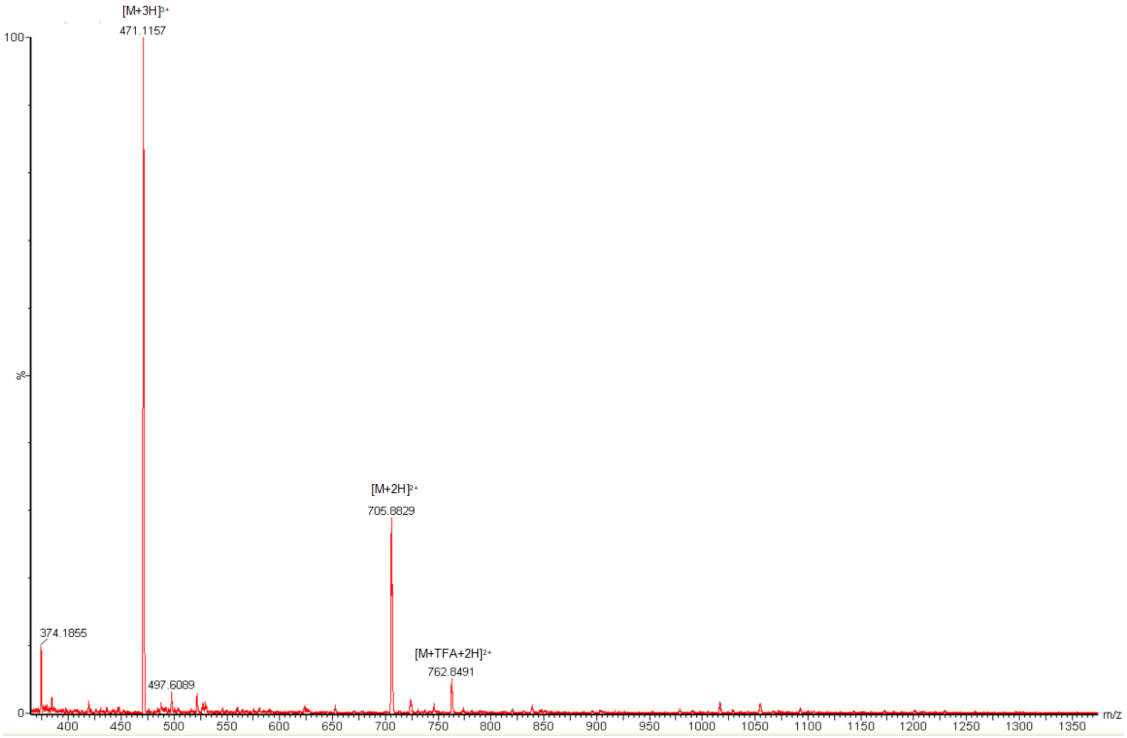

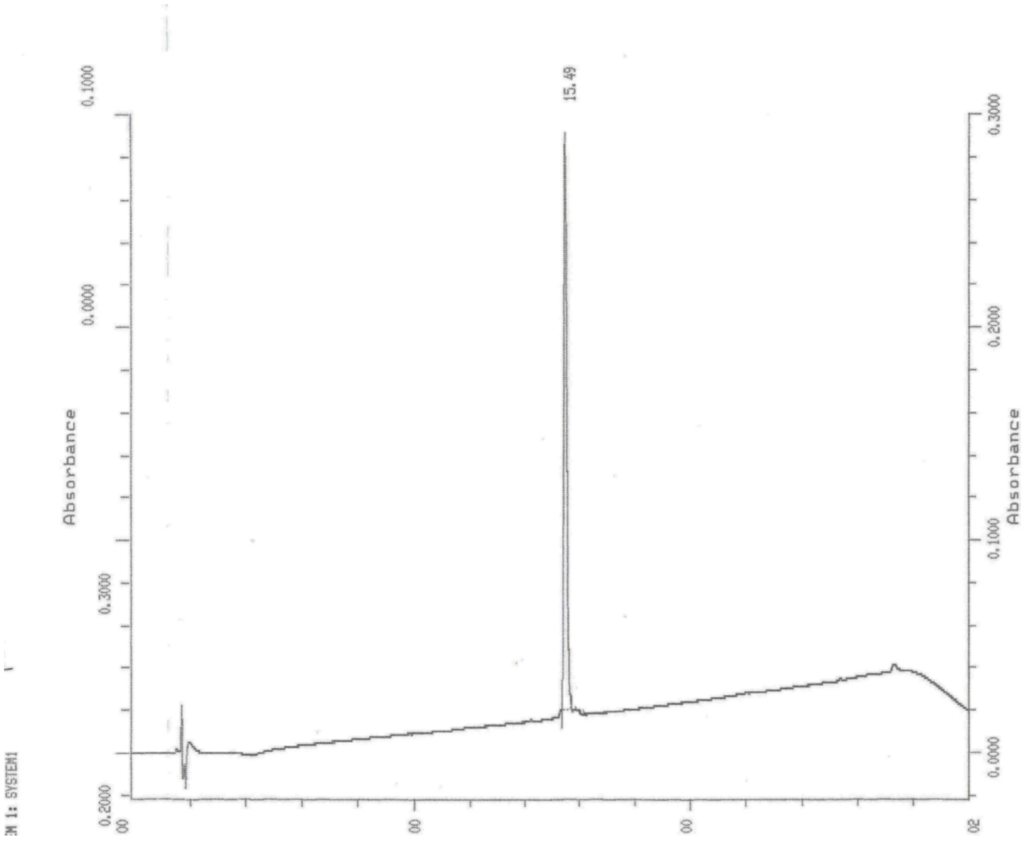

[Tyr[1],Nle[5]]N/OFQ(1-13)NH_2

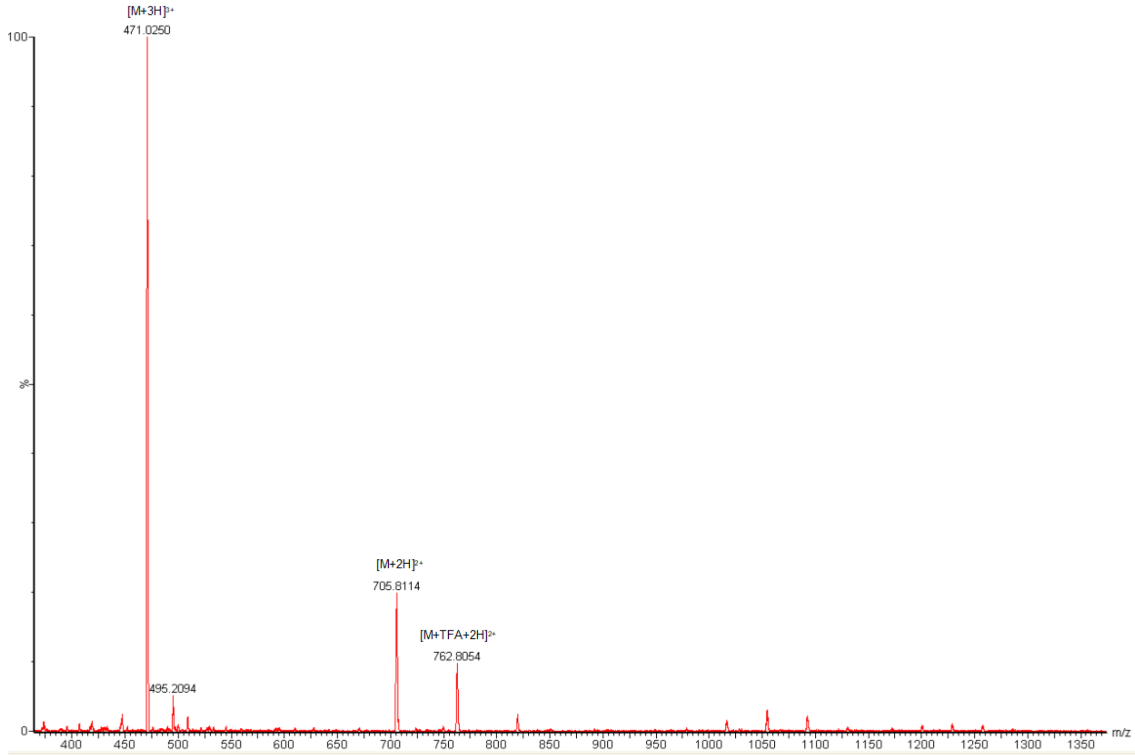

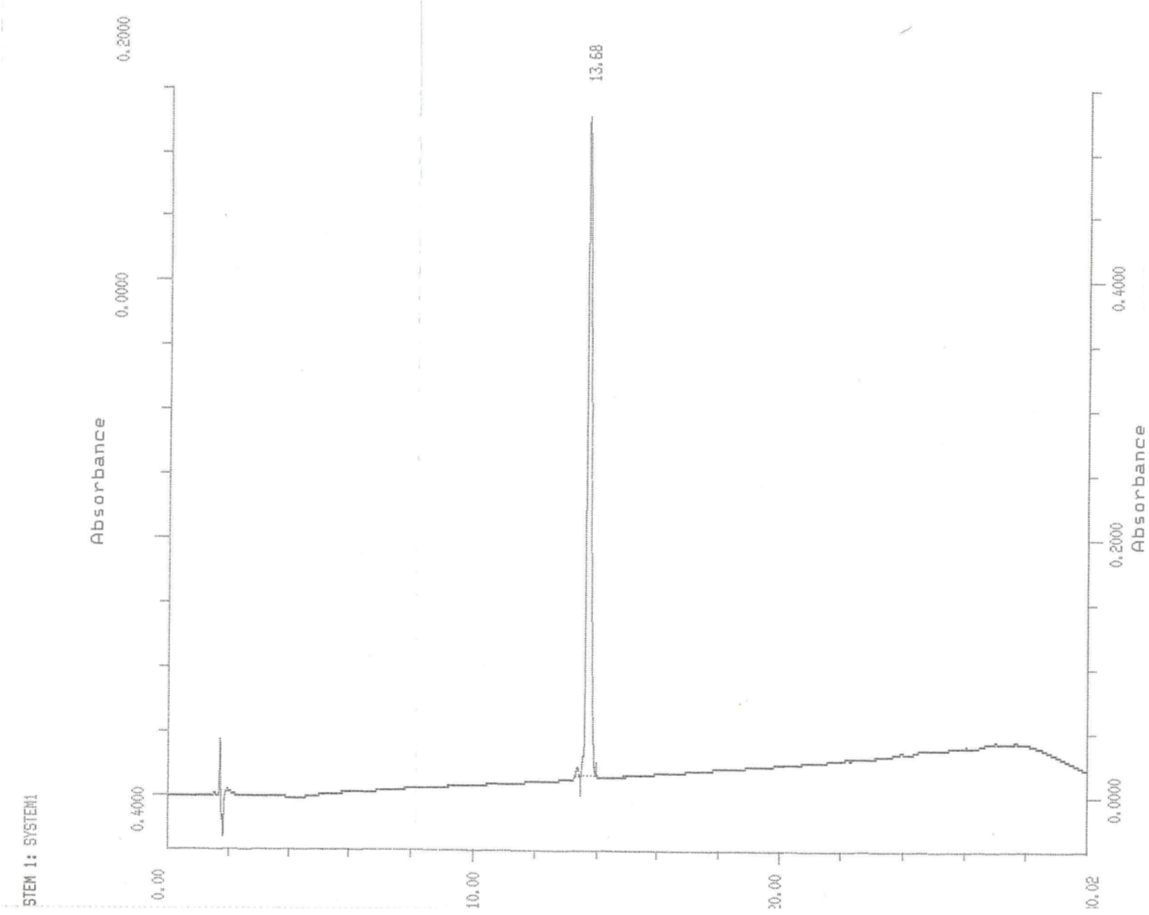

[Tyr[1],Nva[5]]N/OFQ(1-13)NH$_2$

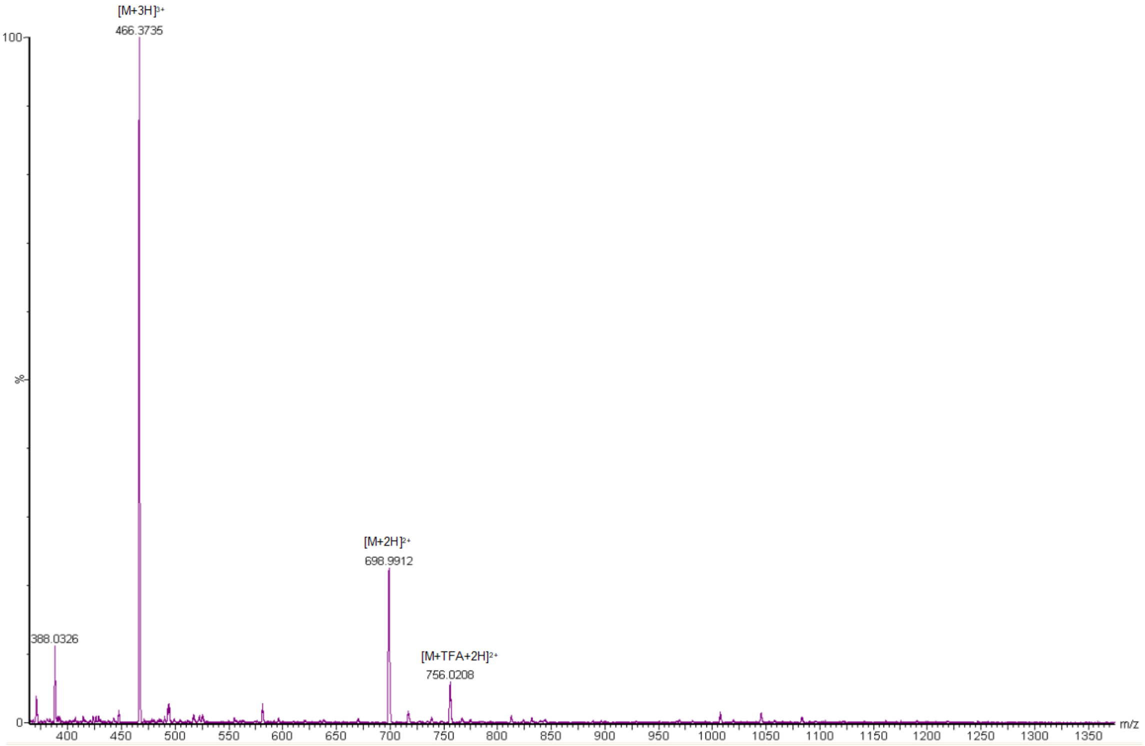

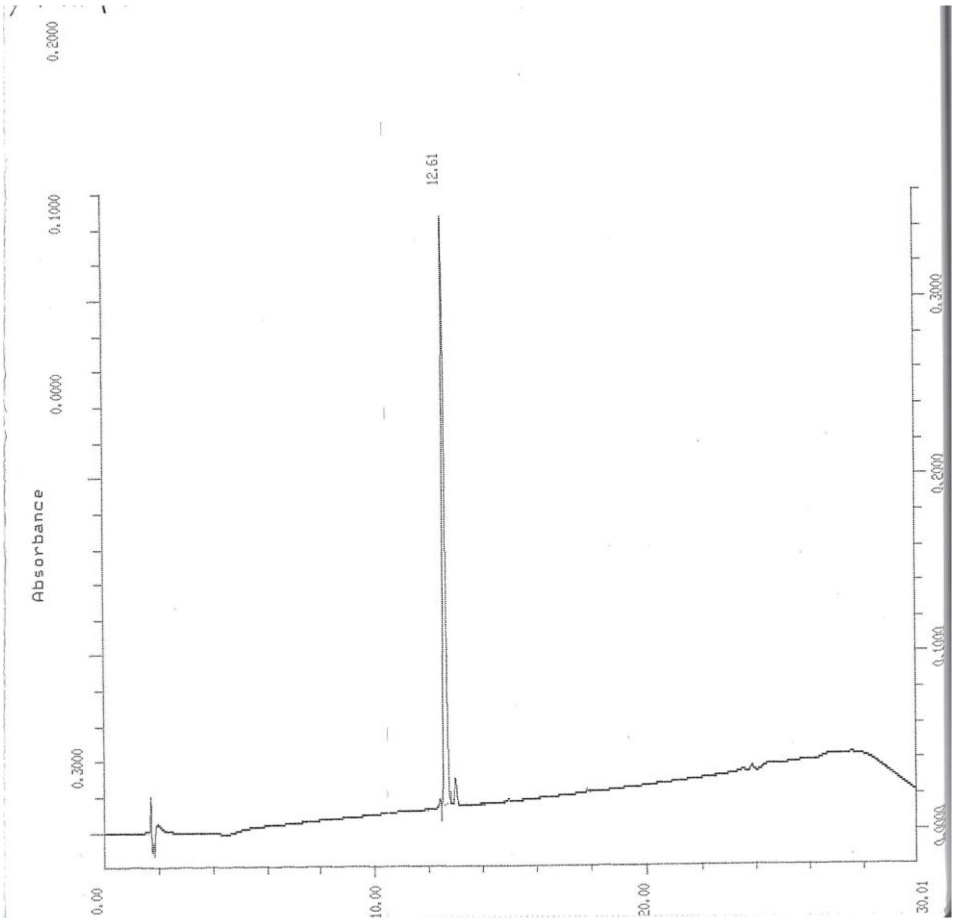

[Tyr[1],Tyr[5]]N/OFQ(1-13)NH$_2$

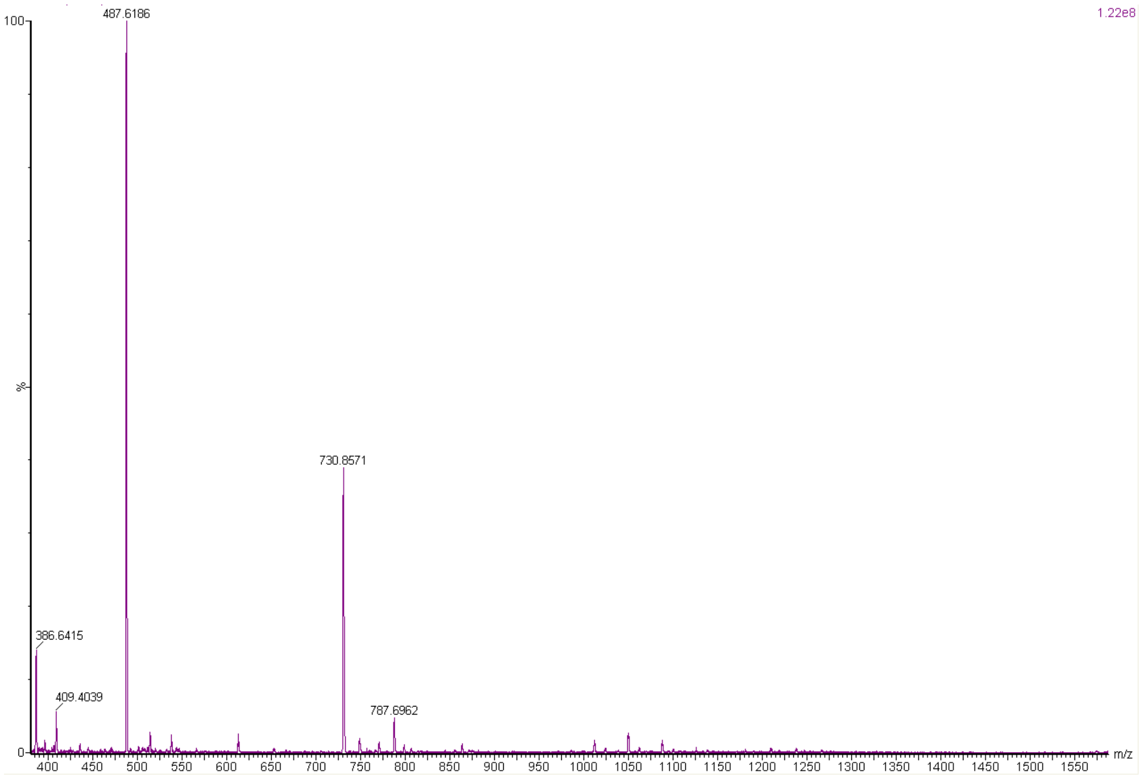

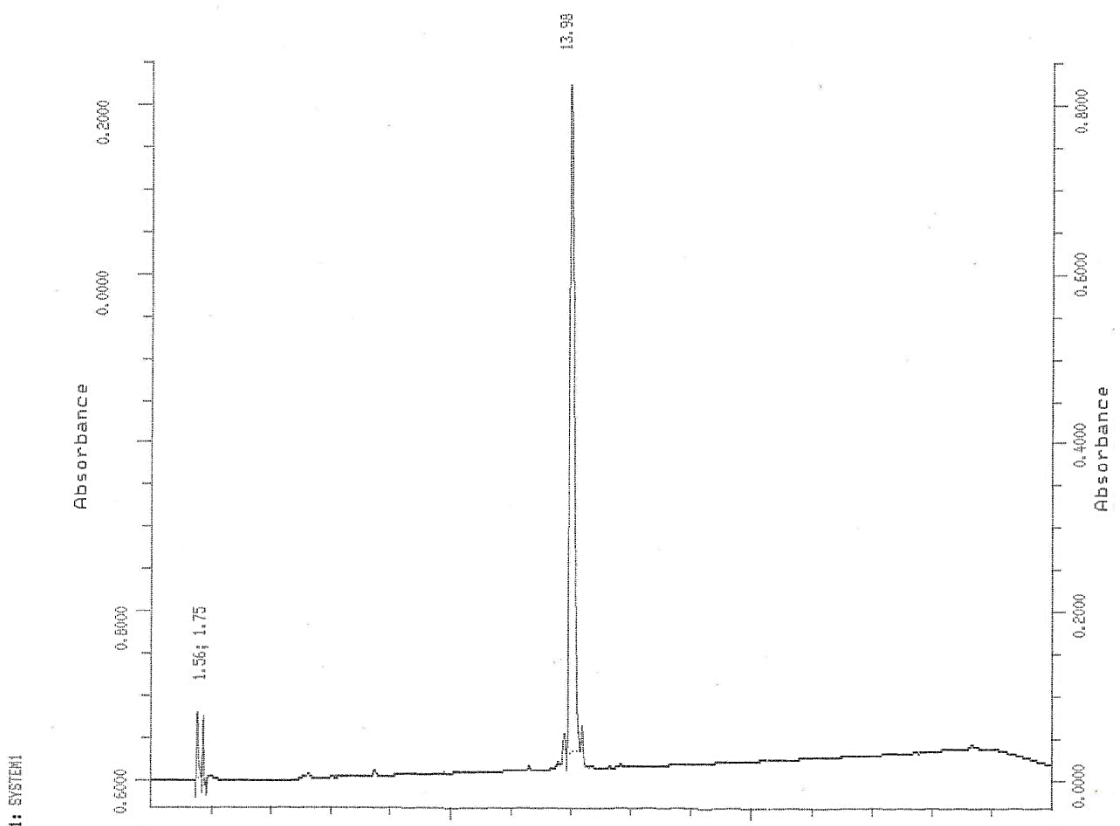

[Tyr[1],Phe[5]]N/OFQ(1-13)NH_2

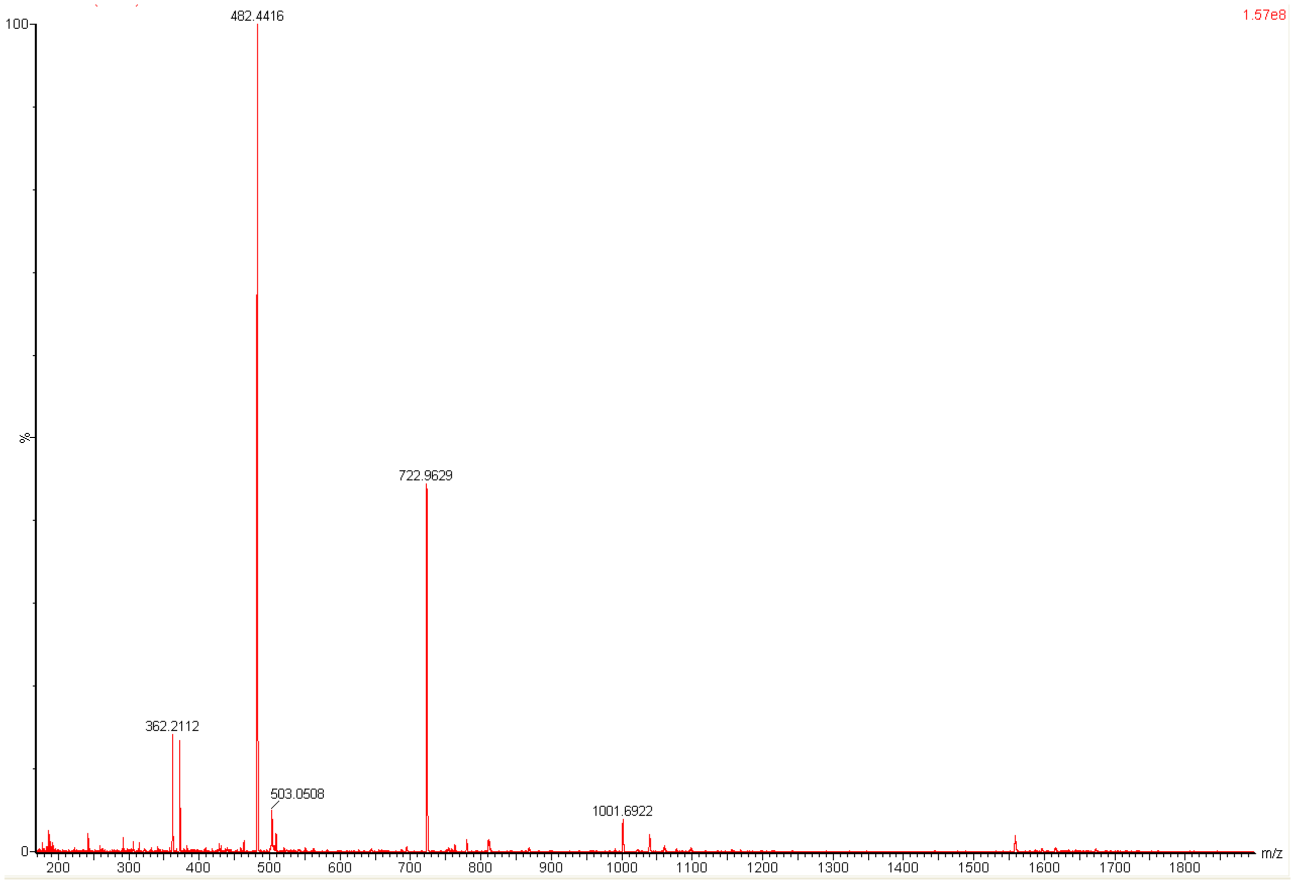

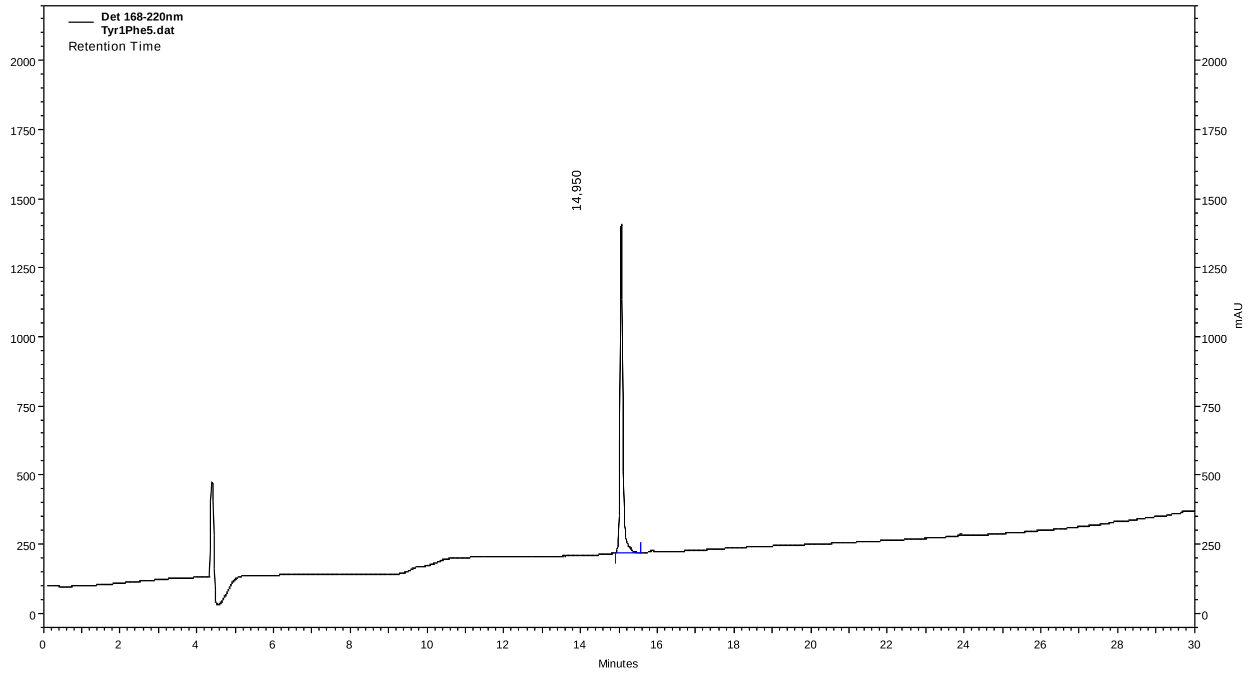

[Tyr[1],His[5]]N/OFQ(1-13)NH_2

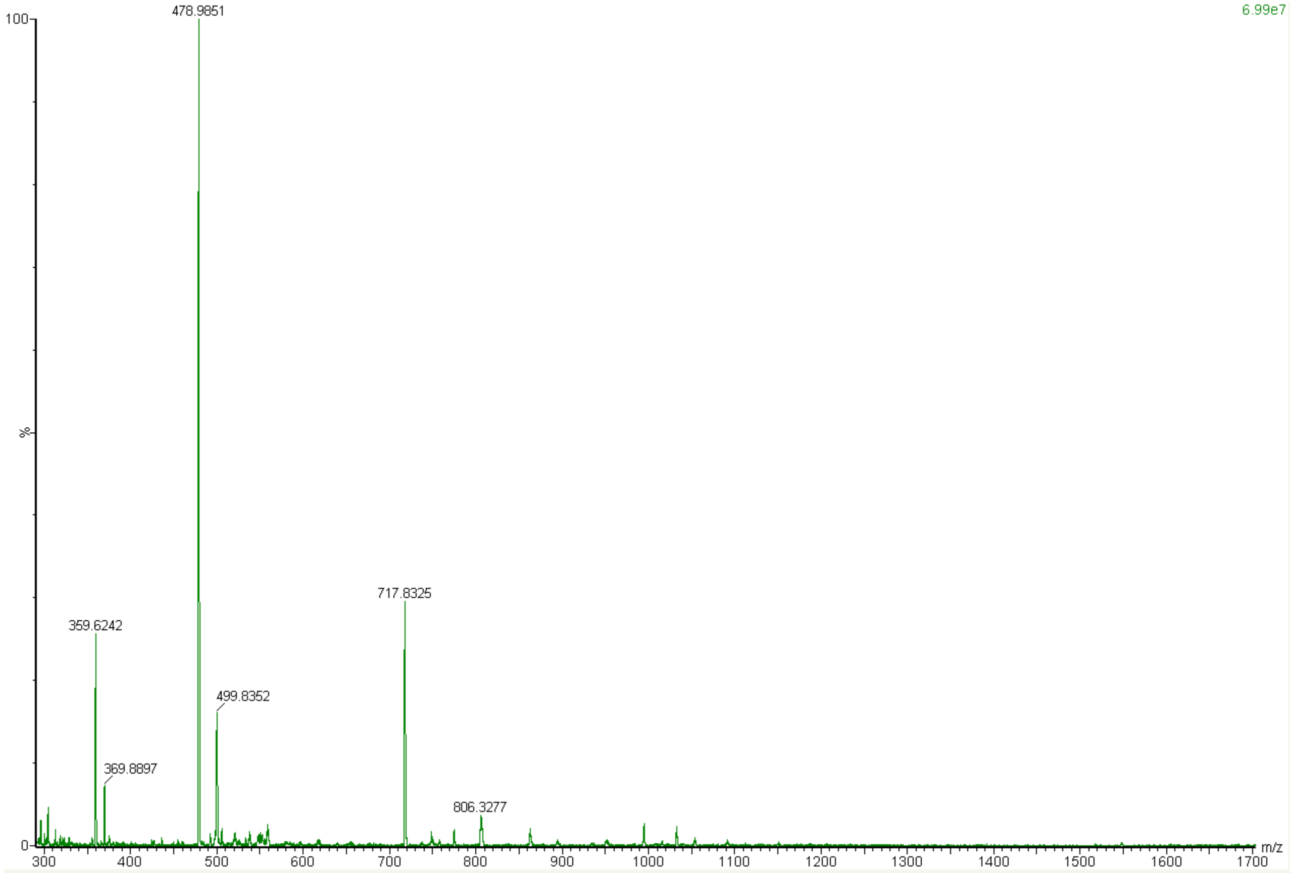

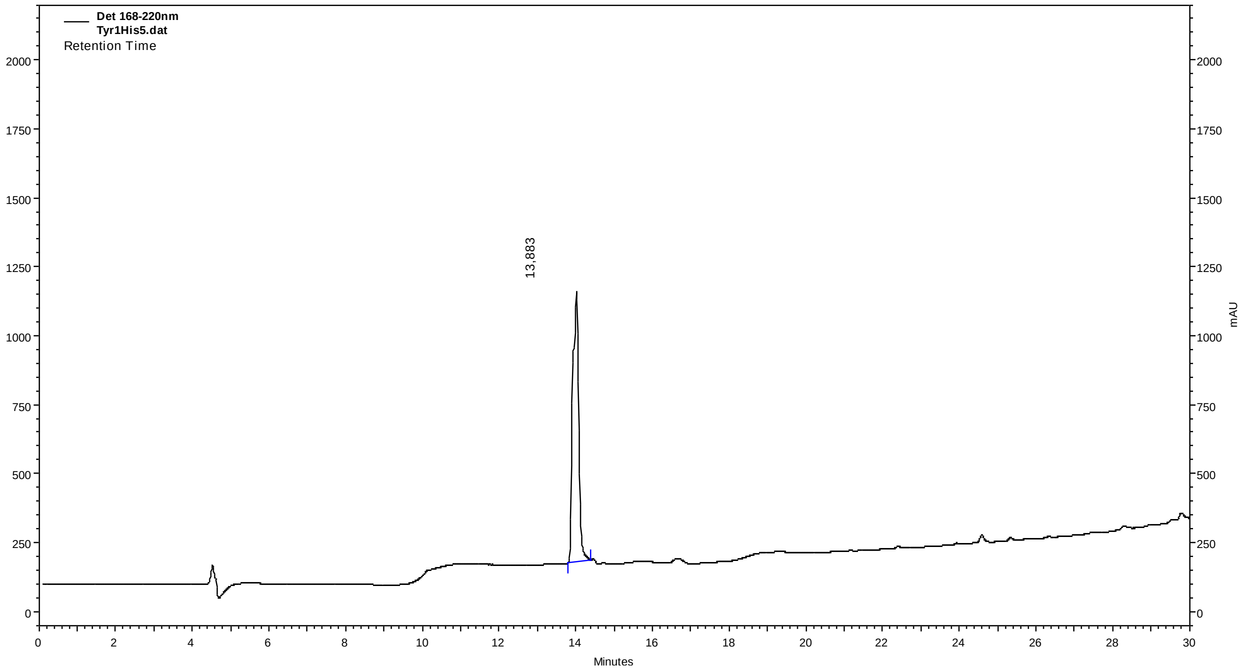

[Tyr[1],Trp[5]]N/OFQ(1-13)NH_2

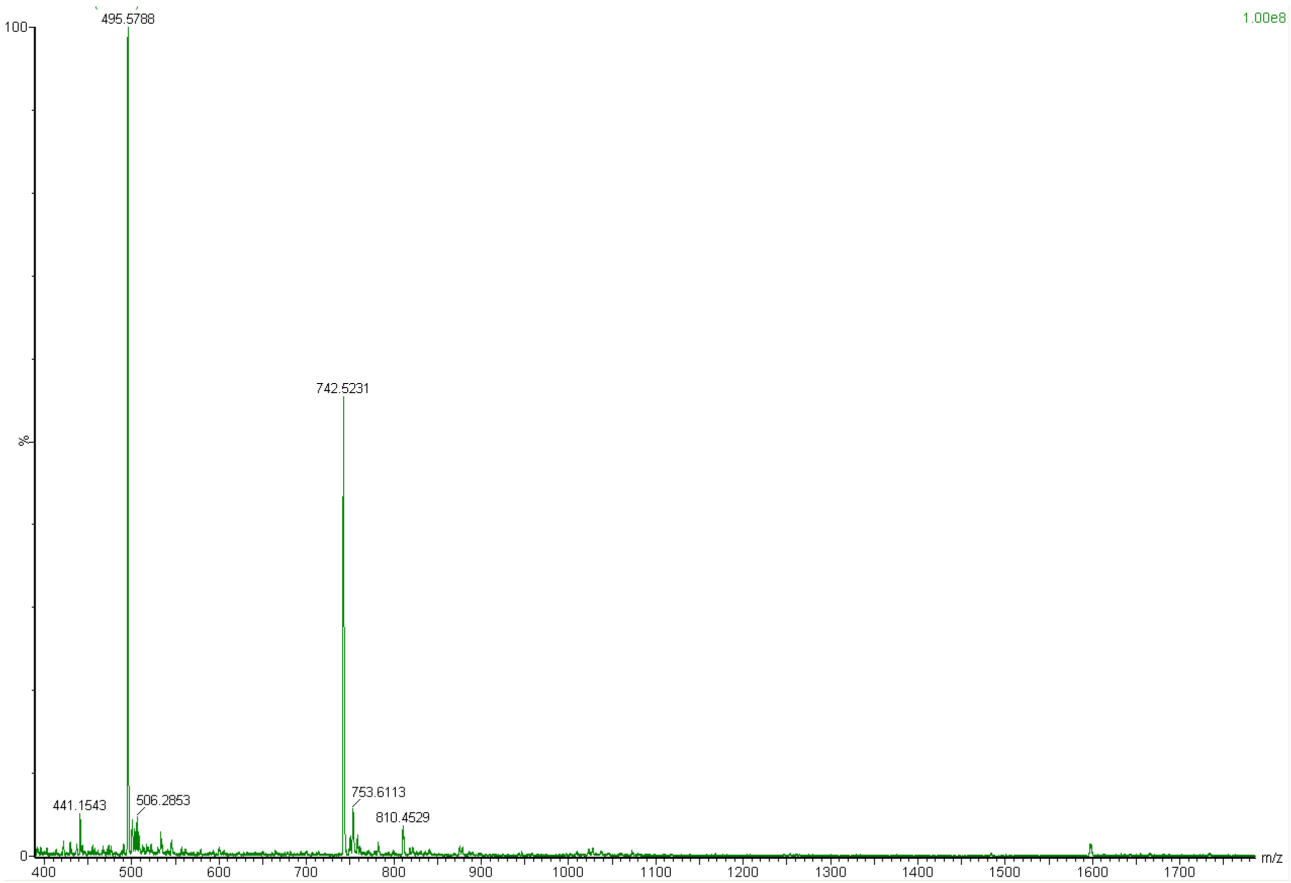

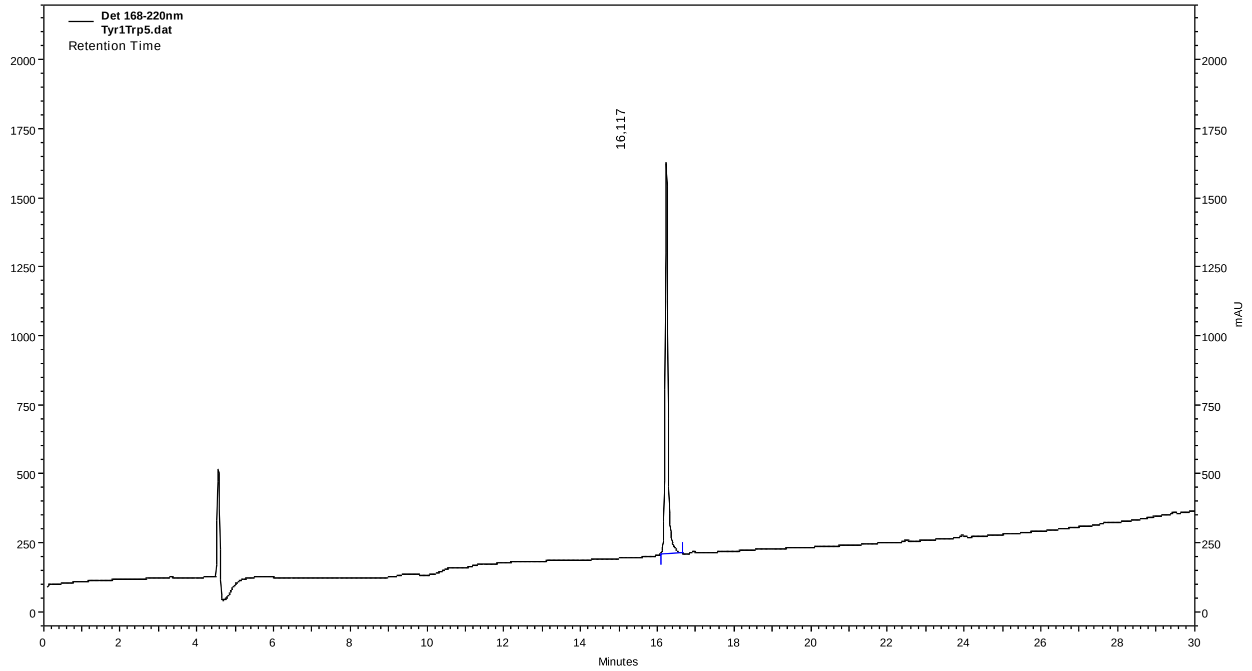

[Tyr[1],hPhe[5]]N/OFQ(1-13)NH_2

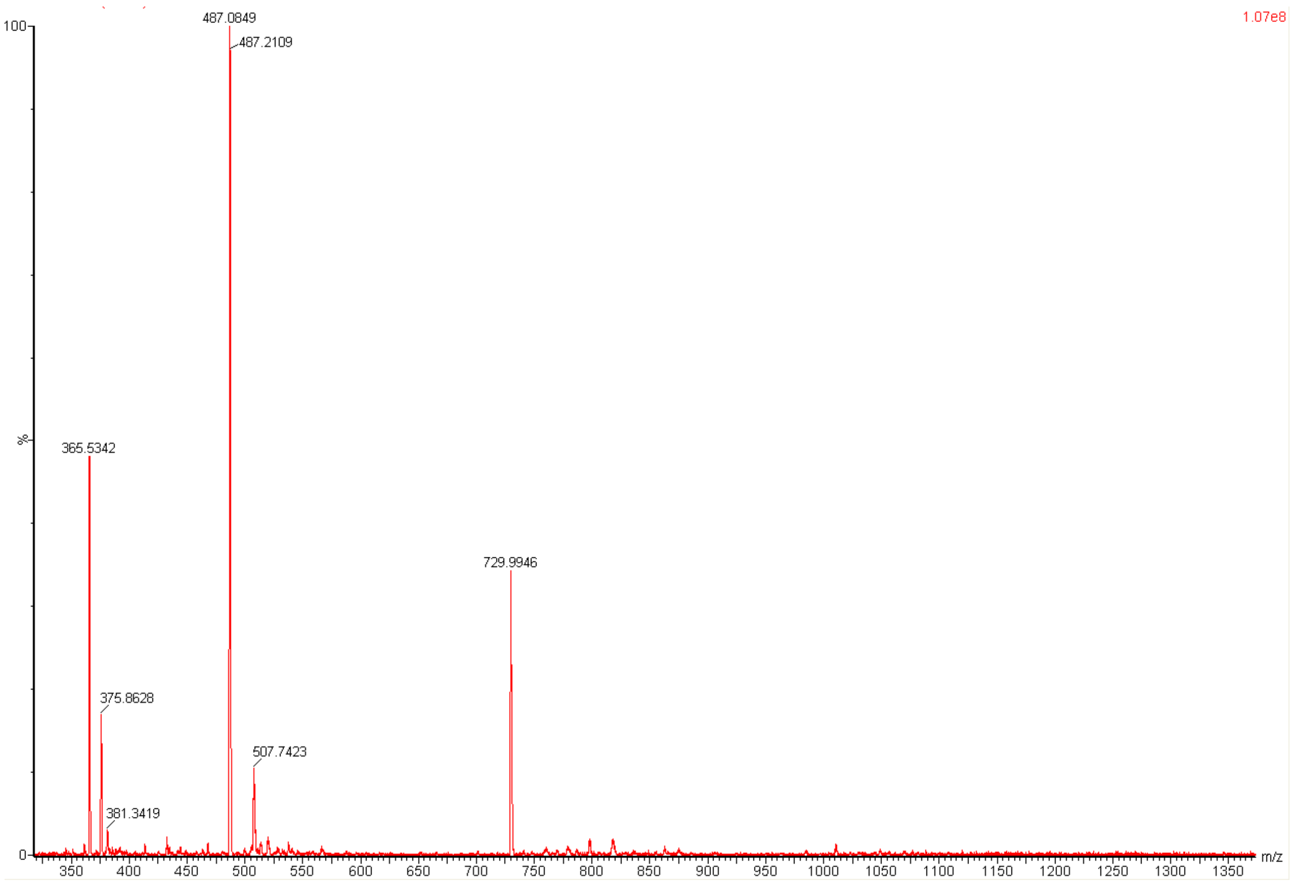

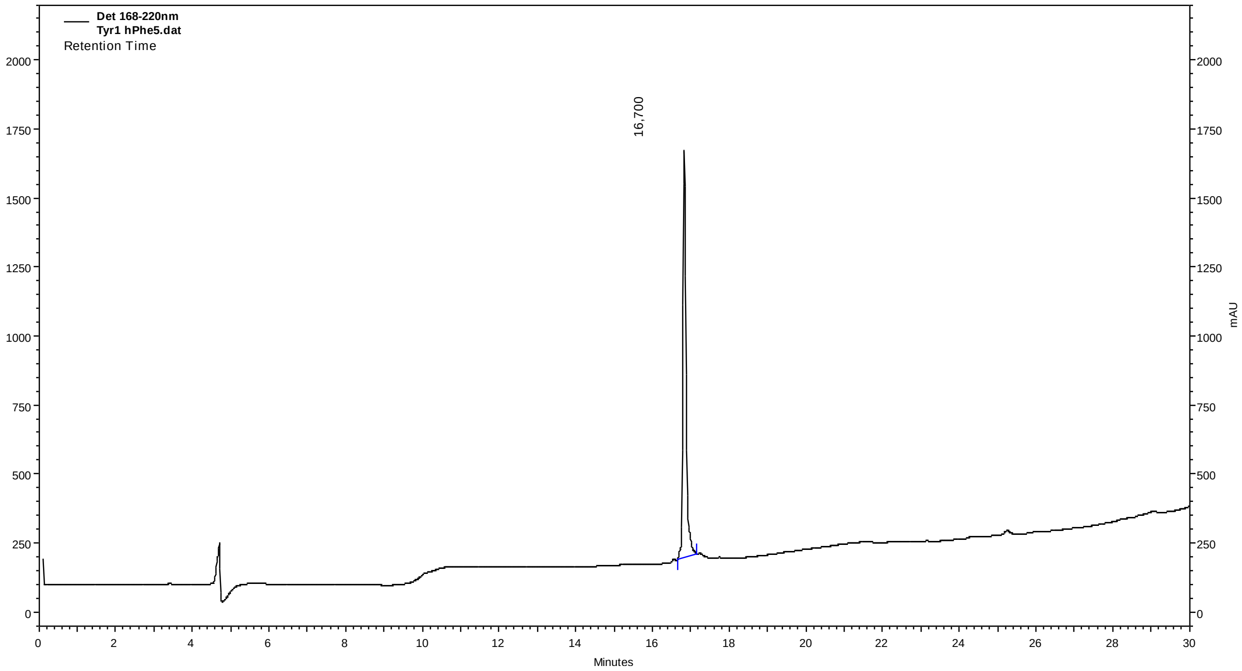

[Tyr[1],Phg[5]]N/OFQ(1-13)NH_2

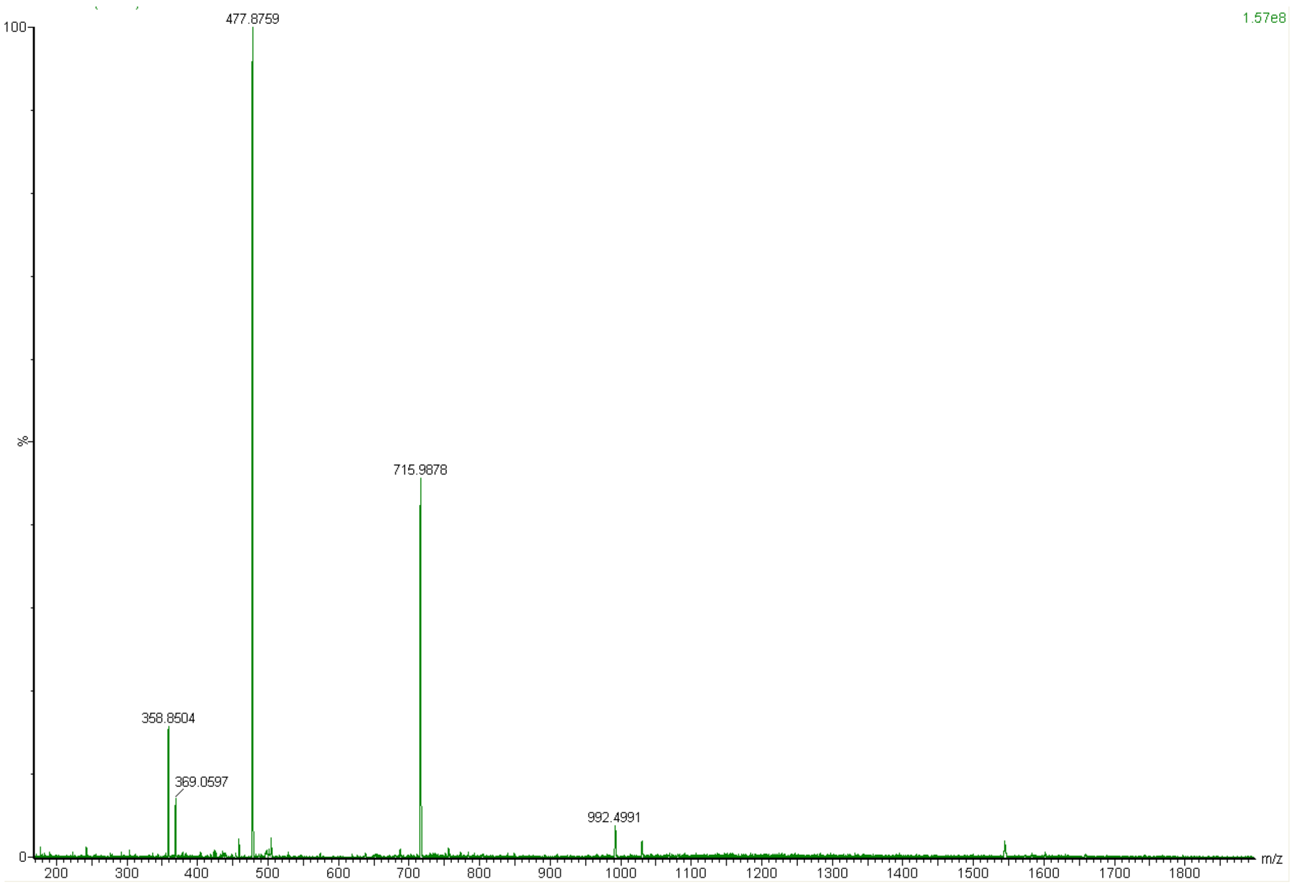

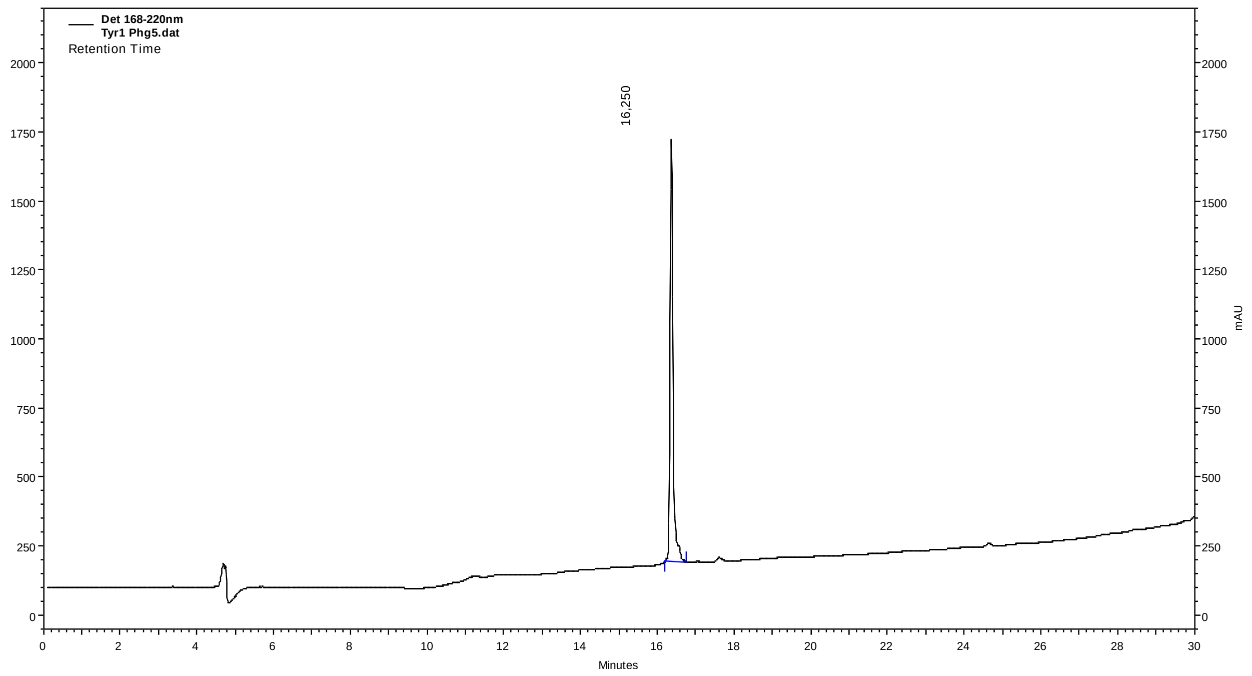

[Tyr[1],p(OCH_3)Phe[5]]N/OFQ(1-13)NH_2

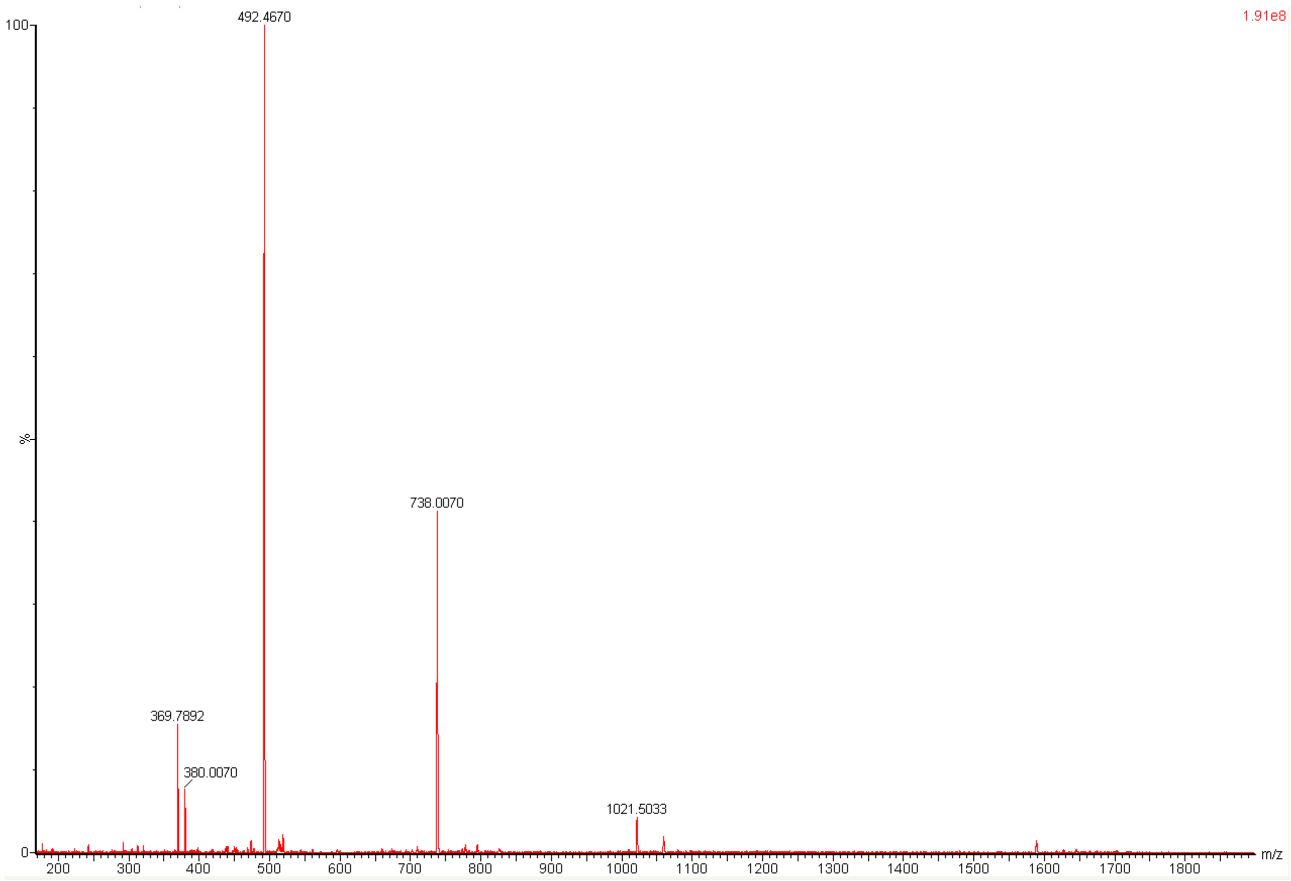

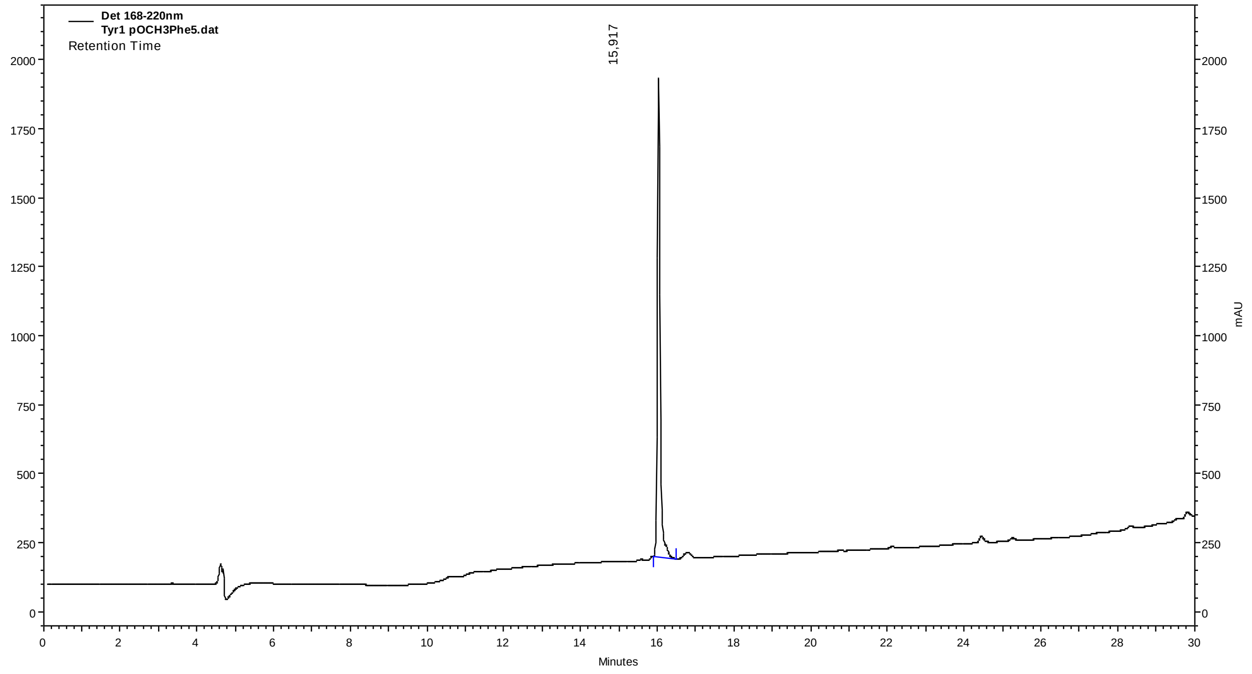

[Tyr[1],pFPhe[5]]N/OFQ(1-13)NH$_2$

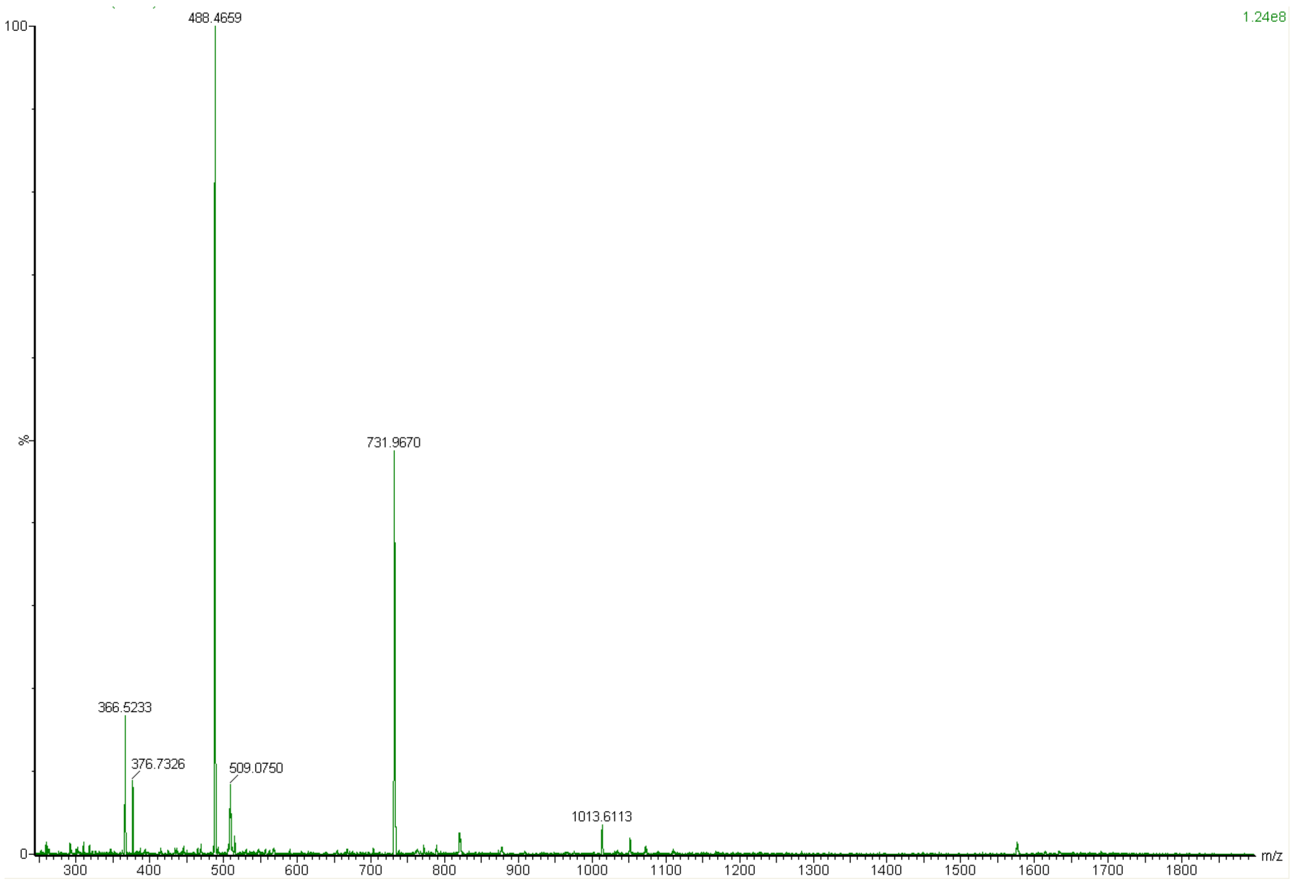

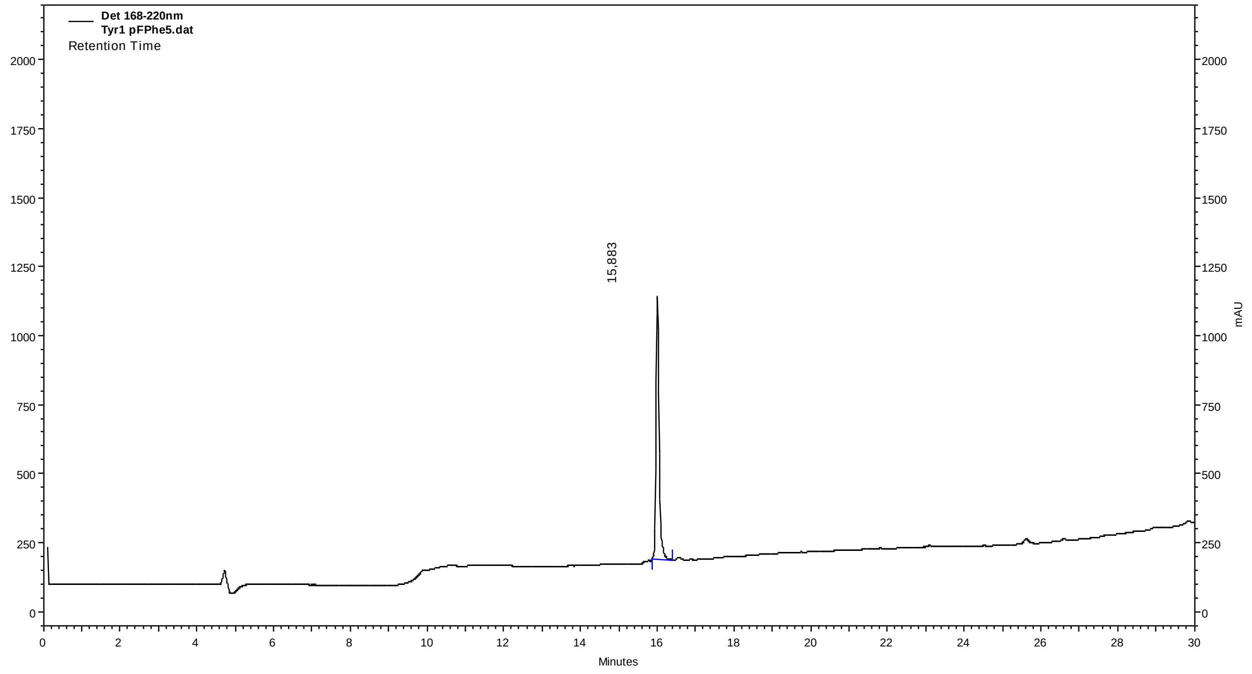

[Tyr[1],p(NO_2)Phe[5]]N/OFQ(1-13)NH_2

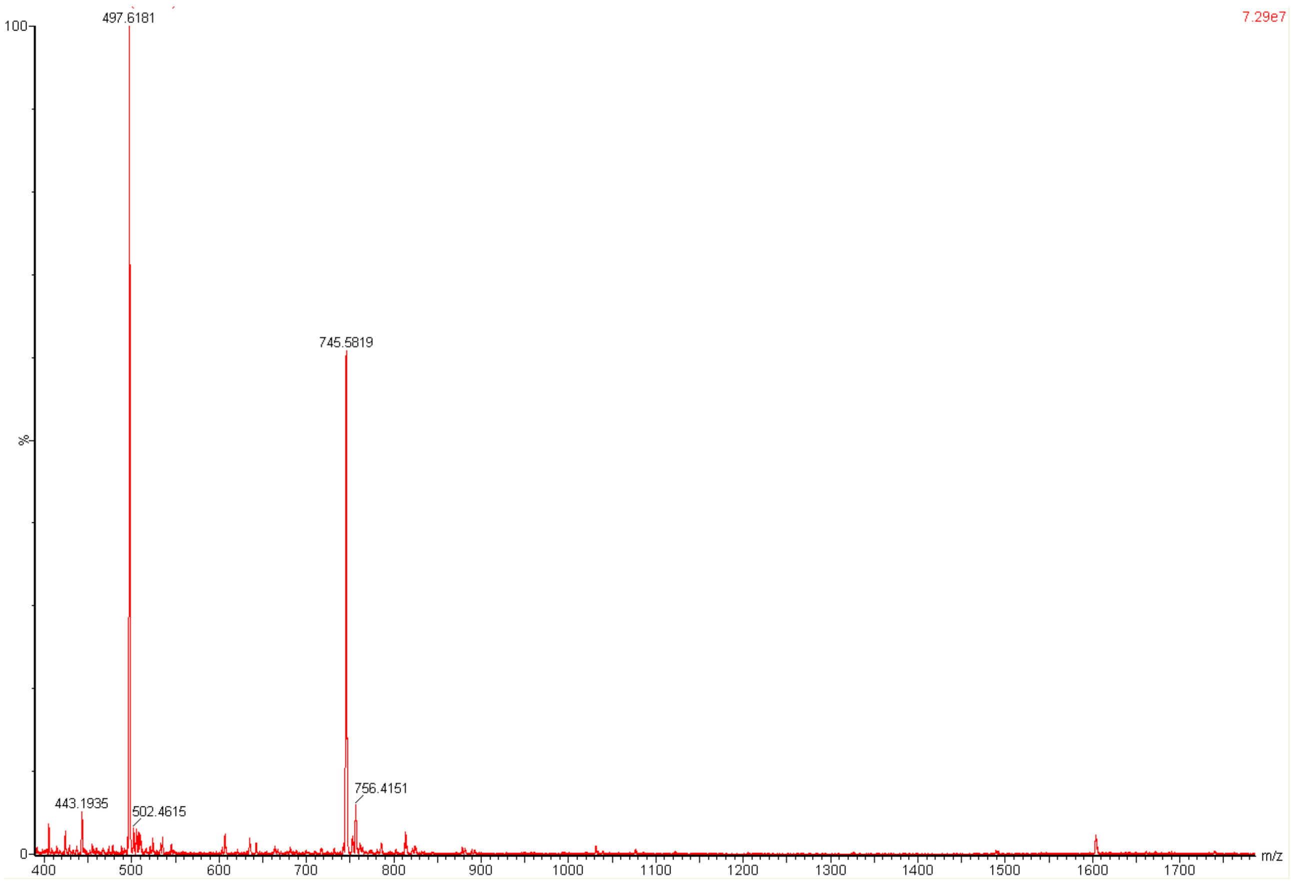

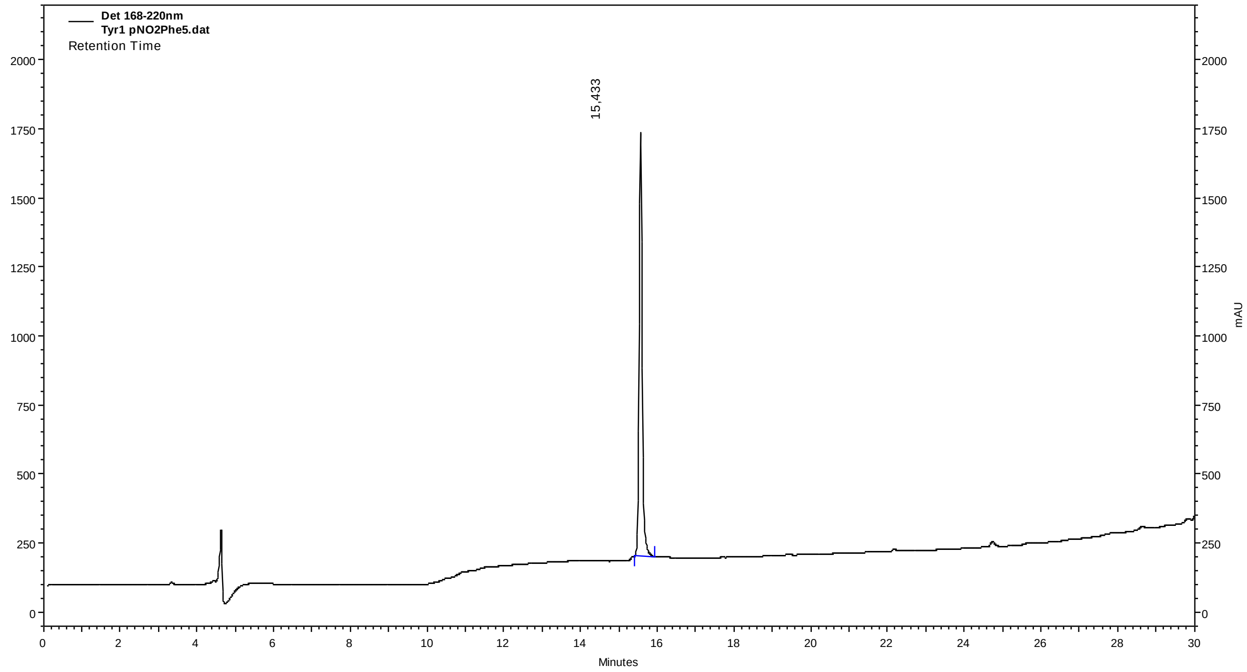

[Tyr[1],Dip[5]]N/OFQ(1-13)NH$_2$

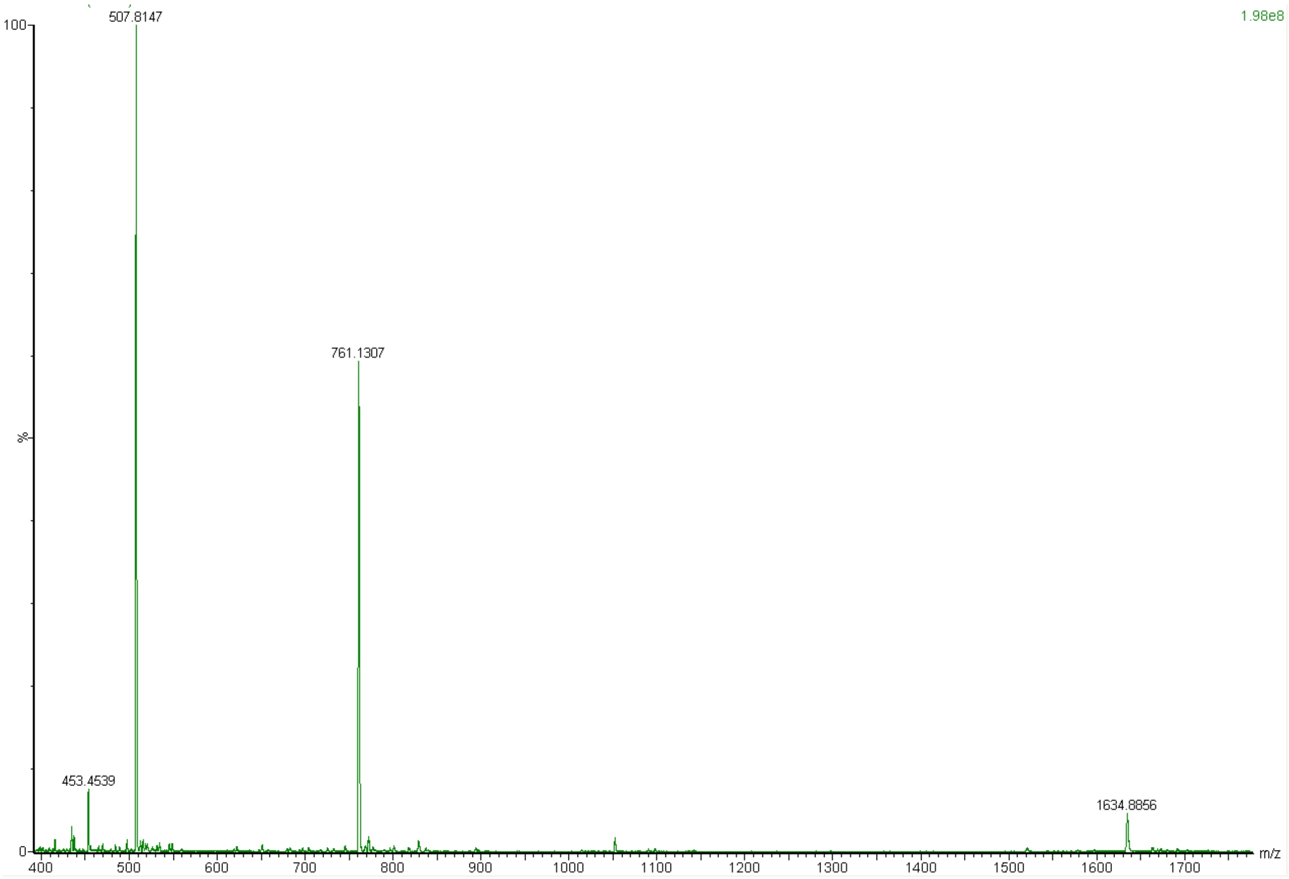

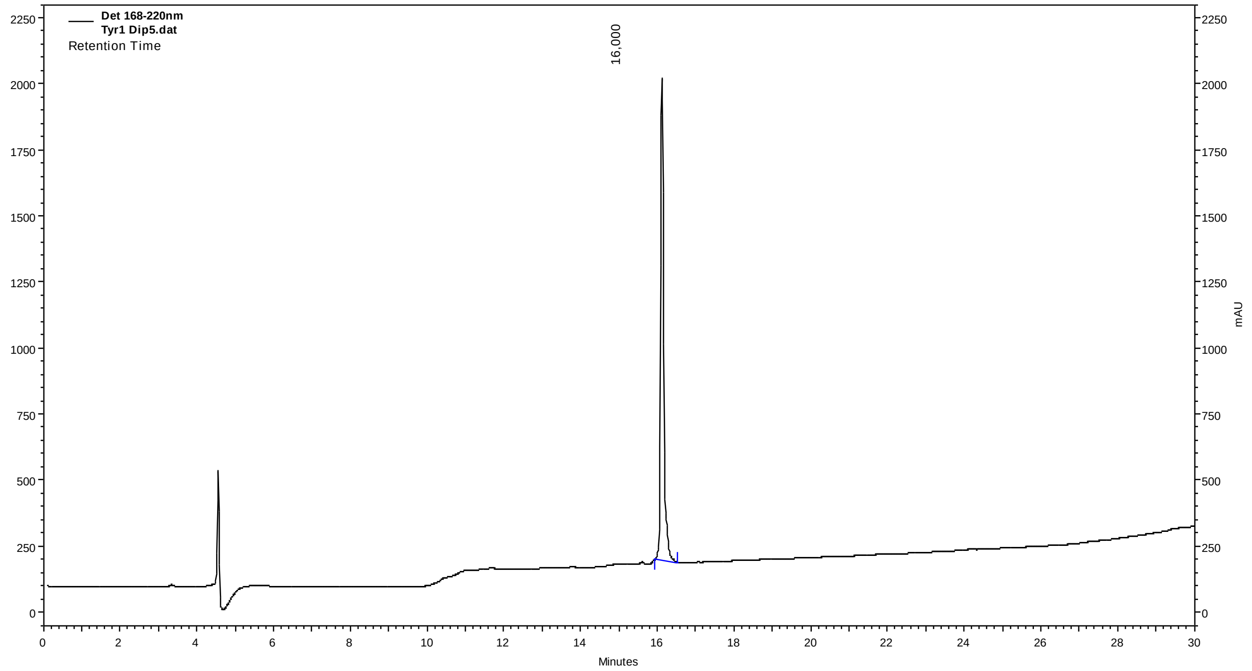

[Tyr^1,Bip^5]N/OFQ(1-13)NH_2

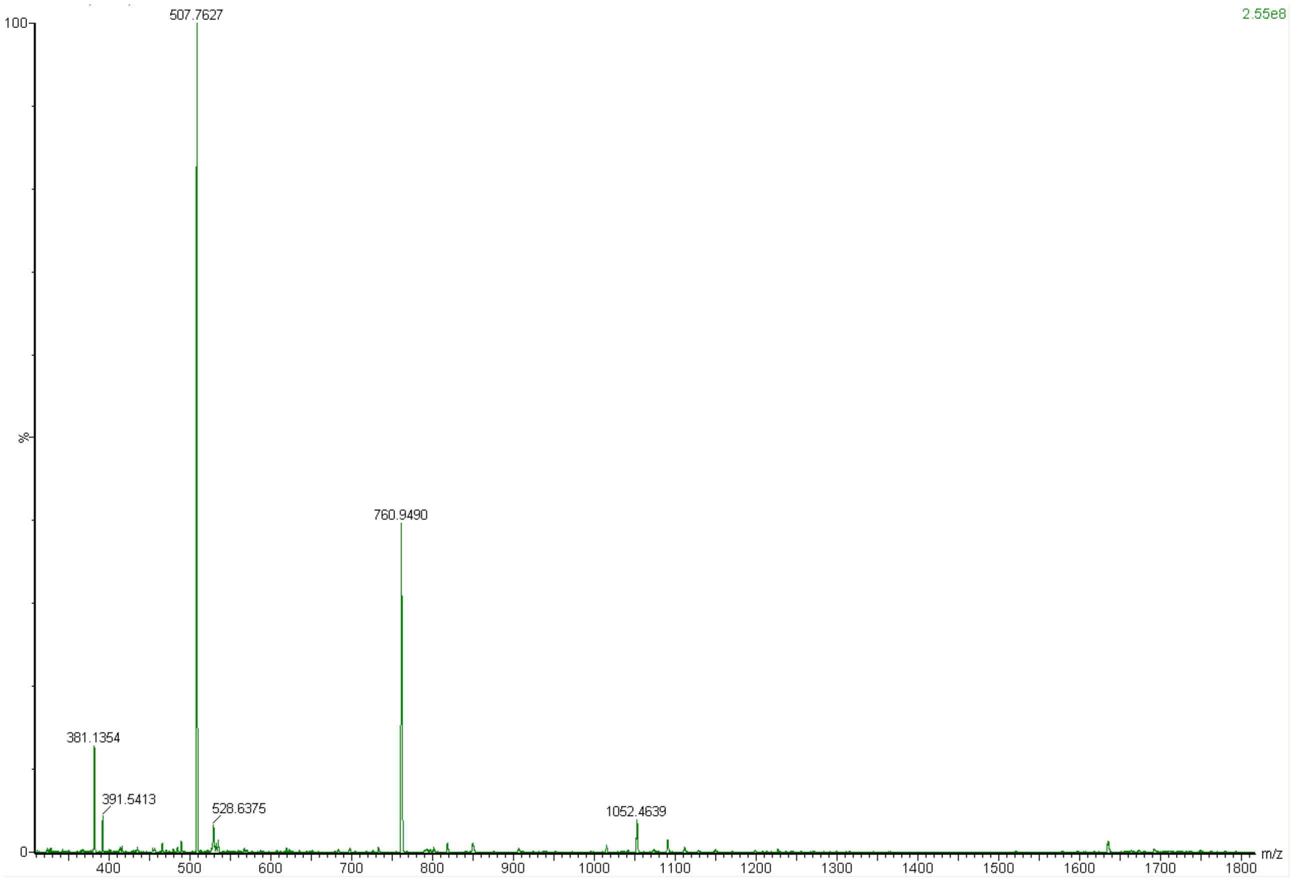

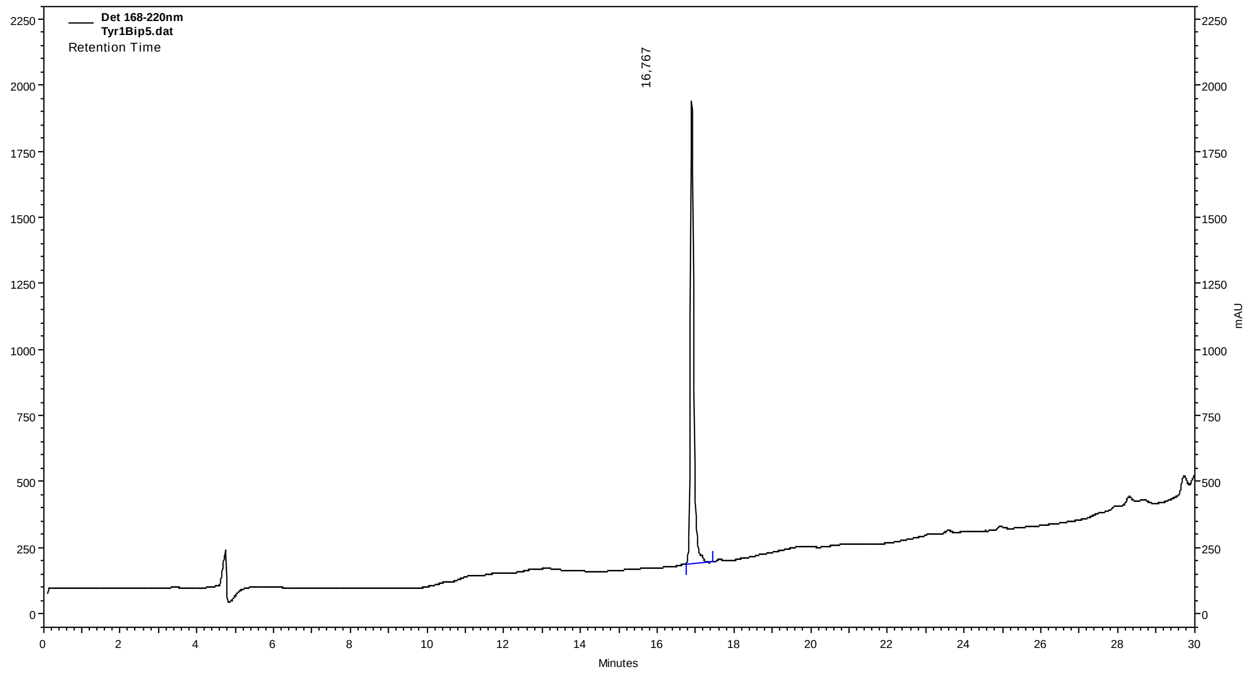

[Tyr[1],1Nal[5]]N/OFQ(1-13)NH_2

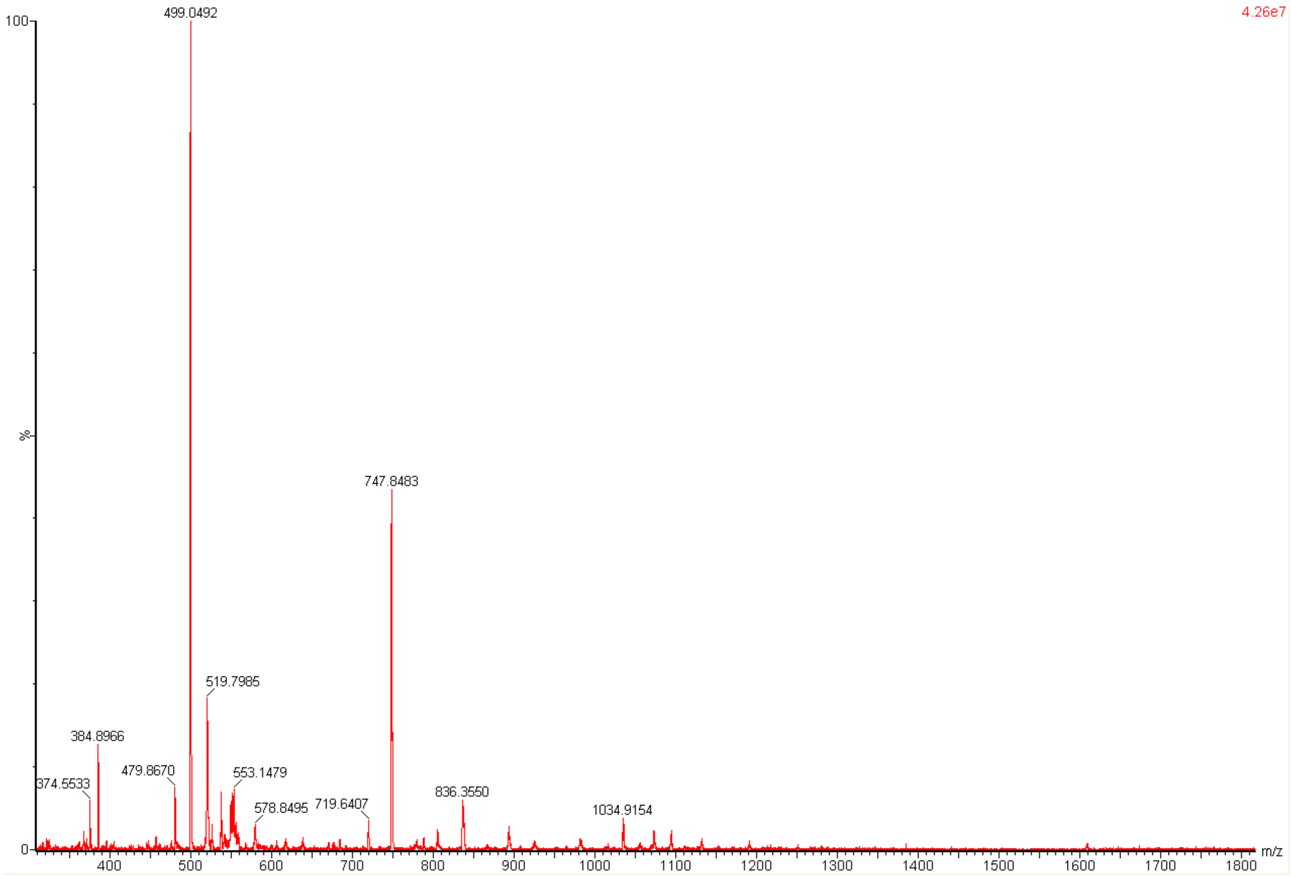

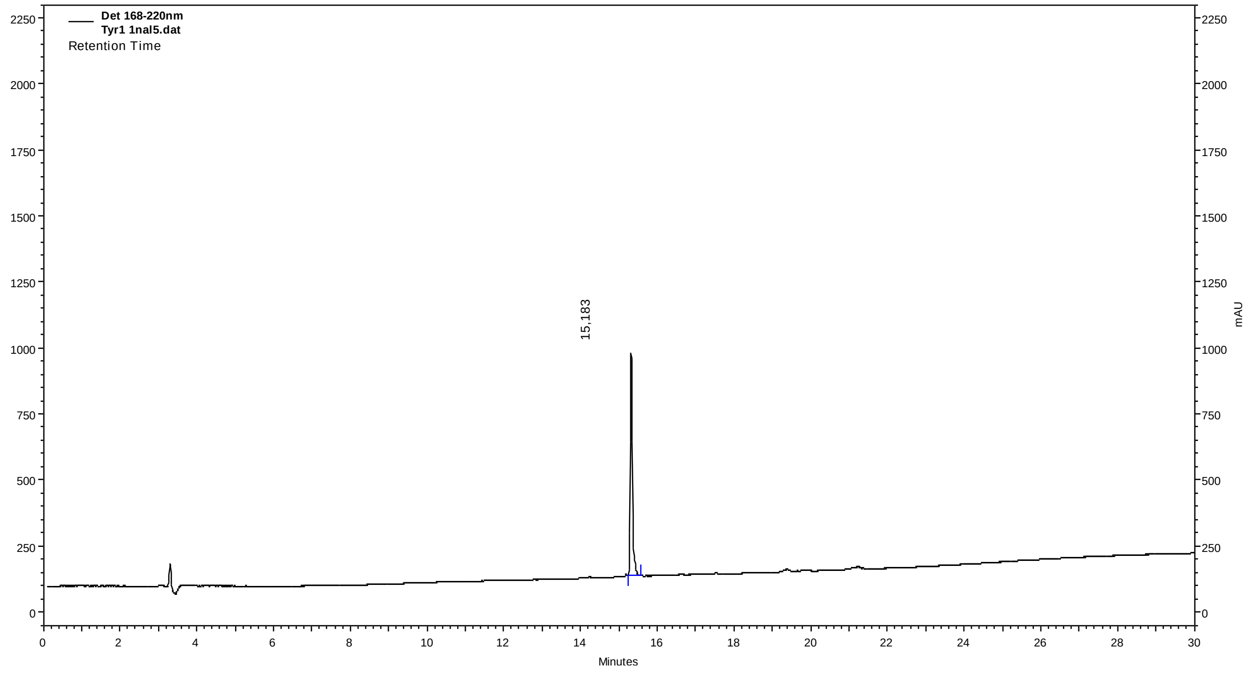

[Tyr[1],2Nal[5]]N/OFQ(1-13)NH_2

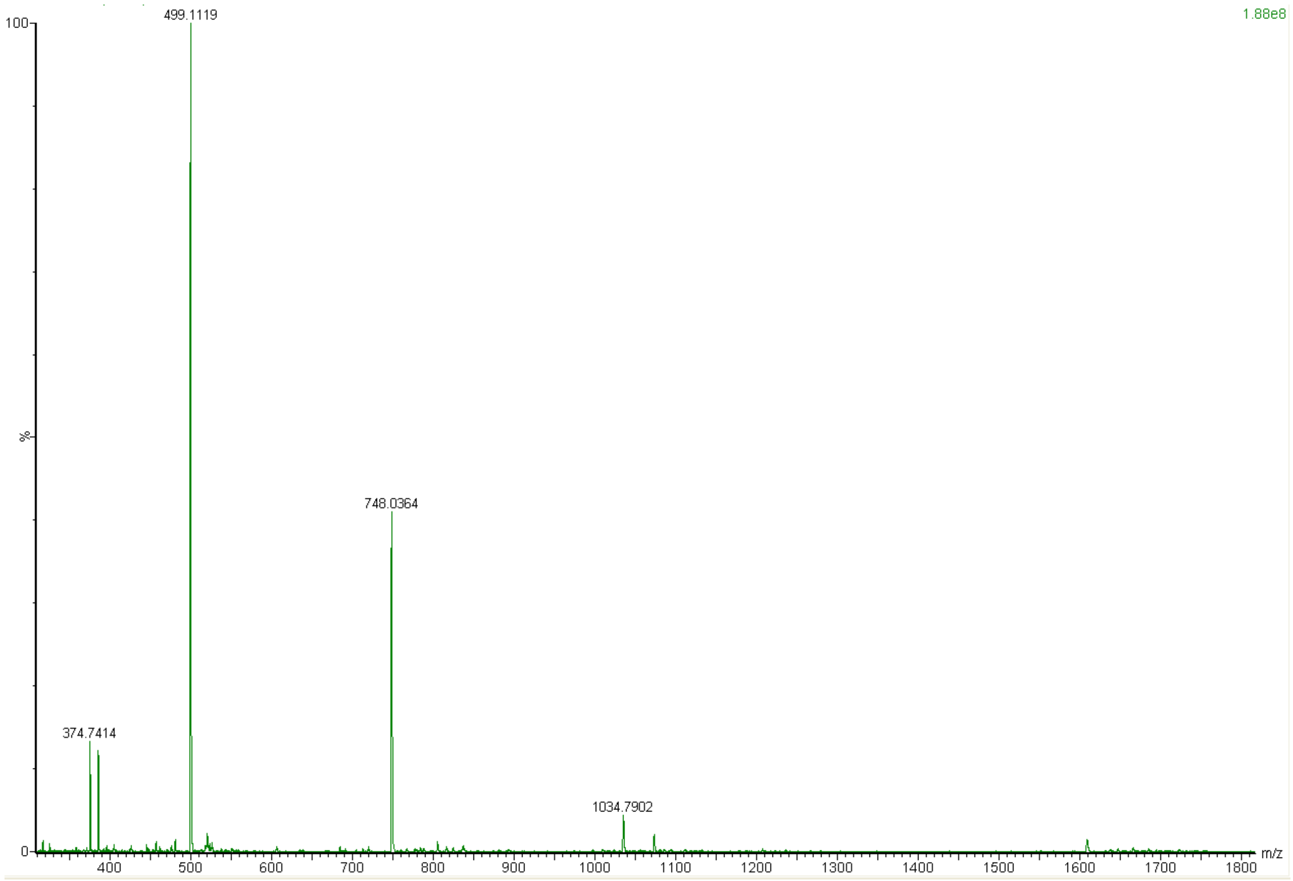

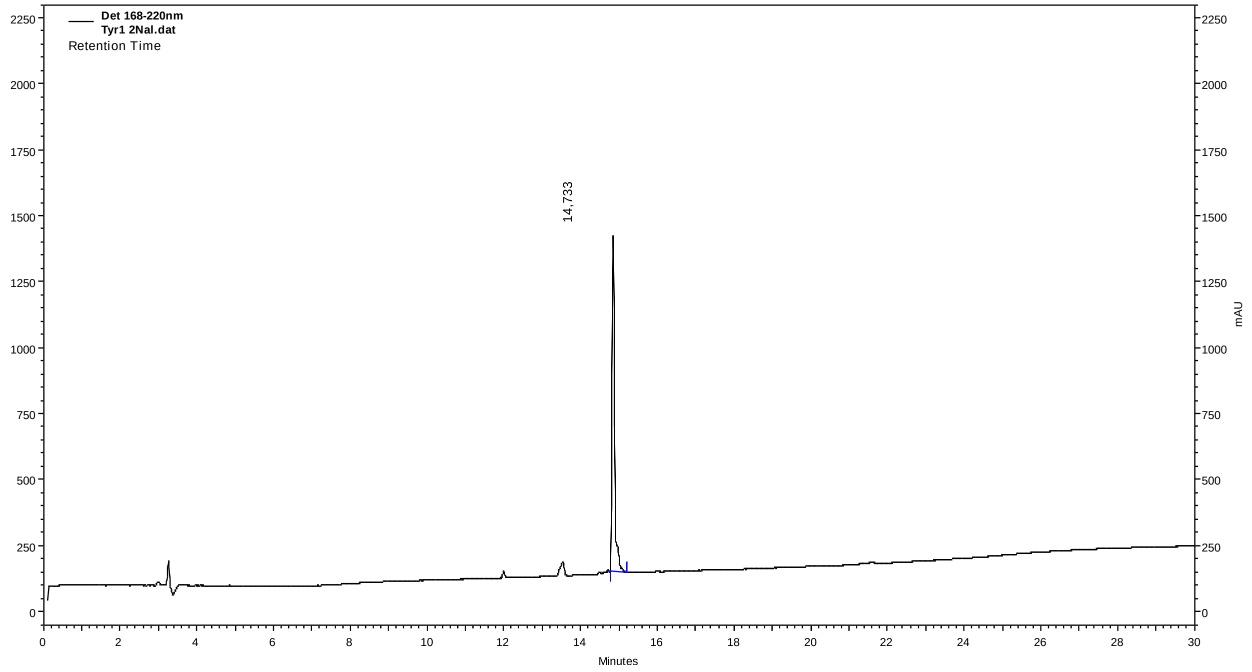

[Tyr[1],p(NH$_2$)Phe[5]]N/OFQ(1-13)NH$_2$

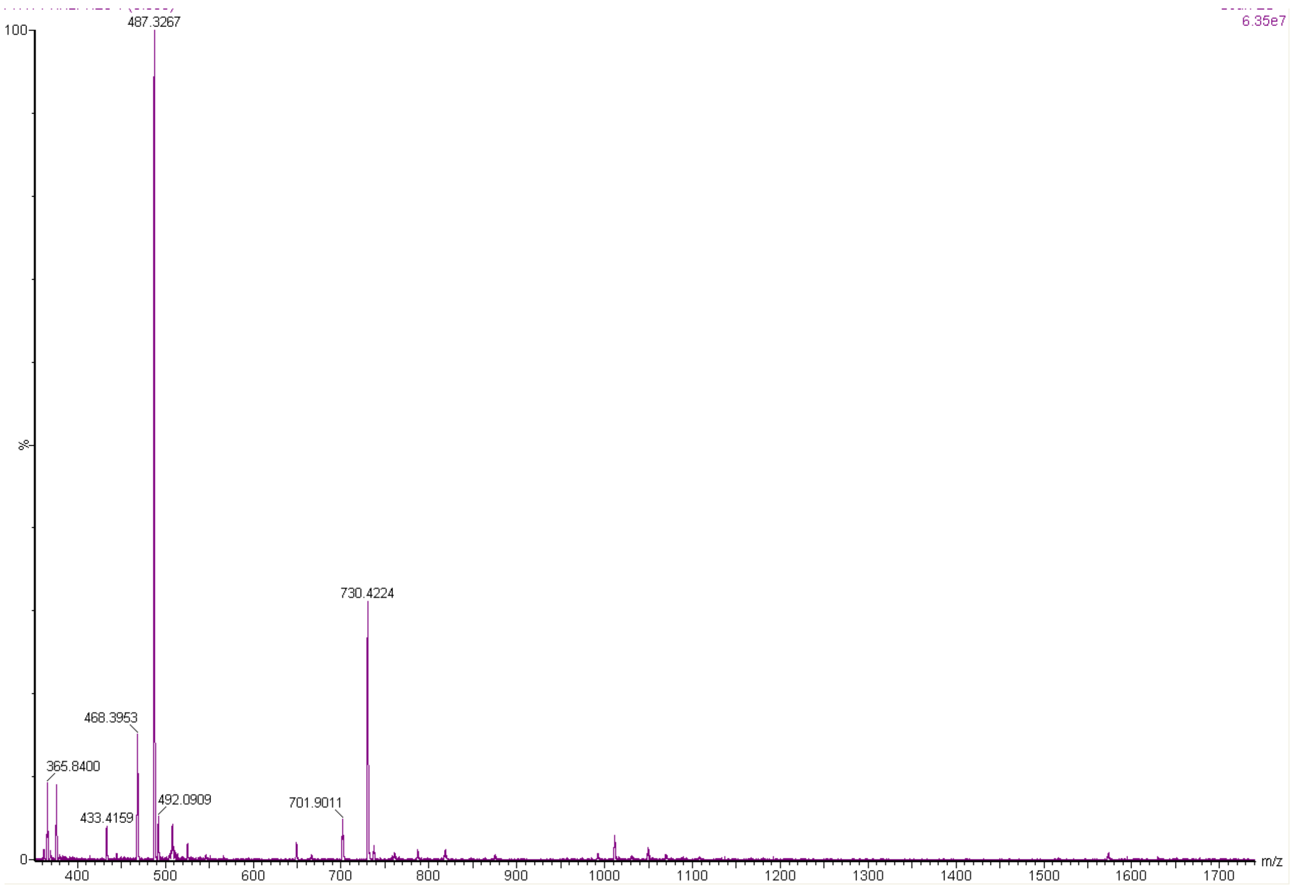

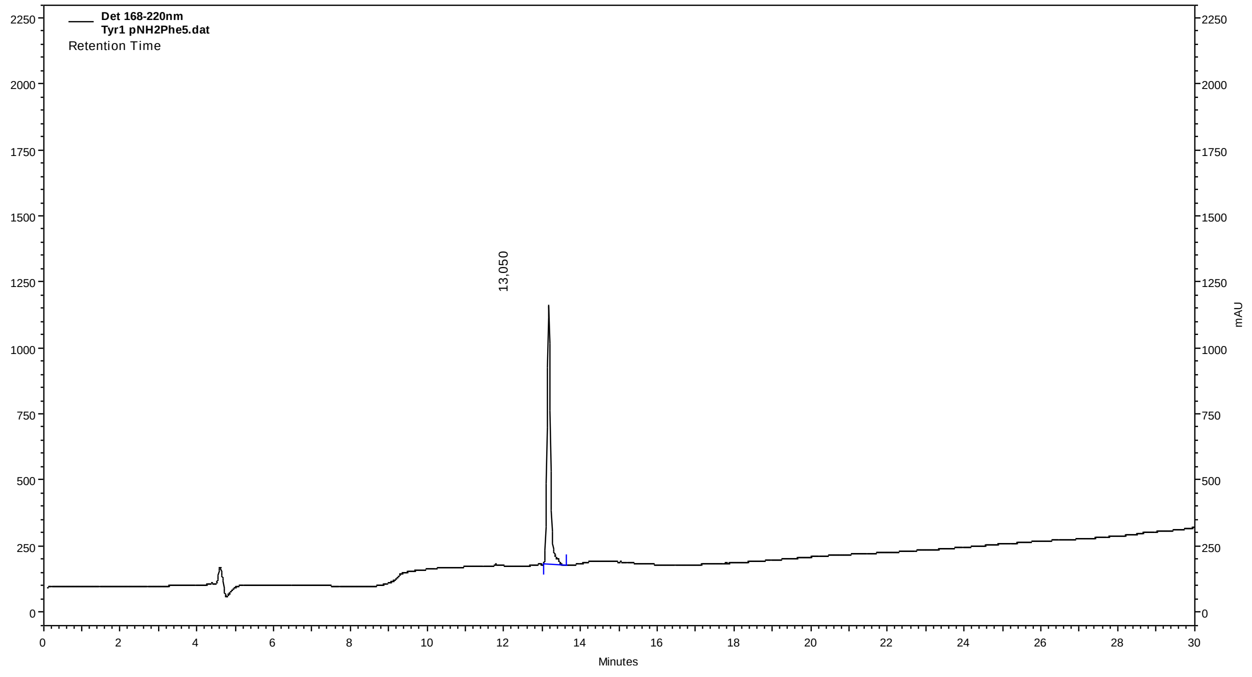

[Tyr[1],Dmt[5]]N/OFQ(1-13)NH_2

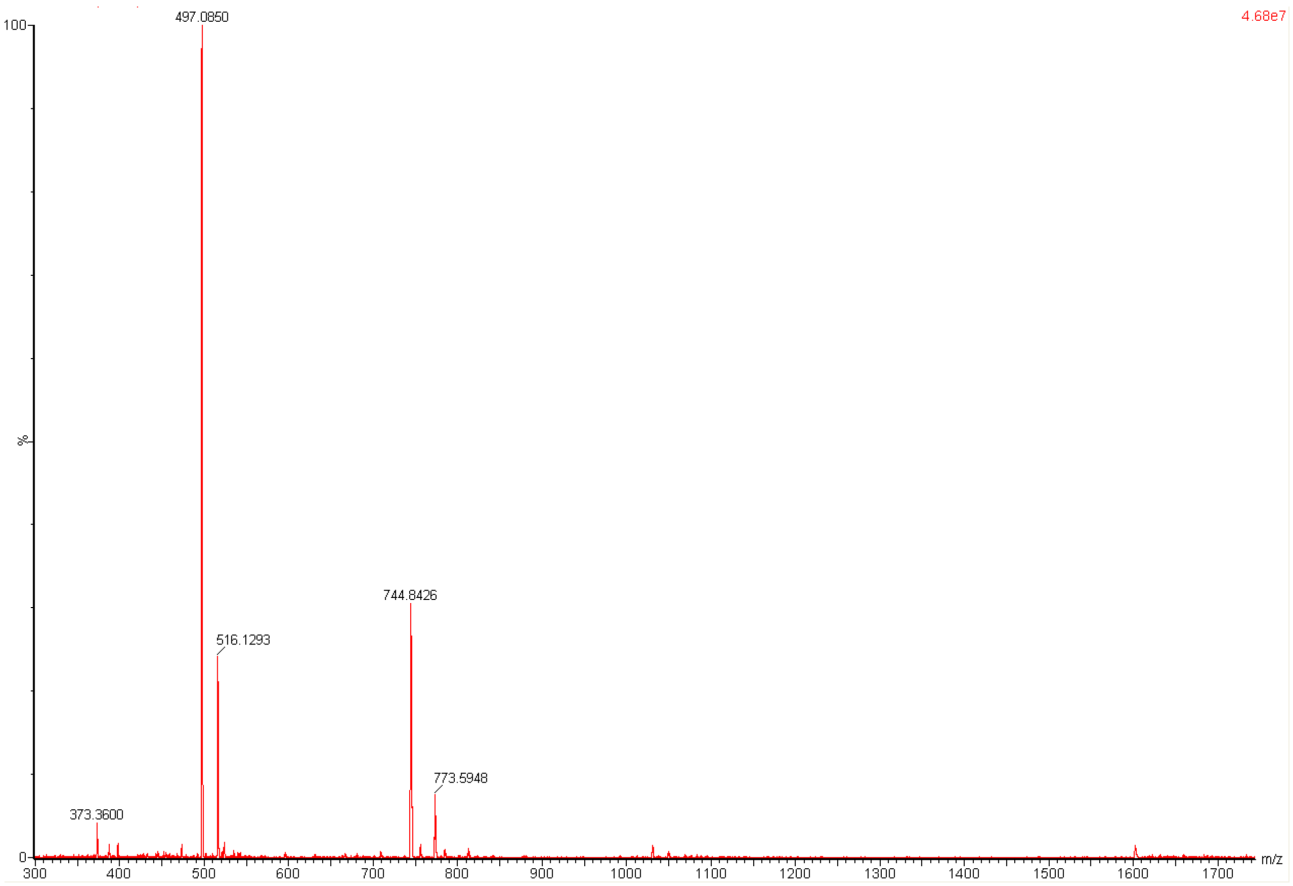

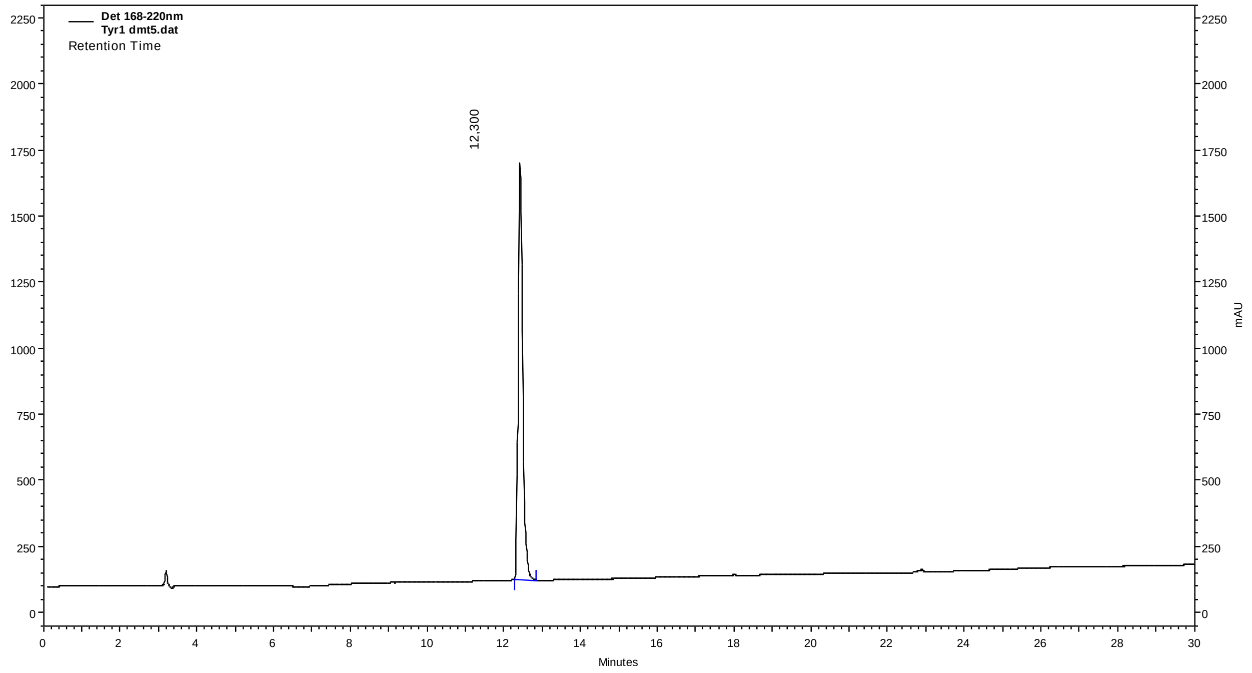

[Dmt¹,Phe⁵]N/OFQ(1-13)NH₂

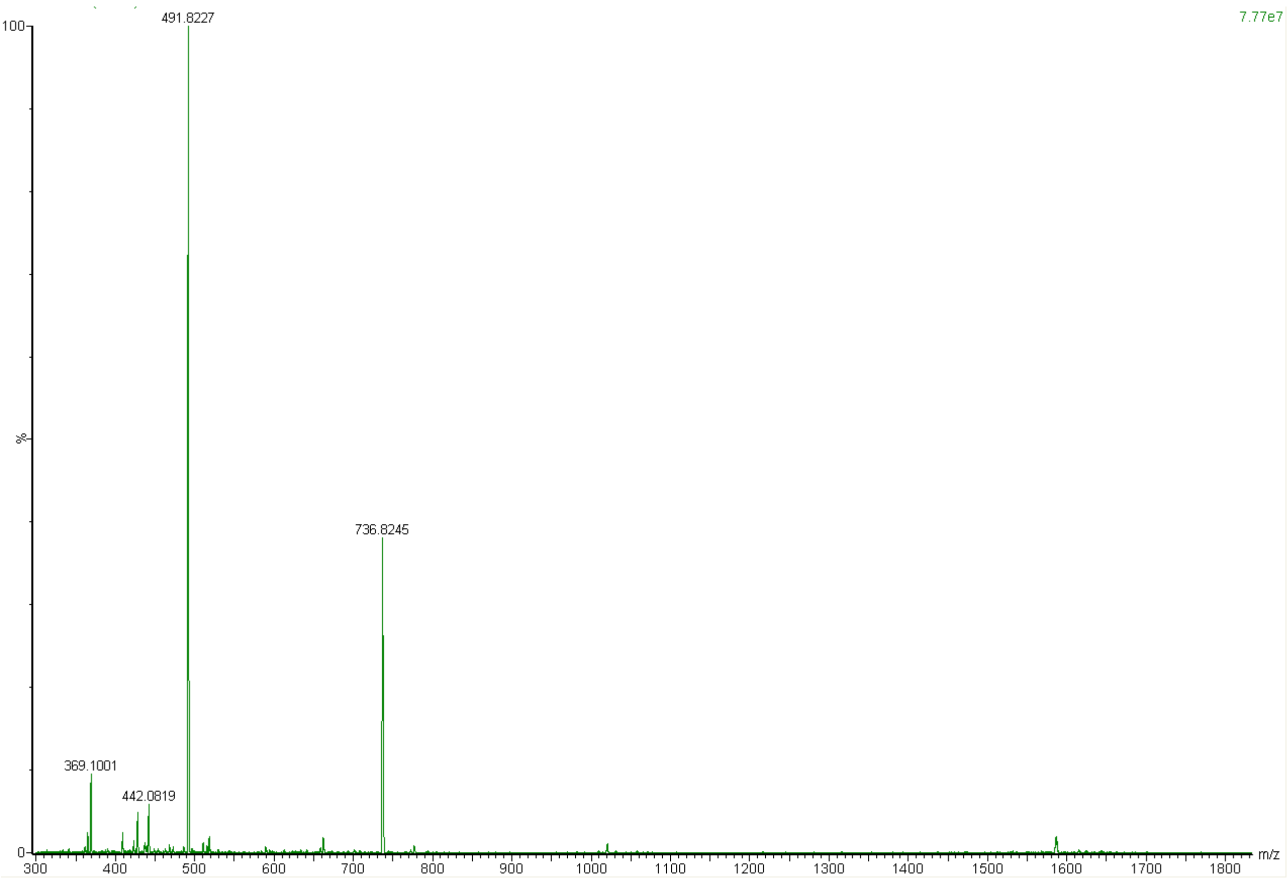

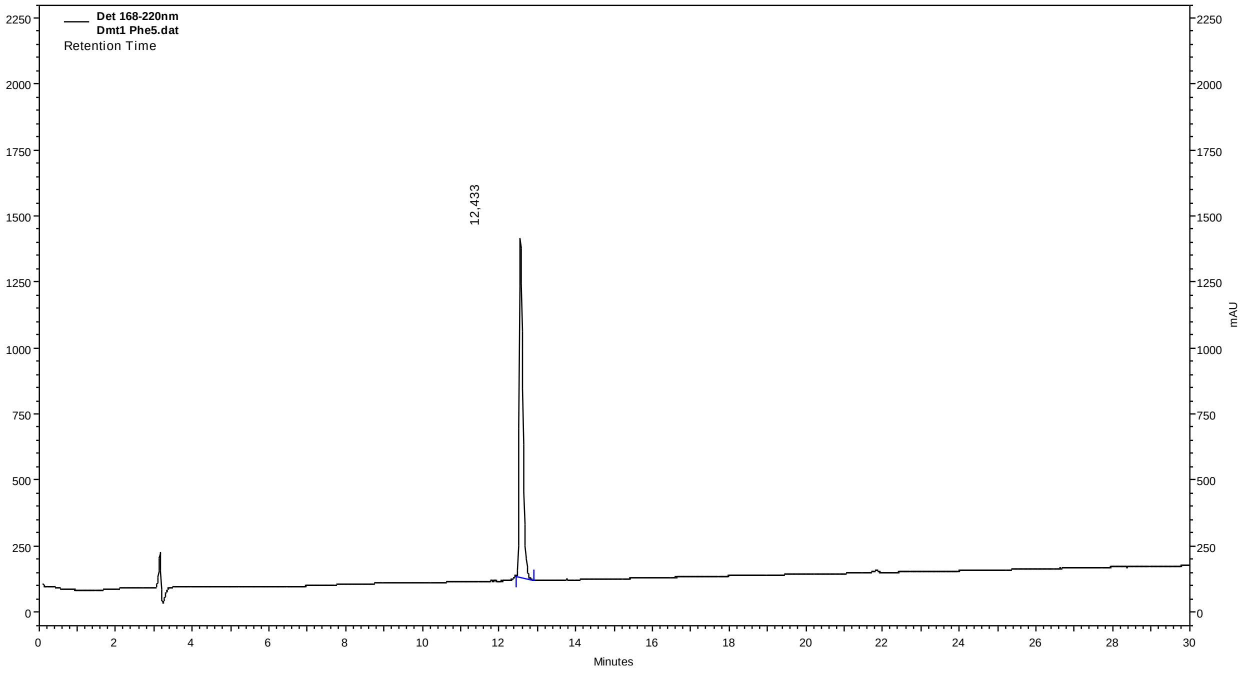

[Dmt[1],Phg[5]]N/OFQ(1-13)NH_2

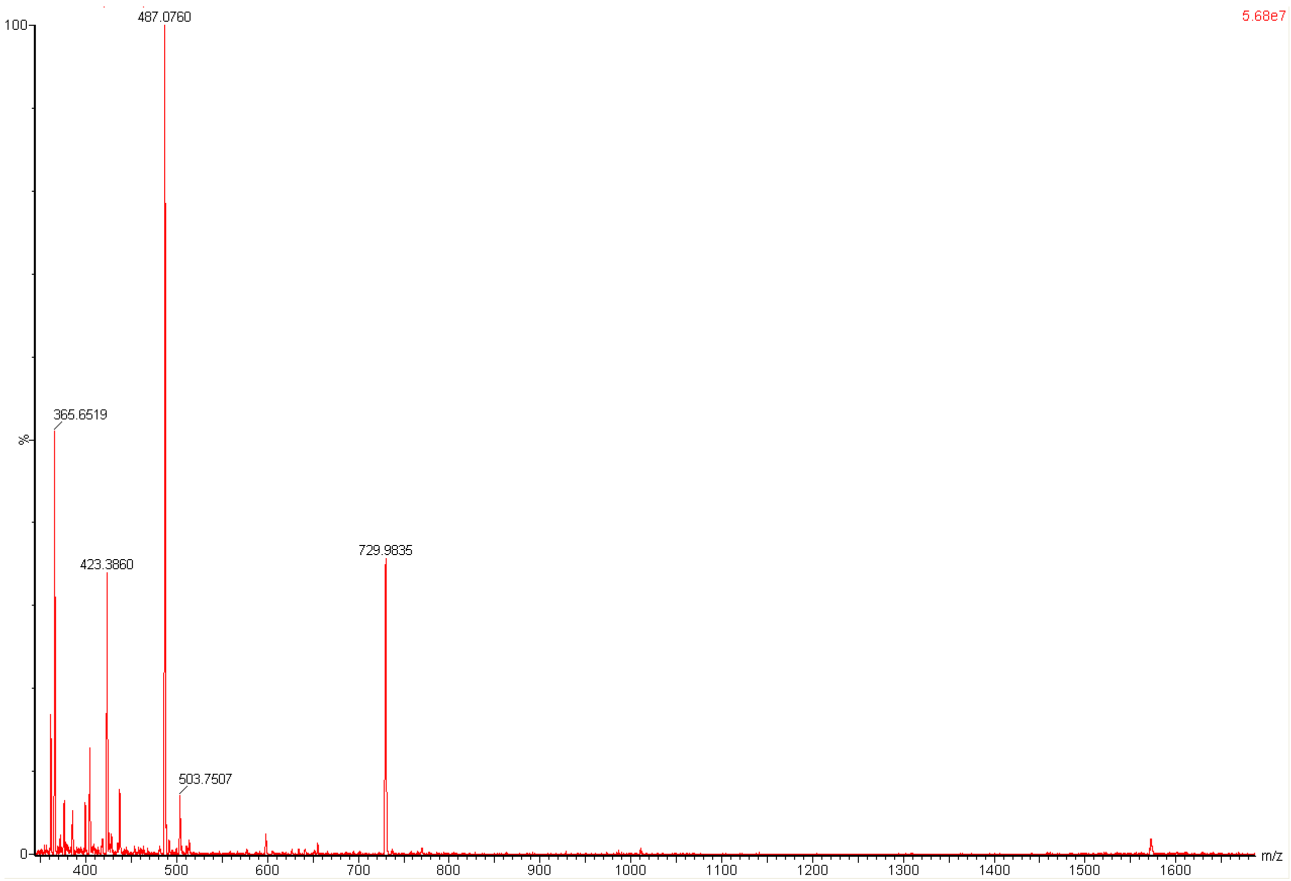

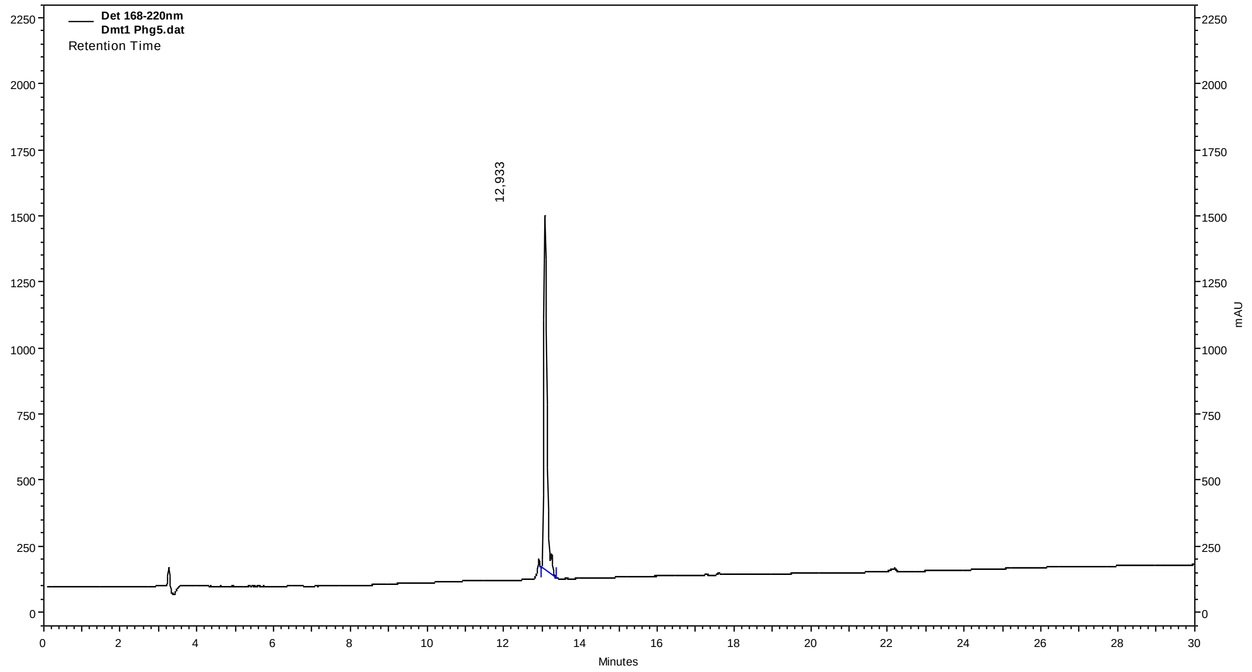

[Dmt[1],1Nal[5]]N/OFQ(1-13)NH$_2$

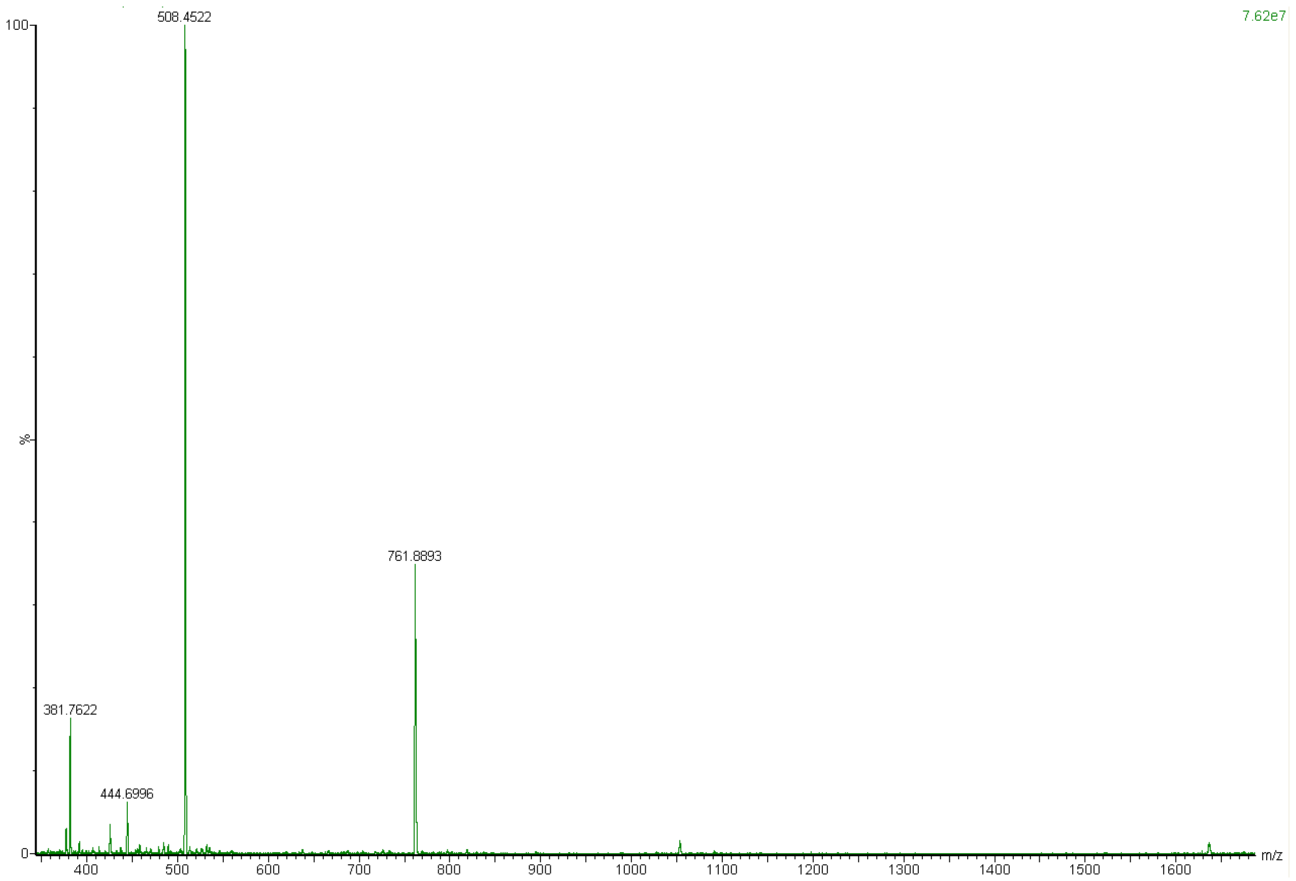

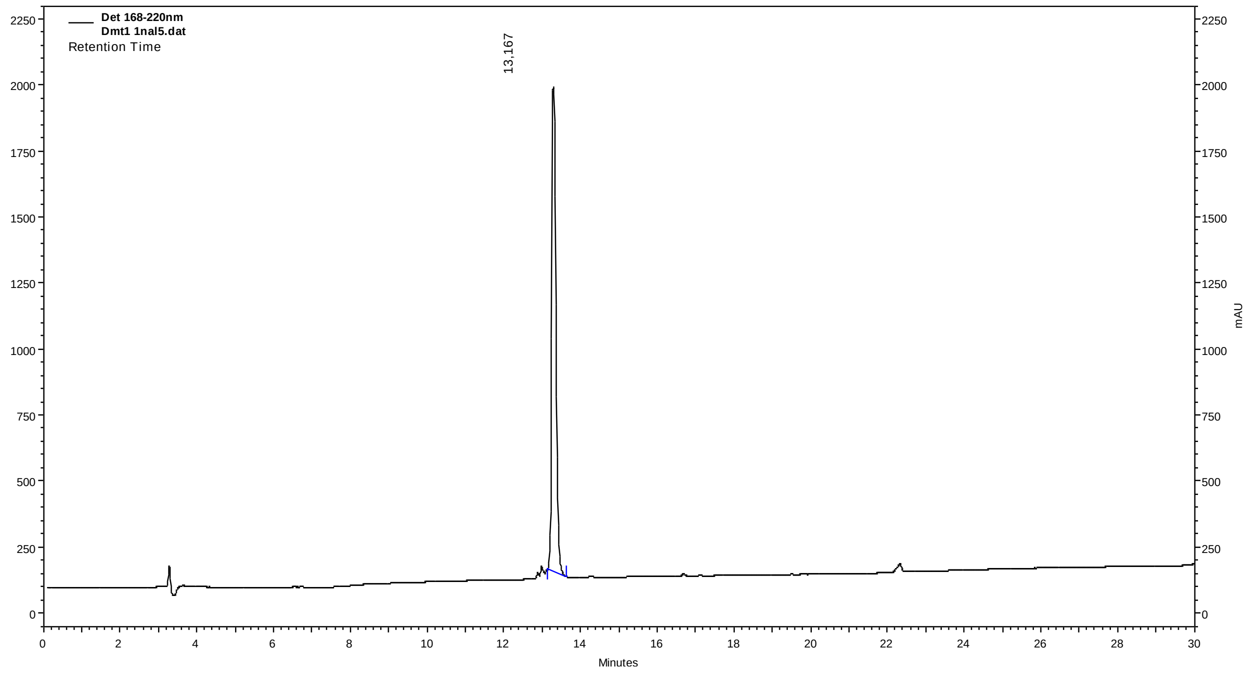

[Dmt^1,p(NH$_2$)Phe^5]N/OFQ(1-13)NH$_2$

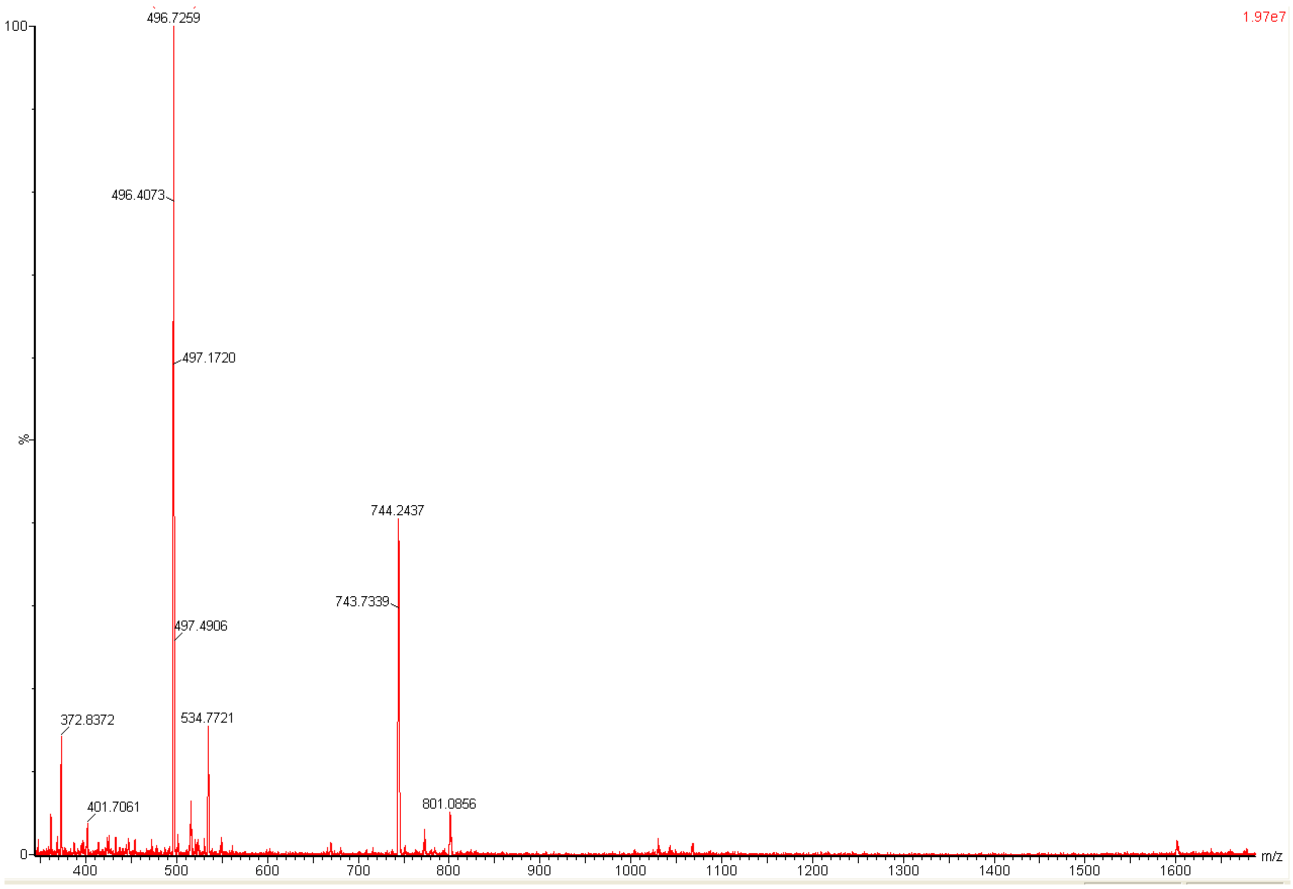

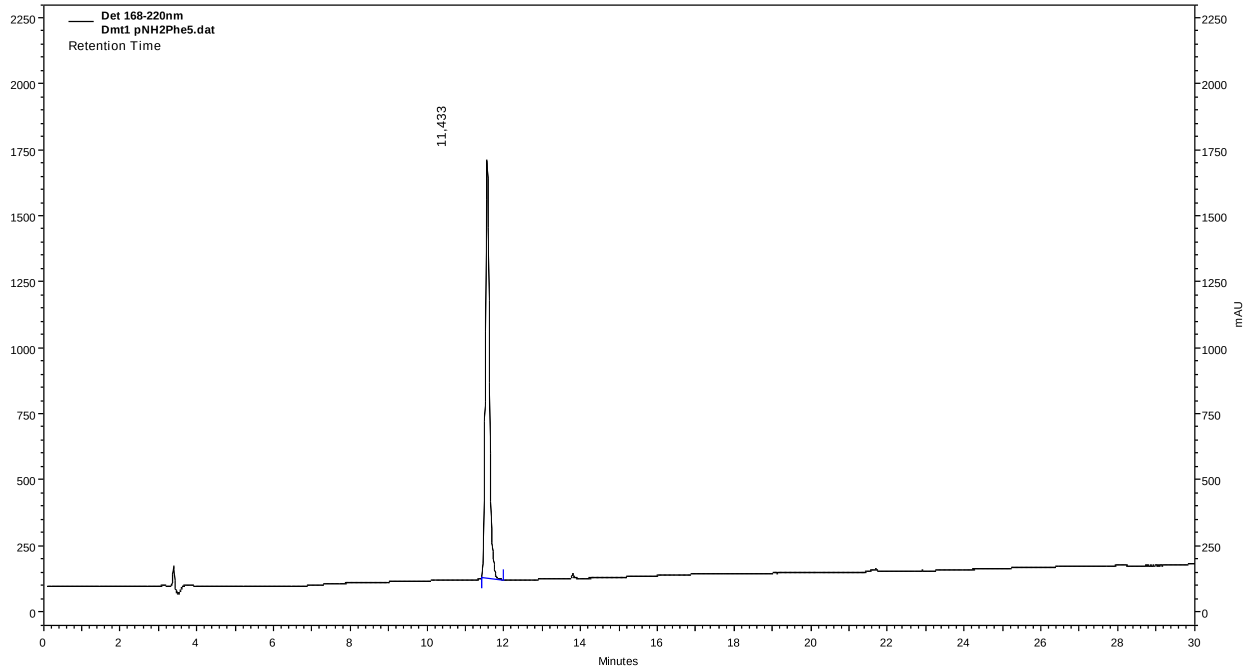

[Dmt[1,5]]N/OFQ(1-13)NH_2

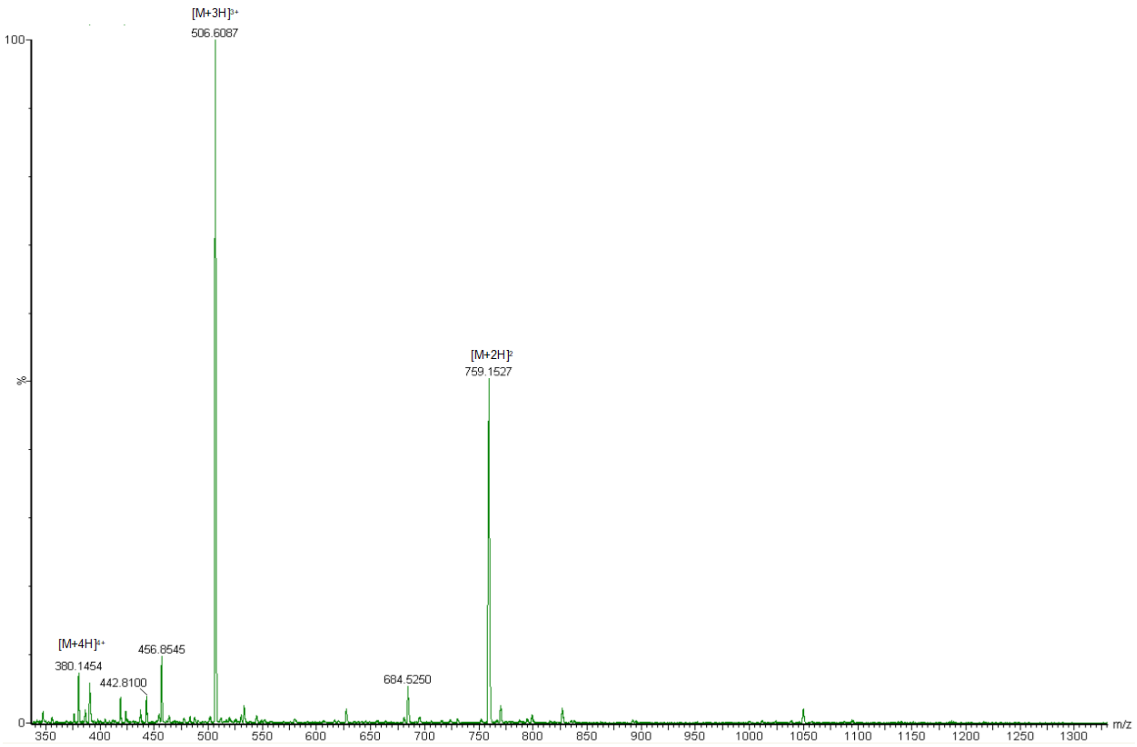

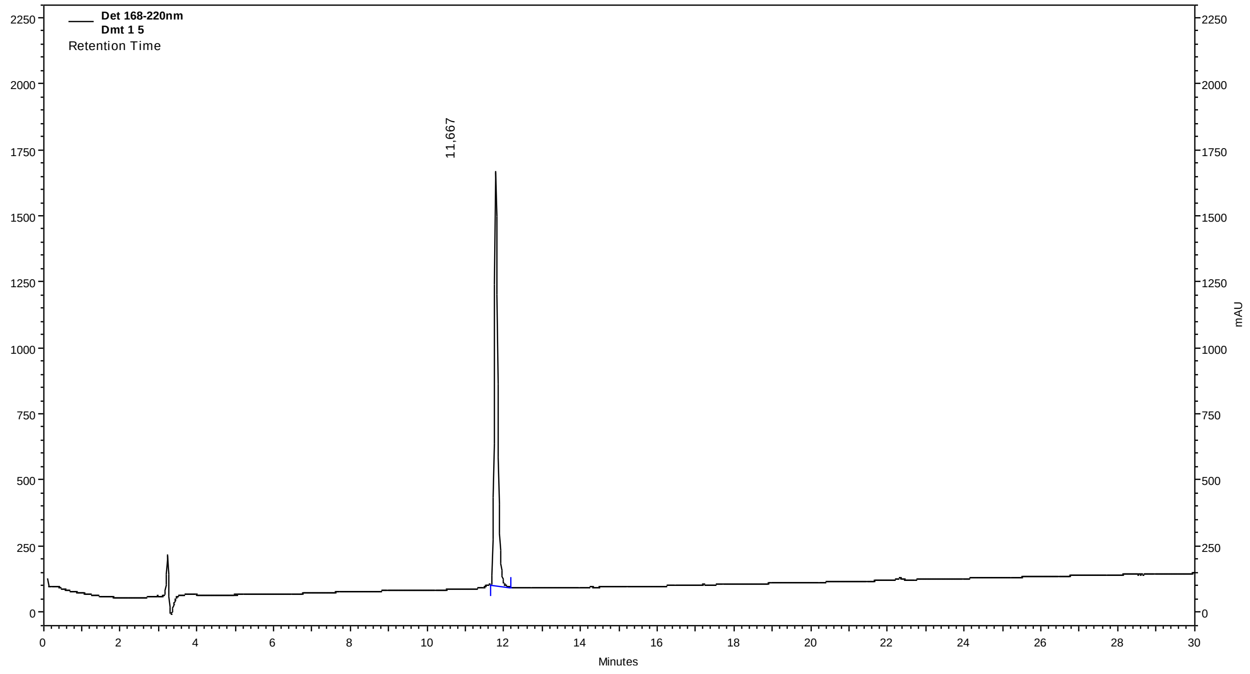

MOLECULAR DYNAMICS

Model set-up of non-natural peptides for MD. In order to perform MD simulation, the AMBER force field parameters and RESP atomic partial charges have been previously calculated for the non-natural residue dimethyl tyrosine (Dmt), which is not included in the standard AMBER residue libraries. Force fields and connectivity parameters were built via the Antechamber, Prepgen, Parmchk and Tleap modules of the freely distributed AMBER package of programs (Ambertools).[2] Calculations of the RESP atomic partial charges have been performed according to the method described by Cieplak et *al*.,[3] by first optimizing the structures and calculating a multi-conformation, multi-orientation QM electrostatic potential at the HF/6-31G* level for both the central fragment dipeptide (Dmt^5) and the N-terminal one (Dmt^1). The ORCA 4.0.1 package[4] was adopted for the QM calculations. Thereafter, Molden input files were generated by the ORCA module orca_2mkl for each calculated conformation and orientation, and used as input to the MULTIWFN program[5] for the final average and extraction of the RESP partial atomic charges via a 2-stage protocol including charge and equivalence constraints at both stages.

The peptides N/OFQ(1-9)-NH_2 and [Dmt^1Dmt^5]N/OFQ(1-9)-NH_2 were then built. The number of the aminoacids used to setup the peptides in the simulations (nine) is limited by the maximum number of torsions (32) that can be managed by the docking software.

Model set-up of the mu receptor. The initial structure of the mu receptor was set up starting from the 3.5 Å resolution cryo-electron microscopy structure of the receptor bound to the agonist peptide DAMGO and a nucleotide-free G_i protein (PDB code 6DDF).[6] Side-chain atoms missing in the reported structure due to structural disorder, were added and minimised "in vacuum" by using the "Modeller" and "Rotamer" modules in the Chimera UCSF package.[7]

Model set-up of the peptide-mu receptor complexes. The N/OFQ(1-9)-NH_2 and [$Dmt^{1,5}$]N/OFQ(1-9)-NH_2 peptides were docked using a "multi-flexible" docking procedure into the mu receptor as follows: a 600 ns MD trajectory of the overall mu-DAMGO-Gi-Protein water membrane system was produced; about 30 representative frames have been extracted from this trajectory, by a clustering algorithm as previously described.[8] These frames constitute the set of receptor conformations used in the docking procedure. The Smina program,[9] a fork for the Autodock Vina software package,[10,11] was used to extract binding poses according to three different scoring functions, namely "vina", "vinardo" and "ad4". All the non-amidic rotatable bonds within the peptides were allowed to rotate freely, whereas each one of the receptor conformations was considered rigid. The "exhaustiveness" parameter for a search space in 30x30x45 angs box was set to 24. For each receptor conformation, several results are produced which are merged, refined, clustered and sorted automatically to produce the final result, i.e. a small number of "interesting poses" or modes (parameter num_modes=9), within

an energy range of less than 3 kcal/mol (energy_range=3). Therefore, about 1000 "interesting poses" were produced adopting three scoring function and 30 receptor conformations for each peptide. All these results were combined with a consensus procedure allowing to select, after minimization and rescoring of the poses, the best one, according to either their average rank, or an exponential distribution function of their rank for each scoring function, as recently proposed.[12]

Analysis of molecular dynamics trajectories. Percentages of formation of hydrogen bonds, and 1st and 2nd order water bridges interactions along MD trajectories have been calculated by custom procedures using the MDAnalysis python library.[13] Polar and hydrophobic interactions between peptides and receptor residues were modelled as average coordination numbers via a continuous, differentiable switching function:

$$strength = n_{npc} = \sum_{ij} \frac{1-\left(r_{ij}/r_0\right)^a}{1-\left(r_{ij}/r_0\right)^b}$$

with the *i* and *j* indexes running over the interacting atoms within the peptide fragment of interest and those within a chosen receptor residue, with $0 \leq strength \leq n_i\, n_j$ (where n_i and n_j are the total number of atoms of the peptide fragment and the receptor residue, respectively, able to make the chosen type of interaction). For hydrophobic interactions, only carbon atoms are considered, for polar interactions only oxygens and nitrogens. The same "strength" function was calculated for salt bridges, π-cation and π-π interactions. In this case $n_i = n_j = 1$ because virtual atoms are defined at the center of mass of aromatic rings or charged groups, hence $0 \leq strength \leq 1$. For hydrophobic, salt bridges, π-cation and π-π interactions a=6, b=12 and r_0=6 Å, 5 Å, 4 Å, 5.5 Å respectively, while for polar interactions a=8, b=12, r_0=2.5. The chosen values of r_0 account for the typical interaction distance plus thermal motion's amplitude (e.g. ~4.5 Å + ~1.5 Å in the case of hydrophobic interactions). The Plumed package[14] was patched to the Gromacs engine to undergo this kind of analysis. These coordination numbers (one per residue of the binding site) can be calculated on single structures as well as averaged along trajectories.

Beyond the roughness of the approach used here to monitor π–π and π-cation interactions (based only on the distances between c.o.m.), a careful characterization of them would require quantum mechanics. However it is known that classical molecular dynamics using fixed-charge force fields has been successfully applied to describe π-stacking in both parallel and T-shaped configurations;[15,16] on the other hand π-cation interactions are considered a shortcoming of most of fixed-charge force fields and their mention in this article emerges only by "a posteriori" analysis of distances between positive charges and aromatic rings.

Figure S1. Analysis of the 600ns MD trajectory of the mu receptor-DAMGO-Gi protein complex (starting structure from PDB code: 6DDF). **A)** RMSD plot of heavy atoms of DAMGO. Side frame: RMSD distribution of DAMGO. **B)** Fractions of the main conformations of DAMGO found by clustering. **C)** The three main conformations extracted.

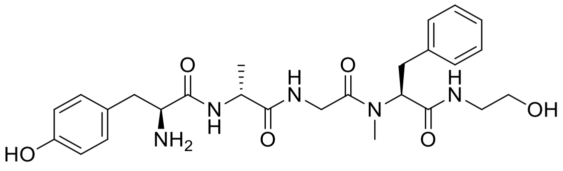

DAMGO

A)

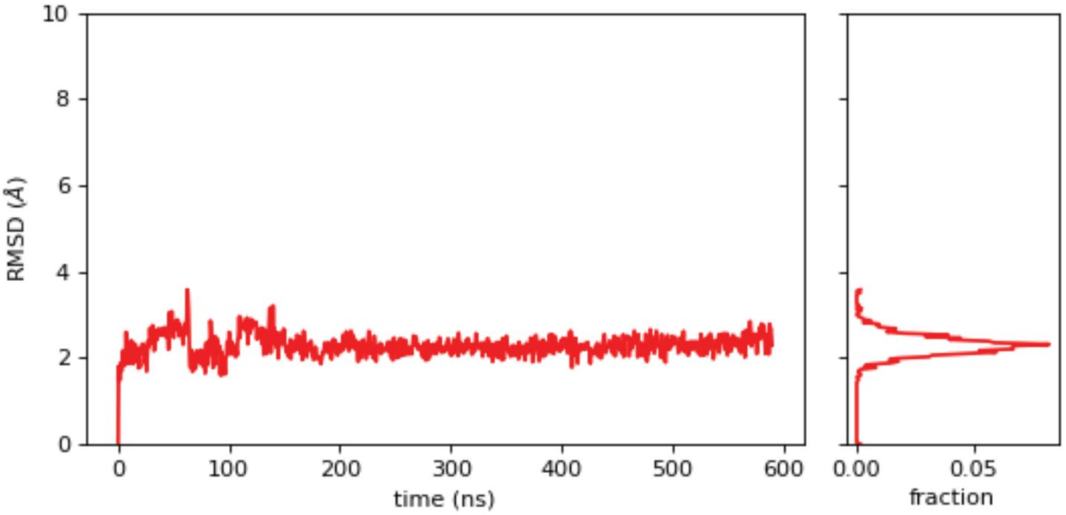

B)

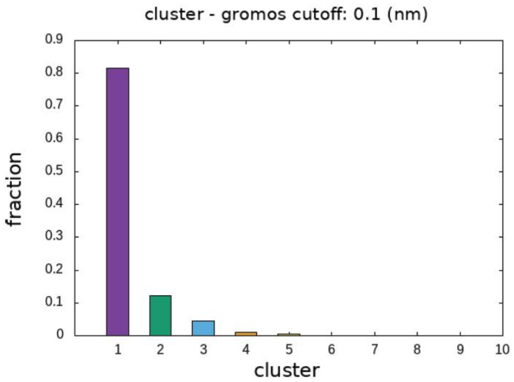

C)

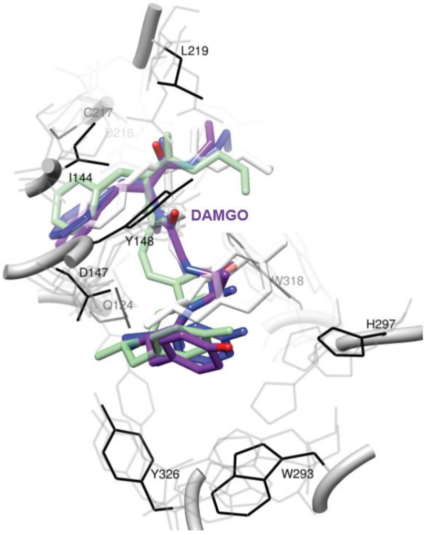

Figure S2. Analysis of the 2 MD trajectories of the mu receptor-[Dmt[1,5]]N/OFQ(1-9)-NH_2 complex. **A)** RMSD plots of heavy atoms of residues 1-5 of the peptide. Side frames: RMSD distributions. **B)** Fractions of the main conformations found of the peptide by clustering. **C)**
The three main conformations extracted.

A)

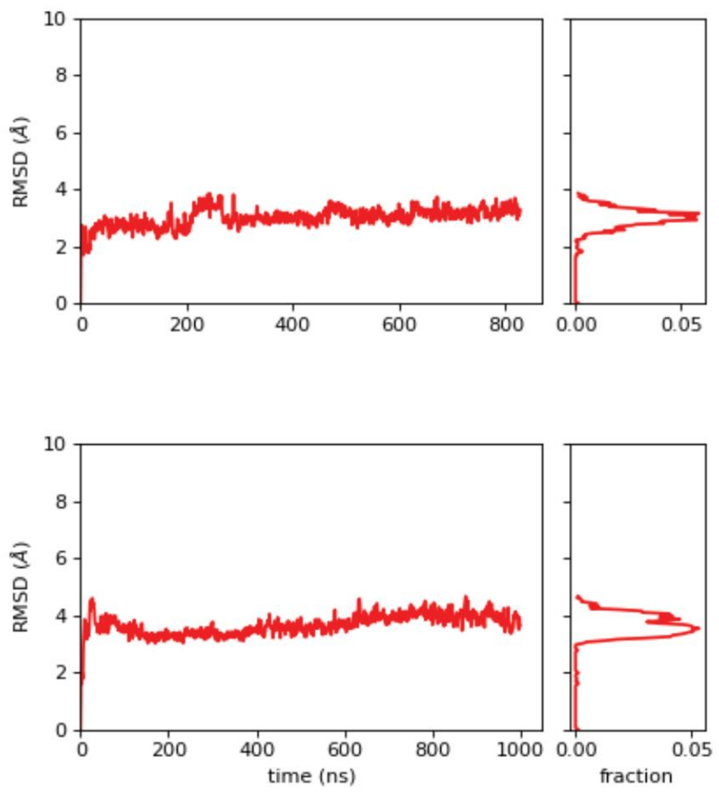

B)

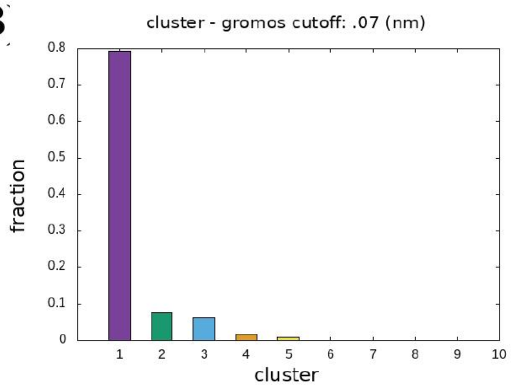

C)

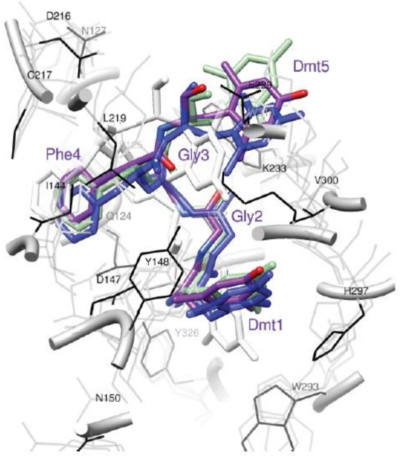

Figure S3. Analysis of the two MD trajectories of the N/OFQ(1-9)-NH_2-mu receptor complex. **A)** RMSD plots of heavy atoms of residues 1-5 of N/OFQ(1-9)-NH_2. Side frames: RMSD distributions. **B)** Fractions of the main conformations found for the peptide by clustering. **C)** The best conformation (purple) extracted compared with DAMGO (yellow).

A)

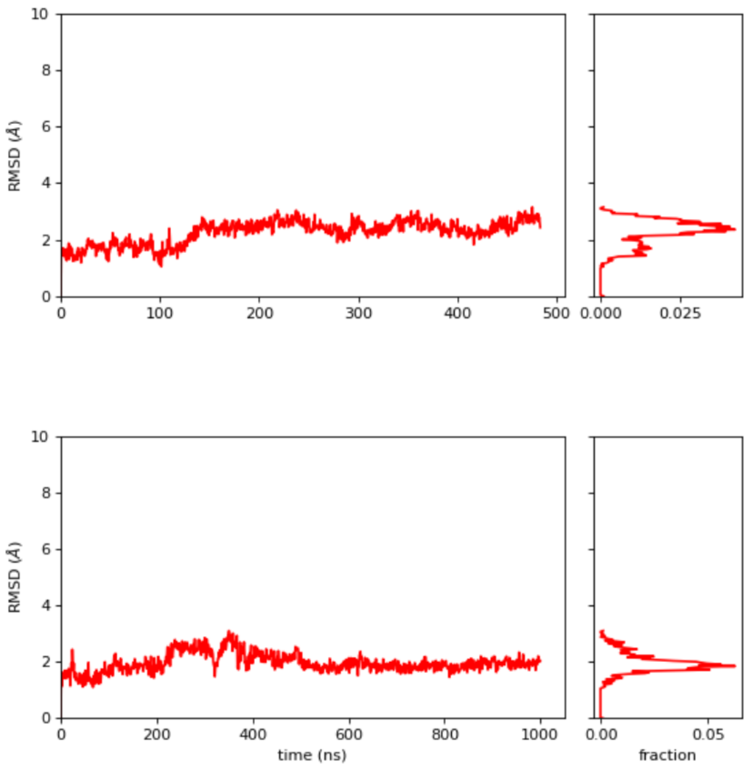

B)

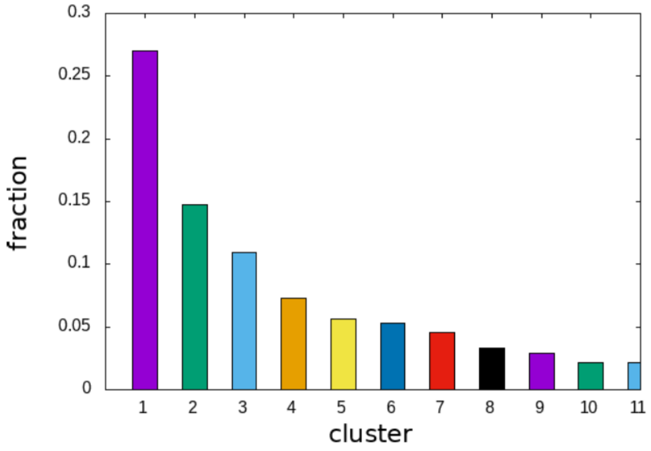

C)

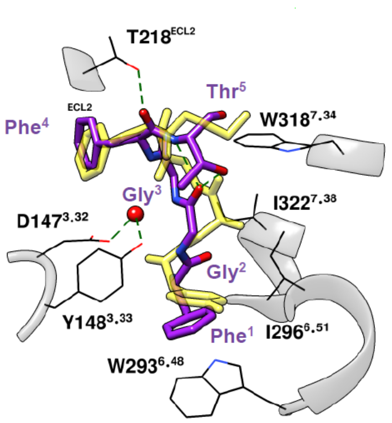

REFERENCES

1) Wang, X., Niu, S., Xu, L., Zhang, C., Meng, L., Zhang, X., Ma, D. Pd-Catalyzed Dimethylation of Tyrosine-Derived Picolinamide for Synthesis of (S)-N-Boc-2,6-dimethyltyrosine and Its Analogues. *Org Lett* **2017,** *19*, 246-249.
2) Case, D.A., I.Y. Ben-Shalom, S.R. Brozell, D.S. Cerutti, T.E. Cheatham, III, V.W.D. Cruzeiro, T.A. Darden, R.E. Duke, D. Ghoreishi, M.K. Gilson, H. Gohlke, A.W. Goetz, D. Greene, R Harris, N. Homeyer, S. Izadi, A. Kovalenko, T. Kurtzman, T.S. Lee, S. LeGrand, P. Li, C. Lin, J. Liu, T. Luchko, R. Luo, D.J. Mermelstein, K.M. Merz, Y. Miao, G. Monard, C. Nguyen, H. Nguyen, I. Omelyan, A. Onufriev, F. Pan, R. Qi, D.R. Roe, A. Roitberg, C. Sagui, S. Schott-Verdugo, J. Shen, C.L. Simmerling, J. Smith, R. Salomon-Ferrer, J. Swails, R.C. Walker, J. Wang, H. Wei, R.M. Wolf, X. Wu, L. Xiao, D.M. York and P.A. Kollman (**2018**), AMBER 2018, University of California, San Francisco.
3) Cieplak, P., W.D. Cornell, C. Bayly & P.A. Kollman, Application of the multimolecule and multiconformational RESP methodology to biopolymers: Charge derivation for DNA, RNA, and proteins. *J Comput Chem* **1995**, *16*, 1357-1377.
4) Neese, F., The ORCA program system, *Wires Computational Molecular Science, Wiley On line Library*, **2011**, https://doi.org/10.1002/wcms.81.
5) Lu, T., F. Chen, Multiwfn: A multifunctional wavefunction analyzer, *J Comput Chem* **2012**, *33*, 580-592.
6) Koehl, A., H. Hu, S. Maeda, Y. Zhang, Q. Qu, J.M. Paggi, N.R. Latorraca, D. Hilger, R. Dawson, H. Matile, G.F.X. Schertler, S. Granier, W.I. Weis, R.O. Dror, A. Manglik, G. Skiniotis and B.K. Kobilka, Structure of the μ-opioid receptor–Gi protein complex. *Nature* **2018**, *558*, 547-552.
7) Pettersen, E. F., Goddard, T. D.,Huang, C. C., Couch, G. S., Greenblatt, D. M., Meng, E. C., Ferrin, T. E., UCSF Chimera-a Visualization System for Exploratory Research and Analysis. *J Comput Chem* **2004**, *25*, 1605-1612.
8) Della Longa, S., Arcovito, A., Microswitches for the Activation of the Nociceptin Receptor Induced by Cebranopadol: Hints from Microsecond Molecular Dynamics, *J Chem Inf Model* **2019**, *59*, 818−831.
9) Koes, D.R., Baumgartner MP, Camacho CJ. Lessons learned in empirical scoring with smina from the CSAR 2011 benchmarking exercise. *J Chem Inf Model* **2013**, *53*, 1893–1904.
10) Morris, G. M., Huey, R., Lindstrom, W., Sanner, M. F., Belew, R. K., Goodsell, D. S., Olson, A. J., AutoDock4 and AutoDockTools4: Automated Docking with Selective Receptor Flexibility. *J Comput Chem* **2009**, *30*, 2785-2791.
11) Trott, O., Olson, A. J., AutoDock Vina: Improving the Speed and Accuracy of Docking with a New Scoring Function, Efficient Optimization, and Multithreading. *J Comput Chem* **2010**, *31*, 455-461.
12) Palacio-Rodríguez, K., Lans I, Cavasotto CN, Cossio P, Exponential consensus ranking improves the outcome in docking and receptor ensemble docking. *Sci Rep* **2019**, *9*, 5142.
13) Michaud-Agrawal, N., E. J. Denning, T. B. Woolf, and O. Beckstein. MDAnalysis: A Toolkit for the Analysis of Molecular Dynamics Simulations. *J Comput Chem* **2011**, *32*, 2319-2327.
14) Tribello, G. A., Bonomi, M., Branduardi, D., Camilloni, C., and Bussi, G. Plumed 2.0: new feathers for an old bird. *Comput Phys Commun* **2014**, *185*, 604-613.
15) Macias AT, MacKerell AD. CH/pi interactions involving aromatic amino acids: refinement of the CHARMM tryptophan forcefield. *J Comput Chem* **2005**, *26*, 1452–1463.
16) Kang M., Zhang P.,Cui H., and Loverde S.M. π–π Stacking Mediated Chirality in Functional Supramolecular Filament. *Macromolecules* **2016**, *49*, 994-1001.
